# Prevalence of physical frailty and impact on survival in patients with chronic kidney disease: a systematic review and meta-analysis

**DOI:** 10.1186/s12882-023-03303-1

**Published:** 2023-09-03

**Authors:** Fan Zhang, Hui Wang, Yan Bai, Ying Zhang, Liuyan Huang, Huachun Zhang

**Affiliations:** 1 Department of Nephrology, Longhua Hospital Shanghai University of Traditional Chinese Medicine, Shanghai, China; 2 Department of Anorectology, Longhua Hospital Shanghai University of Traditional Chinese Medicine, Shanghai, China; 3 Department of Cardiology, Longhua Hospital Shanghai University of Traditional Chinese Medicine, Shanghai, China; 4 Department of Surgery, Longhua Hospital Shanghai University of Traditional Chinese Medicine, Shanghai, China; 5 Department of Nursing, Longhua Hospital Shanghai University of Traditional Chinese Medicine, Shanghai, China

**Keywords:** Physical frailty, Chronic kidney disease, Mortality, Systematic review, Meta-analysis

## Abstract

**Background:**

Frailty is common in chronic kidney disease (CKD) patients and becomes more prevalent as kidney disease progresses. This study aimed to investigate the prevalence of physical frailty and quantify the relationship between frailty and mortality risk in patients with CKD.

**Methods:**

PubMed, Web of Science, Embase, Cochrane Central Register of Controlled Trials, Clinicaltrial.gov, and major renal academic conferences were systematically searched, and additional references to relevant articles were manually searched. The prevalence of physical frailty and the risk of mortality based on random-effects models were assessed using percentages and hazard ratio (HR) with a 95% confidence interval (CI).

**Results:**

A total of 139 articles, including 1,675,482 participants, met the eligibility criteria for the meta-analysis. The results showed that 34.5% (95% CI 31.0 to 38.1%) of CKD patients showed signs of frailty, and 39.4% (95% CI 35.4 to 43.5%) had prefrail symptoms. Compared to non-frail patients, the risk of mortality was increased by 94.1% (95% CI 1.586 to 2.375) in frail patients and 34.5% (95% CI 1.231 to 1.469) in prefrail patients.

**Conclusion:**

The high prevalence of frailty and prefrail in adults with CKD and resulting in premature death emphasize the importance of measuring frailty, which provides important prognostic information and may provide opportunities for interventions to improve the prognosis of patients with CKD.

**Supplementary Information:**

The online version contains supplementary material available at 10.1186/s12882-023-03303-1.

## Introduction

Chronic kidney disease (CKD) is one of the leading causes of increased cardiovascular morbidity and mortality, [1] which is associated with higher healthcare costs and significantly burden. [2] Currently, CKD has become a growing public health problem. [3]

Frailty is a state of increased vulnerability to stressors due to decreased physiological reserves and thus leads to a poor health outcome, [4] and it is described as a spectrum from no frailty (i.e., robustness) to pre-frailty (i.e., early stage of frailty) and then physical frailty. [5] In the context of healthy aging, physical frailty was considered a public health priority, and its complexity requires specific management strategies. [6]

Physical frailty is common in patients with CKD, [7, 8] which can lead to a poor prognosis, as reflected by an increased risk of adverse events such as motor dysfunctions, limited mobility in daily life, and falls, leading to a reduced quality of life, and higher disability rates, ultimately an increased risk of mortality. [9, 10]

Between 2017 and 2021, seven meta-analyses assessing the prevalence of physical frailty in patients with CKD or the impact of frailty on survival were published. [11–17] However, meta-analyses on this topic have certain shortcomings, such as the inclusion of studies with overlapping populations (e.g., ACTIVE/ADIPOSE dialysis cohort [18], and CanFIT cohort [19]), missing some important relevant studies, and limited and/or lacking meta-regression analyses exploring sources of heterogeneity limit interpretation and conclusions.

Therefore, we conducted an updated, more comprehensive systematic review and meta-analysis with a larger sample size, and fewer overlapping cohorts, and its impact on survival. The primary goal of this study is to estimate the risk of mortality in patients with CKD who were affected by physical frailty. Our secondary objective is to assess the prevalence of physical frailty in patients with CKD, and to perform a detailed subgroup analysis to determine the distribution of frailty and the association of age and body mass index (BMI) with the risk of frailty-related mortality.

## Methods

This study followed the newest Preferred Reporting Items for Systemic Reviews and Meta-Analyses (PRISMA 2020) [20] (Table S1) and the Meta-analyses of Observational Studies in Epidemiology (MOOSE) guidelines. [21] The systematic review protocol has been registered on the International Prospective Register of Systematic Reviews (PROSPERO) with a registration number CRD42022320312. The current study methodology is similar to the previously described protocol (https://www.crd.york.ac.uk/PROSPERO/) with a few modifications (Table S2).

### Patient and public involvement

Patients and/or the public were not involved in the design, or conduct, or reporting, or dissemination plans of this research.

### Search strategy

We searched PubMed, Embase, Web of Science, and two clinical trial registries-ClinicalTrials.gov and Cochrane Central Register of Controlled Trials (CENTRAL), from inception to May 8, 2022. The databases were searched independently by two authors (FZ and WH). We adopt a comprehensive retrieval strategy that combines medical subject heading (MeSH) terms, title/abstract, and synonyms to retrieve eligible studies fully. The search strategies for databases are available in Table S3. Moreover, we also reviewed conference abstracts from the International Society of Nephrology (https://www.theisn.org/), European Renal Association (https://www.era-online.org/en/), and American Society of Nephrology (https://www.asn-online.org/) for the past five years to look for other potential studies. Finally, we manually investigated a relevant reference of systematic reviews to search for additional potential studies. [11–17] Any disagreements were resolved by a third reviewer’s opinion (LYH).

### Eligibility criteria

The inclusion criteria for studies were as follow: (1) adults (aged 18 years or older) confirmed CKD, including predialysis (i.e., stage 1-5 non-dialysis), peritoneal dialysis, hemodialysis, kidney transplant recipients; (2) reported prevalence of physical frailty according to a validated tool (e.g., Fried frailty phenotype; Frailty Index; Clinical Frailty Scale), or sufficient data to calculate it; (3) study reported data on physical frailty and mortality outcomes; (4) study design was limited to observational studies, including cohort study, cross-sectional study, case-control study, and longitudinal studies; (5) articles published in English.

Studies were excluded if any of the following exclusion criteria were met: (1) letter/comment/editorial, conference abstracts, and case reports; (2) studies of self-reported CKD; (3) reported other subtypes of frailty, like cognitive, psychosocial, or nutritional frailty. [22] For studies with overlapping cohorts or populations, we selected studies with more recent data, larger sample sizes, and/or richer information for inclusion. Disagreements were resolved by a third reviewer if necessary (LYH).

### Methodological quality assessment

Risk of bias of included studies was assessed using the Newcastle-Ottawa Scale (NOS). [23] The scale assesses the risk of bias in three domains: selection, comparability, and exposure/outcome. In the selection and outcome sections, three of the nine items-“demonstration that outcome of interest was not present at start of study,“ “was follow-up long enough for outcomes to occur?“ and “adequacy of follow-up of cohorts-were removed since they do not apply to cross-sectional studies. [24]

### Data extraction

Two independent authors (FZ and HW) extracted the data using an agreed form. We collected the following information from the included studies: (1) first authors; (2) publication year; (3) geographical location and country; (4) total sample size; (5) mean age and body mass index (BMI); (6) CKD stage; (7) the number of patients with physical frailty (where available, we also collected data on prefrail); (8) assessment tool used to define the presence of physical frailty; (9) the number of deaths; (10) follow-up time; 11) hazard ratio (HR) and 95% confidence interval (95% CI) of patients with frailty CKD corresponding to non-frail patients.

Where more than one study reported data from the same sample and outcome, only one study was selected based on a larger sample size or better suitability of data. When a study reported multiple instruments to assess physical frailty, we used only the results evaluated by the most used scales for inclusion in the primary meta-analysis to avoid the problem of non-independent data. If a study reported the prevalence of physical frailty at different stages of CKD, it was treated as two records included in the meta-analysis separately.

Where a study used hierarchical categorization, for example, mild, moderate, and severe frailty, for the primary analysis, we classified those in the moderate and severe category as frailty and mild as prefrail. If a study appeared potentially eligible but did not report the required data, we contacted the authors by email to obtain additional information and maximize existing studies’ availability. Studies were excluded when they did not report data available for extraction, and the authors could not be contacted. If a study reported longitudinal data on the prevalence of physical frailty in patients with CKD, only baseline data were extracted. Any disagreement about data extraction was resolved by consulting a third author (LYH).

### Data synthesis

We performed a meta-analysis using *meta* [25] and the *metafor* package [26] in R software (version 4.2.0) to obtain weighted pooled estimates of physical frailty and prefrail for all studies. The prevalence was transformed using the Freeman-Tukey double arcsine transform to approximate a normal distribution and stabilize the variance. [27] Since study designs and assessment tools vary, we pooled data using a random-effects model based on the DerSimonian-Laird inverse variance method to provide conservative estimates of physical frailty prevalence and then reverse transformed the estimates and 95% CIs and expressed them as percentages to facilitate interpretation. [28] In addition, prediction intervals were generated to provide a range of predictions of true prevalence in any individual study. [29] Heterogeneity was quantified using the τ^2^ and *I*^2^ statistics, and *I*^2^ > 50% was defined as significant heterogeneity. [30]

We performed pre-planned subgroup analyses according to the CKD stages, different assessment tools, geographical location (i.e., the corresponding continent), and study design. Possible publication bias was tested by examining the funnel plot and an Egger regression. [31, 32]

To explain the significant heterogeneity observed, meta-regression analyses were conducted using the R *metafor* package to assess the effect of the following moderator variables on the heterogeneity of the prevalence of physical frailty: mean age, the proportion of males (percentage), mean BMI, geographical location, CKD stage, and assessment tool. Moreover, using a bubble plot, we visualize the relationship between mean age, BMI, and prevalence of physical frailty and the association between frailty and mortality risk.

We performed three sensitivity analyses: (1) to exclude studies with a sample size of fewer than 100 participants to verify the possibility that the results overestimated the severity of the problem due to the small sample size; (2) considering the recent debate on the meta-analysis of proportions, [33, 34] we used generalized linear mixed models (i.e., random intercept logistic regression models) to pool the prevalence of physical frailty in CKD patients; [35] (3) using a “leave-one-out” approach, in which we removed all studies one by one to analyze their effects on the primary analysis. The grading recommendations assessment development and evaluation (GRADE) approach was used to assess the evidence on prognosis. [36]

## Results

**Fig. 1:**
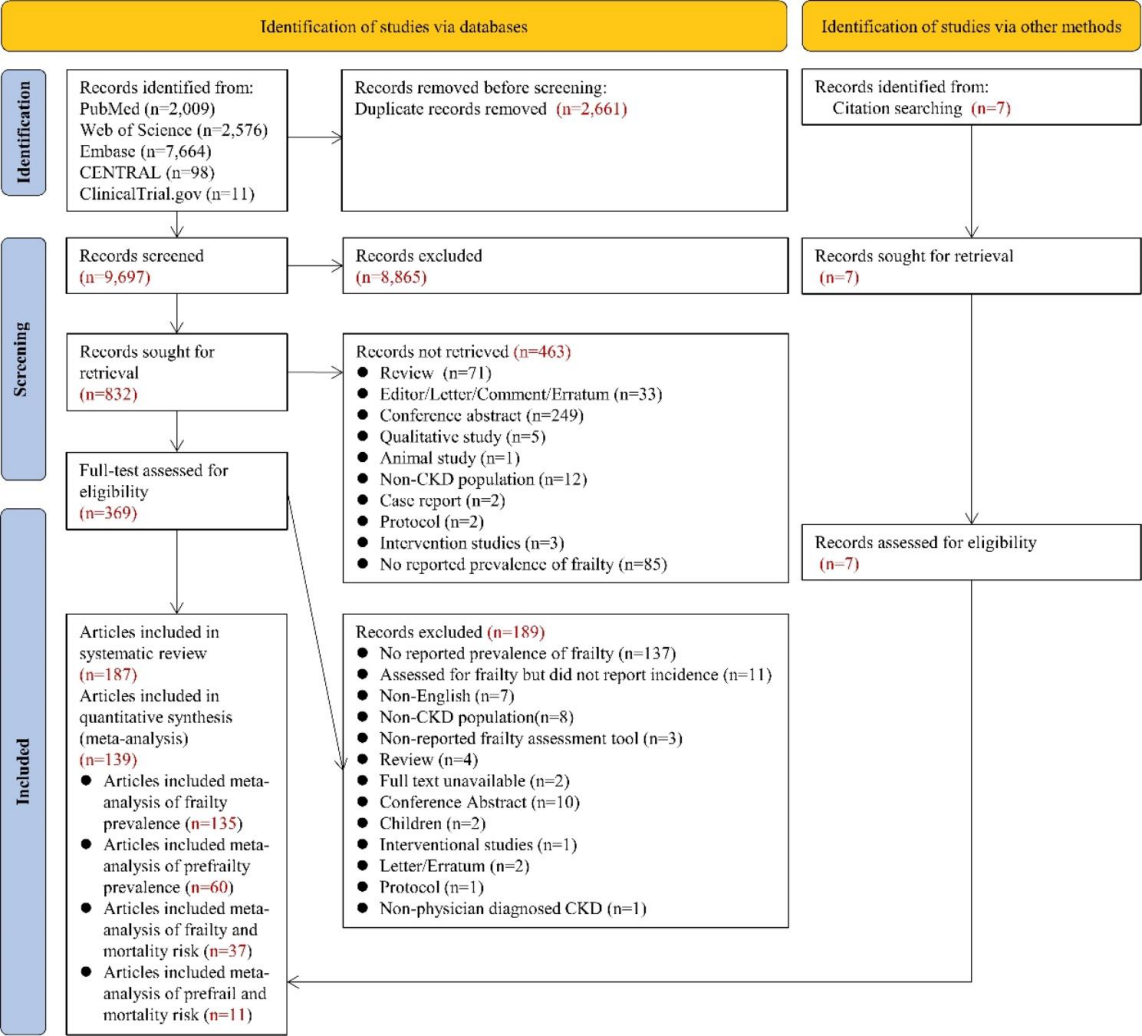
The PRISMA flowchart of studies included in the meta-analysis. Legend: CENTRAL = Cochrane Central Register of Controlled Trials; CKD = Chronic Kidney Disease

After removing duplicate articles from different databases, a total of 9,697 articles were included. These articles were first screened by title and abstract, and eventually, 369 articles and an additional seven articles were subjected to full-text review. A total of 187 articles were included after a rigorous screening process (The reasons for exclusion are listed in Table S4). Notably, some populations of included studies were duplicated (full details are listed in Table S5). One hundred and thirty-nine articles with 144 studies were ultimately included in the meta-analysis. The PRISMA flowchart is shown in Fig. 1.

### The characteristic of included studies

Table S6 summarizes the characteristics of 139 articles included in this systematic review and meta-analysis. There were 44 articles from Asia, 54 from the Americas (South and North America), 38 from Europe, and only three from Oceania. The included population was predominantly dialysis-dependent, with sample sizes ranging from 22 to 1,424,026 of the included studies. The mean age of the patients ranged from 31 to 83.7 years, and the mean BMI from 20.7 to 31.7 kg/m^2^. The scores for all studies are shown in Table S7.

### Prevalence of physical frailty in CKD patients

The results of a meta-analysis based on 135 articles (142 studies in total) with a total of 1,672,650 patients showed an overall prevalence of physical frailty in CKD patients of 34.5% (95% CI 31.0 to 38.1%), with significant heterogeneity between studies as expected (Figure S1). The effect of individual studies on the combined estimates is very small by the leave-one-out analysis (Figure S2). The prevalence of physical frailty combined using the generalized linear mixed model was 33.0% (95% CI 29.2 to 36.59%) (Figure S3), similar to the Freeman-Tukey double arcsine transform. After excluding studies with sample sizes below 100, the pooled result was 33.6% (95% CI 29.7 to 37.7%) (Figure S4).

According to the different scales for judging pre-frailty, 60 articles (61 studies in total) reported data on prefrail in patients with CKD, with a combined prevalence of 39.4% (95% CI 35.3 to43.6%) and high heterogeneity between studies (Figure S5). Leave-one-out analysis showed that individual studies had minimal effect on the pool estimates (Figure S6). The prevalence of prefrail using the generalized linear mixed model was 38.8% (95% CI 34.7 to 43.1%), slightly lower than the Freeman-Tukey double arcsine transform (Figure S7).

### Subgroup analysis for the prevalence of physical frailty in CKD patients

**Fig. 2:**
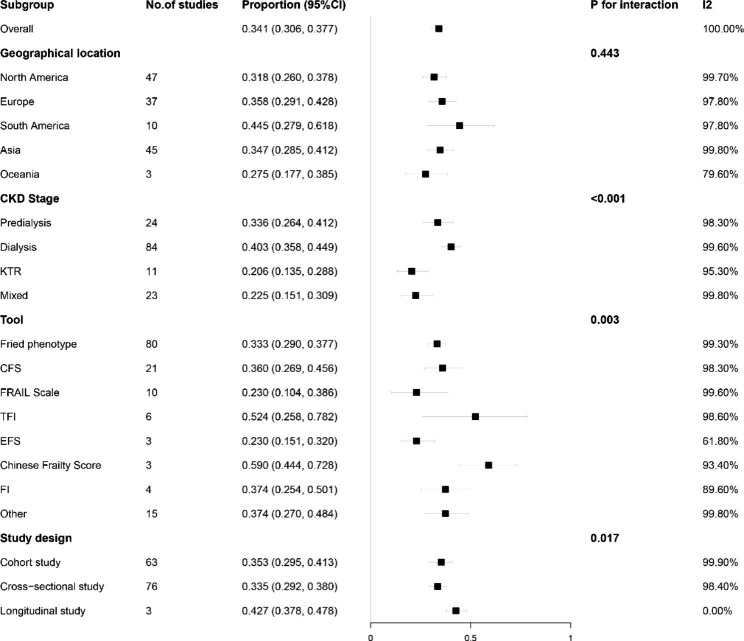
Subgroup analyses for the prevalence of physical frailty. Legend: KTR = Kidney Transplantation Recipient; CFS = Clinical Frailty Scale; TFI = Tilburg Frailty Index; EFS = Edmonton Frail Scale; FI = Frailty Index; CI = Confidence Interval

Figure 2 reports the results of subgroup analyses of physical frailty prevalence. Significant interactions were found based on the assessment tool, study design, and disease stage. A higher prevalence of physical frailty was found in the South American population and studies using the Chinese Frailty Score and Tilburg Frailty Indicator, while the lowest prevalence was found in studies using the Edmonton Frail Scale and FRAIL scales. In cross-sectional studies, lower proportions of CKD patients were frail. Regarding disease stage, kidney transplant recipients had the lowest prevalence of frailty. The heterogeneity found in most subgroup analyses was high. Based on subgroup analysis, Figure S8 shows the prevalence of frailty in 19 countries.

### Meta-regression for the prevalence of physical frailty in CKD patients

**Fig. 3:**
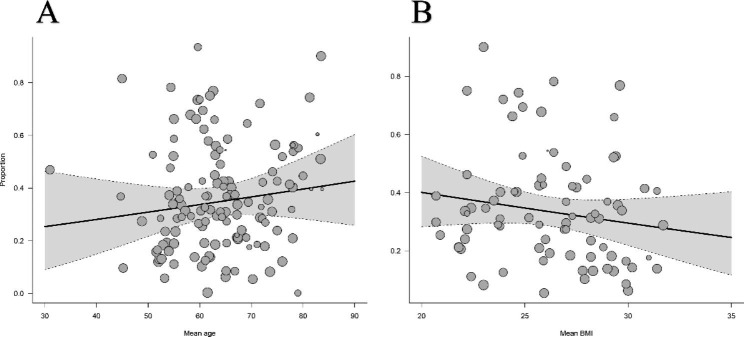
Univariable meta-regression for the prevalence of physical frailty according to study-level characteristics. Legend: A = mean age; B = mean body mass index

To explore potential heterogeneity sources in frailty prevalence, we performed univariate meta-regression analyses based on several study-level characteristics. The results identified disease stage and assessment tools as potential factors that could cause high heterogeneity (Table S8). Specifically, the prevalence of frailty was higher in dialysis-dependent CKD patients and lower in kidney transplant recipients compared to predialysis, consistent with the results of the subgroup analysis. Figure 3 shows the relationship between mean age, BMI, and prevalence of frailty.

### Association between physical frailty and mortality risk in CKD patients

From the 37 articles (38 studies in total) that provided analyzable data, physical frailty was associated with increased mortality risk in patients with CKD. The pooled adjusted HR was 1.941 (95% CI 1.586 to 2.375), but heterogeneity was high (Figure S9).

Eleven articles (12 studies in total) provided prefrail-related data. The pooled adjusted HR was 1.345 (95% CI 1.231 to 1.469), with moderate heterogeneity between studies (Figure S10). In addition, four studies reported data on continuous variables, i.e., each 1-unit increase in frailty score was associated with a 29.1% (95% CI 1.191 to 1.400) increased mortality risk (Figure S11).

Sensitivity analyses based on leave-one-out revealed no changes in the significance or direction of effects for these results (Figure S12-S13). The evidence level for these results was very low according to GRADE (Table S9).

### Meta-regression for physical frailty and mortality risk in CKD patients

Meta-regression investigating potential sources of heterogeneity demonstrated that mean age, BMI, percentage of males, and CKD stage were not significantly associated with physical frailty and mortality risk, but geographical location explained between-study heterogeneity (Table S10). Furthermore, we visualized the association of mean age and BMI with physical frailty and mortality risk (Figure S14).

### Publication bias

Funnel plots for all meta-analysis outcomes are shown in Figure S15 and were tested by Egger linear regression.

## Discussion

### Summary of main results

In this systematic review and meta-analysis of 1,672,650 CKD patients, approximately one-third (95% CI 31.0–38.1%) were physical frail, with a prediction interval pointing to a prevalence range of 3.2%–76.9%, which was a higher in older individuals and lower in those with higher BMI. Pre-frailty is defined as the transitional stage of transition from a normal clinical state to frailty. [5] The pooled results demonstrated a prevalence of pre-frailty in CKD patients of approximately 39.4% (95% CI 35.3 to 43.6%), which is slightly higher than that of frailty.

On the other hand, with significant heterogeneity, the risk of mortality in patients with frailty CKD was nearly two times higher than in patients without frailty (HR: 1.941; 95% CI 1.586 to 2.375), and the results of subgroup analysis at different stages of CKD supported this association. Further analysis revealed a positive association between physical frailty and mortality risk in those with older age and higher BMI.

### Discussion of main findings

In the past 20 years, frailty has been introduced in geriatrics as the aging population has rapidly expanded, highlighting the significant impact on patients and health services, clinical care, and chronic non-communicable diseases. [5, 6] Studies of patients with early-stage CKD and ESKD treated with long-term dialysis have reported a prevalence of physical frailty ranging from 7% in CKD stages 1–4 to 73% in hemodialysis patients, [11] and the prediction interval results of this meta-analysis support this wider result, depending on the frailty assessment tool, the participants, [10] and different glomerular filtration rate calculation formulas. [37]

The view in recent years has been that the assessment of frailty provides additional insight into the various phenomena that affect the physiologic status of patients with chronic diseases, including CKD. [38, 39] Although there is a consensus on the theoretical concept of frailty, many tools have been used to assess it. [40] Among these, the frailty phenotype, which assesses remaining physiologic reserve based on the phenotypic expression of different signs and symptoms, is most used to screen individuals for frailty, [5] whereas the frailty index provides an overall assessment of health deficits. [41]

After conducting a more comprehensive search and excluding overlapping populations, our current study provides a relatively reliable estimate of the prevalence of physical frailty in patients with CKD, documenting that approximately 30 of every 100 patients with CKD are frail and 39.4% are prefrail. This result suggests that up to 73.5% of CKD patients have some degree of physical frailty, compared to 63% in the general population. [42] It is important to note that the prevalence of physical frailty varies widely across CKD stages and should be more targeted for screening and intervention. [43] The highest risk is in the dialysis population, while the lowest is in kidney transplant recipients, with a prevalence of physical frailty of 40.3% and 20.6%. It is well known that CKD is a dynamic process. [44] In the early stages of CKD, frailty is triggered by increased catabolism due to anemia, insulin resistance, and vitamin D deficiency and progresses with loss of renal function. [45] In dialysis-dependent CKD patients, this frailty cycle is further exacerbated by metabolic acidosis, inflammation, malnutrition, and dialysis. [46] However, frailty can improve within a few months after kidney transplantation. [47] Nevertheless, it has been suggested that physical frailty may or may not be reversible and that patients may develop severe after transplantation, thus necessitating concern for post-transplant frailty. [48]

On a national level, some countries reported sparse data, with India reporting a relatively higher incidence of frailty, as shown in Figure S8. The population of India is reported to be undergoing a marked shift and is now aging at an accelerated rate. [49] It is well known that advanced age is closely associated with frailty development. [4] As a comparison, western countries such as Spain, Germany, and Australia have lower rates of frailty in CKD patients, which may be attributed to two factors: (1) outcome bias caused by a smaller included population; and (2) national emphasis on CKD management, for example, the United Stated government released the “Advancing American Kidney Health” initiative in 2019, which aims to improve the lives of Americans suffering from CKD. [50]

In the regression analysis, we observed an interesting phenomenon. The reality that the prevalence of physical frailty increases with age is unquestionable; after all, frailty is an age-related clinical condition. [51] However, the relationship between frailty and BMI is negative, although not statistically significant. According to the explanation of Lowrie et al., [52] during inflammatory states or malnutrition, body protein stores are diverted to defend against inflammation and repair damage; therefore, the increased body weight of overweight CKD patients protects against inflammation and infection, which are vital factors that exacerbate frailty development. [53] This theory may explain the lower prevalence of physical frailty in CKD patients with higher BMI.

The pooled adjusted HR associated with frailty for categorical and continuous indicators ranged from 1.291 to 1.941. In this case, the pooled adjusted HR was lower in kidney transplant recipients at 1.450 (95% CI 1.163 to 1.806) and higher in dialysis patients at 1.936 (95% CI 1.626 to 2.306). For dialysis patients, factors such as reduced physical activity and inadequate intake contribute to the high prevalence of frailty. [18, 54] In turn, frailty can cause decreased immune function and frequent infections, while inflammatory mediators released by infections cause increased protein metabolism, decreased muscle and fat volume, and worsened frailty, [55] which aggravates the development and progression of atherosclerosis through inflammatory mechanisms. [56] The three form a vicious cycle that increases the incidence of cardiovascular disease and mortality in dialysis patients. However, frailty-induced mortality is higher across the CKD spectrum, which emphasizes the need for screening and intervention for frailty in patients with all stages of CKD to prevent premature death in frail patients. This finding also highlights the need to incorporate physical frailty scores into CKD prognostic models, particularly in the dialysis population.

In patients with CKD, the “obesity paradox” has been reported, implying that higher BMI may be associated with better survival. [57] However, our meta-regression found that, although no statistical significance was observed, patients with higher BMI had a higher risk of frailty-induced death. This result is contrary to the results of Chang et al. [58]. The reason for this contradiction with the “obesity paradox” may be that defining obesity solely in terms of BMI does not quantify the body fat ratio and distribution. [59]

An unavoidable and challenging issue is the inclusion of studies using a variety of frailty assessment scales. Anderson et al. reported that the major frailty scales (Frailty Phenotype, Frailty Index, Edmonton Frailty Scale, and Clinical Frailty Scale) were similar in assessing the prevalence of frailty in dialysis-dependent CKD patients. [60] However, the agreement in frailty status between instruments was weak at best, which may explain the reason for the high heterogeneity of this study. Nevertheless, Imamura K et al. reported that the Fried Frailty Phenotype, Study of Osteoporotic Fractures Index, Short Physical Performance Battery, and Clinical Frailty Scale were similar about predicting clinical events in outpatient hemodialysis patients; also, Imamura K et al. suggested that caution should be considered when using questionnaire-based frailty assessments. [61] These findings suggest that it may be feasible and informative to pool studies for meta-analysis for the specific purpose of this study, based on the support of available evidence in the literature.

### Clinical and public health implications

The results of our study have important clinical and public health implications. From a clinical perspective, assessment of frailty and consequent individualized care can improve physical condition before dialysis and awaiting kidney transplantation by assigning intervention strategies appropriate to the individual. It has been argued that the degree of frailty in ESKD is one of the critical decision factors for receiving renal replacement therapy. [62, 63] Nephrologists are less likely to recommend dialysis treatment for patients with established frailty. [64] Furthermore, Haugen et al. reported that among kidney transplant candidates, the enrollment rate of frail patients was 38% lower (HR: 0.62; 95% CI 0.56 to 0.69) compared to non-frail patients. [65] Various interventions have been proposed to influence the frailty state. Some interventions such as prehabilitation [66], aerobic exercise and resistance training, and strict blood pressure/glycemic control have an established role in managing frailty in CKD. [43] For the reversal of frailty in dialysis patients, the current study proposes a strategy of exercise training combined with oral nutritional supplementation, which has gained some benefits. [67, 68]

The developmental benefits of reversing frailty are not limited to the patient level but may also positively impact public health. Based on our findings on the positive correlation between the prevalence of physical frailty and age in CKD and the progression of frailty with declining renal function, early detection, and intervention in predialysis stages and the provision of timely, accessible, and adequate affordable support for CKD patients through effective referral mechanisms to prolong the functional decline trajectory of older patients as much as possible, which is the concept of “healthy aging” advocated by the World Health Organization. [69]

### Strength and limitation

This is the largest meta-analysis to date focusing on studies of the prevalence of frailty in CKD patients and its impact on the mortality risk in this population. We acknowledge the following limitations. First, statistical heterogeneity between studies remained high even after subgroup analysis of disease stage, geographic location, study design, and assessment tools. This suggests differences in the demographic characteristics of the included study populations that were not explained in this study (e.g., comorbidity, ethnicity). It may have partially contributed to the differences in reported clinical outcomes between studies. Nonetheless, high heterogeneity is a common problem in epidemiological meta-analysis and has been gradually accepted. [70] Second, small sample studies may compromise the statistical efficacy of the study. Therefore, we performed the additional manipulation of excluding studies with sample sizes below 100 to observe changes in effect sizes. The results were similar to those of the primary analysis. Third, although frailty is a dynamic process, variables including changes in frailty over time after diagnosis of CKD, dialysis initiation, and kidney transplantation were unavailable due to the nature of the meta-analysis and require further investigation. Fourth, despite our rigorous review, there may be partial overlap in the included studies. Fifth, different included studies adjusted for different confounders in the multivariate Cox regression model, which may have contributed to the high heterogeneity of the pooled adjusted HR. A better approach would be to use individual patient meta-analysis to further assess the relationship between frailty and survival after adjusting for key confounding variables. Sixth, the funnel plot asymmetry and the significance of the Egger regression test suggest a possible publication bias in this study. Seventh, because the included studies were designed as observational studies, the level of evidence for frailty and mortality risk in CKD was rated as very low in terms of GRADE. Given these limitations, the results of this systematic review and meta-analysis should be interpreted cautiously.

## Conclusion

In conclusion, this systematic review and meta-analysis suggest that frailty affects approximately one-third of patients with CKD. The current study also showed that frailty was associated with increased mortality risk among patients at different stages of CKD. Considering this, (1) frailty should be an essential consideration in decision-making, prognosis, and advance care planning for patients with CKD; (2) all patients with CKD, regardless of their degree of renal dysfunction, should be regularly assessed for frailty status with a standardized tool; and (3) more studies are needed to incorporate frailty into the prognostic scores of patients with CKD.

### Electronic supplementary material

Below is the link to the electronic supplementary material.

Supplementary Material 1

## Data Availability

This meta-analysis was a secondary analysis of raw data from published original articles. All the data used for the study are included in the manuscript and supplementary material.

## Declarations

### Patient and public involvement

Patients and/or the public were not involved in the design, or conduct, or reporting, or dissemination plans of this research.

## References

[1] Webster AC, Nagler EV, Morton RL, Masson P (2017). Chronic kidney disease. Lancet.

[2] Romagnani P, Remuzzi G, Glassock R, Levin A, Jager KJ, Tonelli M, Massy Z, Wanner C, Anders HJ (2017). Chronic kidney disease. Nat Rev Dis Primers.

[3] Collaboration GBDCKD (2020). Global, regional, and national burden of chronic kidney disease, 1990–2017: a systematic analysis for the global burden of Disease Study 2017. Lancet.

[4] Clegg A, Young J, Iliffe S, Rikkert MO, Rockwood K (2013). Frailty in elderly people. Lancet.

[5] Fried LP, Tangen CM, Walston J, Newman AB, Hirsch C, Gottdiener J, Seeman T, Tracy R, Kop WJ, Burke G (2001). Frailty in older adults: evidence for a phenotype. J Gerontol A Biol Sci Med Sci.

[6] Hoogendijk EO, Afilalo J, Ensrud KE, Kowal P, Onder G, Fried LP (2019). Frailty: implications for clinical practice and public health. Lancet.

[7] Musso CG, Jauregui JR, Macías Núñez JF (2015). Frailty phenotype and chronic kidney disease: a review of the literature. Int Urol Nephrol.

[8] Portilla Franco ME, Tornero Molina F, Gil Gregorio P (2016). Frailty in elderly people with chronic kidney disease. Nefrologia.

[9] Nixon AC, Bampouras TM, Pendleton N, Woywodt A, Mitra S, Dhaygude A (2018). Frailty and chronic kidney disease: current evidence and continuing uncertainties. Clin Kidney J.

[10] Wong L, Duque G, McMahon LP (2021). Sarcopenia and Frailty: Challenges in Mainstream Nephrology Practice. Kidney Int Rep.

[11] Chowdhury R, Peel NM, Krosch M, Hubbard RE (2017). Frailty and chronic kidney disease: a systematic review. Arch Gerontol Geriatr.

[12] Kojima G (2017). Prevalence of frailty in end-stage renal disease: a systematic review and meta-analysis. Int Urol Nephrol.

[13] Lee HJ, Son YJ. Prevalence and Associated factors of Frailty and Mortality in patients with end-stage renal disease undergoing hemodialysis: a systematic review and Meta-analysis. Int J Environ Res Public Health 2021, 18(7). 10.3390/ijerph18073471 PMC8037521 33801577

[14] Mei F, Gao Q, Chen F, Zhao L, Shang Y, Hu K, Zhang W, Zhao B, Ma B (2021). Frailty as a predictor of negative health outcomes in chronic kidney disease: a systematic review and Meta-analysis. J Am Med Dir Assoc.

[15] Quint EE, Zogaj D, Banning LBD, Benjamens S, Annema C, Bakker SJL, Nieuwenhuijs-Moeke GJ, Segev DL, McAdams-DeMarco MA, Pol RA (2021). Frailty and kidney transplantation: a systematic review and Meta-analysis. Transpl Direct.

[16] Zhang Q, Ma Y, Lin F, Zhao J, Xiong J (2020). Frailty and mortality among patients with chronic kidney disease and end-stage renal disease: a systematic review and meta-analysis. Int Urol Nephrol.

[17] Zhao Y, Liu Q, Ji J (2020). The prevalence of frailty in patients on hemodialysis: a systematic review and meta-analysis. Int Urol Nephrol.

[18] Johansen KL, Dalrymple LS, Delgado C, Chertow GM, Segal MR, Chiang J, Grimes B, Kaysen GA (2017). Factors Associated with Frailty and its trajectory among patients on Hemodialysis. Clin J Am Soc Nephrol.

[19] Brar RS, Whitlock RH, Komenda P, Rigatto C, Prasad B, Bohm C, Tangri N (2021). Provider perception of Frailty is Associated with Dialysis decision making in patients with Advanced CKD. Clin J Am Soc Nephrol.

[20] Page MJ, McKenzie JE, Bossuyt PM, Boutron I, Hoffmann TC, Mulrow CD, Shamseer L, Tetzlaff JM, Akl EA, Brennan SE (2021). The PRISMA 2020 statement: an updated guideline for reporting systematic reviews. BMJ.

[21] Stroup DF, Berlin JA, Morton SC, Olkin I, Williamson GD, Rennie D, Moher D, Becker BJ, Sipe TA, Thacker SB (2000). Meta-analysis of observational studies in epidemiology: a proposal for reporting. Meta-analysis of Observational Studies in Epidemiology (MOOSE) group. JAMA.

[22] Ijaz N, Buta B, Xue QL, Mohess DT, Bushan A, Tran H, Batchelor W, deFilippi CR, Walston JD, Bandeen-Roche K (2022). Interventions for Frailty among older adults with Cardiovascular Disease: JACC State-of-the-art review. J Am Coll Cardiol.

[23] The Newcastle-Ottawa. Scale (NOS) for assessing the quality of nonrandomised studies in meta-analyses [https://www.ohri.ca//programs/clinical_epidemiology/oxford.asp].

[24] Breton MC, Guénette L, Amiche MA, Kayibanda JF, Grégoire JP, Moisan J (2013). Burden of diabetes on the ability to work: a systematic review. Diabetes Care.

[25] Schwarzer G. meta: An R Package for Meta-Analysis. 2007, 7:40–45.

[26] Viechtbauer W. Conducting Meta-analyses in R with the metafor Package. J Stat Softw 2010, 36.

[27] Barendregt JJ, Doi SA, Lee YY, Norman RE, Vos T (2013). Meta-analysis of prevalence. J Epidemiol Community Health.

[28] Munn Z, Moola S, Lisy K, Riitano D, Tufanaru C (2015). Methodological guidance for systematic reviews of observational epidemiological studies reporting prevalence and cumulative incidence data. Int J Evid Based Healthc.

[29] Riley RD, Higgins JP, Deeks JJ (2011). Interpretation of random effects meta-analyses. BMJ.

[30] Higgins JP, Thompson SG, Deeks JJ, Altman DG (2003). Measuring inconsistency in meta-analyses. BMJ.

[31] Egger M, Davey Smith G, Schneider M, Minder C (1997). Bias in meta-analysis detected by a simple, graphical test. BMJ.

[32] Sterne J, Harbord R (2004). Funnel plots in Meta-analysis. Stata J.

[33] Doi SA, Xu C (2021). The Freeman-Tukey double arcsine transformation for the meta-analysis of proportions: recent criticisms were seriously misleading. J Evid Based Med.

[34] Schwarzer G, Chemaitelly H, Abu-Raddad LJ, Rücker G (2019). Seriously misleading results using inverse of Freeman-Tukey double arcsine transformation in meta-analysis of single proportions. Res Synth Methods.

[35] Lin L, Chu H (2020). Meta-analysis of Proportions using generalized Linear mixed models. Epidemiology.

[36] Iorio A, Spencer FA, Falavigna M, Alba C, Lang E, Burnand B, McGinn T, Hayden J, Williams K, Shea B (2015). Use of GRADE for assessment of evidence about prognosis: rating confidence in estimates of event rates in broad categories of patients. BMJ.

[37] Dalrymple LS, Katz R, Rifkin DE, Siscovick D, Newman AB, Fried LF, Sarnak MJ, Odden MC, Shlipak MG (2013). Kidney function and prevalent and incident frailty. Clin J Am Soc Nephrol.

[38] Cesari M, Prince M, Thiyagarajan JA, De Carvalho IA, Bernabei R, Chan P, Gutierrez-Robledo LM, Michel JP, Morley JE, Ong P (2016). Frailty: an emerging Public Health Priority. J Am Med Dir Assoc.

[39] Otobe Y, Rhee CM, Nguyen M, Kalantar-Zadeh K, Kopple JD (2022). Current status of the assessment of sarcopenia, frailty, physical performance and functional status in chronic kidney disease patients. Curr Opin Nephrol Hypertens.

[40] Morley JE, Vellas B, van Kan GA, Anker SD, Bauer JM, Bernabei R, Cesari M, Chumlea WC, Doehner W, Evans J (2013). Frailty consensus: a call to action. J Am Med Dir Assoc.

[41] Mitnitski AB, Mogilner AJ, Rockwood K (2001). Accumulation of deficits as a proxy measure of aging. ScientificWorldJournal.

[42] O’Caoimh R, Sezgin D, O’Donovan MR, Molloy DW, Clegg A, Rockwood K, Liew A (2021). Prevalence of frailty in 62 countries across the world: a systematic review and meta-analysis of population-level studies. Age Ageing.

[43] Kennard A, Glasgow N, Rainsford S, Talaulikar G (2023). Frailty in chronic kidney disease: challenges in nephrology practice. A review of current literature. Intern Med J.

[44] Eckardt KU, Coresh J, Devuyst O, Johnson RJ, Köttgen A, Levey AS, Levin A (2013). Evolving importance of kidney disease: from subspecialty to global health burden. Lancet.

[45] Guerville F, de Souto Barreto P, Taton B, Bourdel-Marchasson I, Rolland Y, Vellas B (2019). Estimated glomerular filtration rate decline and Incident Frailty in older adults. Clin J Am Soc Nephrol.

[46] Lorenz EC, Kennedy CC, Rule AD, LeBrasseur NK, Kirkland JL, Hickson LJ (2021). Frailty in CKD and Transplantation. Kidney Int Rep.

[47] McAdams-DeMarco MA, Isaacs K, Darko L, Salter ML, Gupta N, King EA, Walston J, Segev DL (2015). Changes in Frailty after kidney transplantation. J Am Geriatr Soc.

[48] Vleut R, Abramowicz D, Hellemans R (2020). Frailty: a new comorbidity in kidney transplant candidates?. Nephrol Dial Transplant.

[49] Ravindranath V, Sundarakumar JS (2021). Changing demography and the challenge of dementia in India. Nat Rev Neurol.

[50] HHS Launches President Trump’s. ‘Advancing American Kidney Health’ Initiative [https://public3.pagefreezer.com/browse/HHS.gov/31-12-2020T08:51/https://www.hhs.gov/about/news/2019/07/10/hhs-launches-president-trump-advancing-american-kidney-health-initiative.html].

[51] Dent E, Martin FC, Bergman H, Woo J, Romero-Ortuno R, Walston JD (2019). Management of frailty: opportunities, challenges, and future directions. Lancet.

[52] Lowrie EG (1998). Acute-phase inflammatory process contributes to malnutrition, anemia, and possibly other abnormalities in dialysis patients. Am J Kidney Dis.

[53] Angulo J, El Assar M, Álvarez-Bustos A, Rodríguez-Mañas L (2020). Physical activity and exercise: strategies to manage frailty. Redox Biol.

[54] Chan GC, Ng JK, Chow KM, Kwong VW, Pang WF, Cheng PM, Law MC, Leung CB, Li PK, Szeto CC (2021). Progression in physical Frailty in Peritoneal Dialysis Patients. Kidney Blood Press Res.

[55] Chalupsky M, Goodson DA, Gamboa JL, Roshanravan B (2021). New insights into muscle function in chronic kidney disease and metabolic acidosis. Curr Opin Nephrol Hypertens.

[56] Soysal P, Arik F, Smith L, Jackson SE, Isik AT (2020). Inflammation, Frailty and Cardiovascular Disease. Adv Exp Med Biol.

[57] Park J, Ahmadi SF, Streja E, Molnar MZ, Flegal KM, Gillen D, Kovesdy CP, Kalantar-Zadeh K (2014). Obesity paradox in end-stage kidney disease patients. Prog Cardiovasc Dis.

[58] Chang TI, Ngo V, Streja E, Chou JA, Tortorici AR, Kim TH, Kim TW, Soohoo M, Gillen D, Rhee CM (2017). Association of body weight changes with mortality in incident hemodialysis patients. Nephrol Dial Transplant.

[59] Crow RS, Lohman MC, Titus AJ, Cook SB, Bruce ML, Mackenzie TA, Bartels SJ, Batsis JA (2019). Association of obesity and Frailty in older adults: NHANES 1999–2004. J Nutr Health Aging.

[60] Anderson BM, Qasim M, Correa G, Evison F, Gallier S, Ferro CJ, Jackson TA, Sharif A (2022). Correlations, agreement and utility of frailty instruments in prevalent haemodialysis patients: baseline cohort data from the FITNESS study. Clin Kidney J.

[61] Imamura K, Yamamoto S, Suzuki Y, Yoshikoshi S, Harada M, Osada S, Kamiya K, Matsuzawa R, Matsunaga A (2023). Comparison of the association between six different frailty scales and clinical events in patients on hemodialysis. Nephrol Dial Transplant.

[62] Berger JR, Jaikaransingh V, Hedayati SS (2016). End-stage kidney disease in the Elderly: Approach to Dialysis initiation, choosing modality, and Predicting Outcomes. Adv Chronic Kidney Dis.

[63] Harhay MN, Rao MK, Woodside KJ, Johansen KL, Lentine KL, Tullius SG, Parsons RF, Alhamad T, Berger J, Cheng XS (2020). An overview of frailty in kidney transplantation: measurement, management and future considerations. Nephrol Dial Transplant.

[64] Foote C, Morton RL, Jardine M, Gallagher M, Brown M, Howard K, Cass A (2014). COnsiderations of nephrologists when SuggestIng Dialysis in Elderly patients with renal failure (CONSIDER): a discrete choice experiment. Nephrol Dial Transplant.

[65] Haugen CE, Chu NM, Ying H, Warsame F, Holscher CM, Desai NM, Jones MR, Norman SP, Brennan DC, Garonzik-Wang J (2019). Frailty and Access to kidney transplantation. Clin J Am Soc Nephrol.

[66] Kobashigawa J, Dadhania D, Bhorade S, Adey D, Berger J, Bhat G, Budev M, Duarte-Rojo A, Dunn M, Hall S (2019). Report from the American Society of transplantation on frailty in solid organ transplantation. Am J Transplant.

[67] Ikizler TA (2019). Intradialytic nutrition and exercise: convenience versus efficacy. Kidney Int.

[68] Martin-Alemañy G, Espinosa-Cuevas M, Pérez-Navarro M, Wilund KR, Miranda-Alatriste P, Cortés-Pérez M, García-Villalobos G, Gómez-Guerrero I, Cantú-Quintanilla G, Ramírez-Mendoza M (2020). Effect of oral nutritional supplementation with and without Exercise on Nutritional Status and physical function of adult hemodialysis patients: a parallel controlled clinical trial (AVANTE-HEMO study). J Ren Nutr.

[69] Beard JR, Officer A, de Carvalho IA, Sadana R, Pot AM, Michel JP, Lloyd-Sherlock P, Epping-Jordan JE, Peeters G, Mahanani WR (2016). The World report on ageing and health: a policy framework for healthy ageing. Lancet.

[70] Barberio B, Zamani M, Black CJ, Savarino EV, Ford AC (2021). Prevalence of symptoms of anxiety and depression in patients with inflammatory bowel disease: a systematic review and meta-analysis. Lancet Gastroenterol Hepatol.

