# Supplementary material for: Prevalence of physical frailty and impact on survival in patients with chronic kidney disease: a systematic review and meta-analysis
Source: BMC Nephrol. 2023 Sep 3;24:258. doi: 10.1186/s12882-023-03303-1 (PMC10476333; doi:10.1186/s12882-023-03303-1)
Supplement: Supplementary file 1 — Supplementary Material 1 [file 12882_2023_3303_MOESM1_ESM.docx]

**Prevalence of physical frailty and impact on survival in patients with chronic kidney disease: A systematic review and meta-analysis.**

**Supplementary Material**

Content

[Instructions 2](#_Toc143121995)

[Table S1 The PRISMA 2020 Checklist 3](#_Toc143121996)

[Table S2 Protocol deviations 7](#_Toc143121997)

[Table S3 Search detailed for database 8](#_Toc143121998)

[Table S4 List of studies excluded at full-text review and reasons for exclusion 12](#_Toc143121999)

[Table S5 The list of studies that were excluded from the meta-analysis due to data overlapping 30](#_Toc143122000)

[Table S6 Characteristic of included 187 articles 35](#_Toc143122001)

[Table S7 Assessment of risk of bias in the included studies using Newcastle-Ottawa Scale (NOS) 69](#_Toc143122002)

[Description of the NOS scale 75](#_Toc143122003)

[Table S8 Meta-regression for the prevalence of frailty 79](#_Toc143122004)

[Table S9 GRADE evidence profile: frailty and prefrail for mortality in patients with CKD 80](#_Toc143122005)

[Table S10 Meta-regression for the association of frailty and mortality risk 81](#_Toc143122006)

[Figure S1 The physical frailty prevalence based on Freeman-Tukey double arcsine transformation 82](#_Toc143122007)

[Figure S2 The sensitive analysis for the prevalence of physical frailty based on leave-one-out 83](#_Toc143122008)

[Figure S3 The physical frailty prevalence based on generalized linear mixed model 84](#_Toc143122009)

[Figure S4 Forest plot of physical frailty prevalence after removing sample size below 100 85](#_Toc143122010)

[Figure S5 The prefrail prevalence based on Freeman-Tukey double arcsine transformation 86](#_Toc143122011)

[Figure S6 The sensitive analysis for the prevalence of prefrail based on leave-one-out 87](#_Toc143122012)

[Figure S7 The physical frailty prevalence based on generalized linear mixed model 88](#_Toc143122013)

[Figure S8 Prevalence of frailty in CKD patients based on country distribution 89](#_Toc143122014)

[Figure S9 Association between frailty and risk of mortality in patients with CKD 90](#_Toc143122015)

[Figure S10 Association between prefrail and risk of mortality in patients with CKD 91](#_Toc143122016)

[Figure S11 Association of each 1-unit increase in frailty score with the risk of mortality in patients with CKD 92](#_Toc143122017)

[Figure S12 The sensitive analysis for the association between physical frailty and mortality risk based on leave-one-out 93](#_Toc143122018)

[Figure S13 The sensitive analysis for the association between prefrail and mortality risk based on leave-one-out 94](#_Toc143122019)

[Figure S14 Univariable meta-regression for the prevalence of physical frailty according to study-level characteristics 95](#_Toc143122020)

[Figure S15 Funnel plots corresponding to the four meta-analyses 96](#_Toc143122021)

[Reference 97](#_Toc143122022)

Instructions

1. Full-text screening of 369 articles has been uploaded as Supplementary Material, except for some conference abstracts and unavailable full-text to *txt* instead.

2. The serial numbers of the full-text *pdf* file naming correspond to the numbers marked in red in Table S3-S6.

3. When age and body mass index were described by median, quartiles or median, range, they were converted to mean ± standard deviation using the method of Luo et al. and Wan et al.

Luo, D., Wan, X., Liu, J., & Tong, T. (2018). Optimally estimating the sample mean from the sample size, median, mid-range, and/or mid-quartile range. *Statistical methods in medical research*, *27*(6), 1785–1805.

Wan, X., Wang, W., Liu, J., & Tong, T. (2014). Estimating the sample mean and standard deviation from the sample size, median, range and/or interquartile range. *BMC medical research methodology*, *14*, 135.

4. When studies reported subgroups (e.g., gender, age group), they were combined using following Equation:

Total sample: N=N_1_+N_2_

Mean: M=(N_1_M_1_+N_2_M_2_)/(N_1_+N_2_)

Standard deviation: SD=$\sqrt{\frac{{(N}_{1}-1){SD}_{1}^{2}+(N_{2}-1){SD}_{2}^{2}+\frac{N1N2}{N1+N2}(M_{1}^{2}+M_{2}^{2}-2M_{1}M_{2})}{N_{1}+N_{2}-1}}$

5. We are very sorry for the authors Mutevelić-Turković A, Pérez-Sáez MJ, Zachciał J, whose names contain symbols that are not recognized by the *R* software, and we took the liberty to change them to similar alphabets, we did not mean to offend.

Table S1 The PRISMA 2020 Checklist

| **Section and Topic** | **Item #** | **Checklist item** | **Location where item is reported** |
| --- | --- | --- | --- |
| **TITLE** | | |  |
| Title | 1 | Identify the report as a systematic review. | Page 1 |
| **ABSTRACT** | | |  |
| Abstract | 2 | See the PRISMA 2020 for Abstracts checklist. | Page 2-3 |
| **INTRODUCTION** | | |  |
| Rationale | 3 | Describe the rationale for the review in the context of existing knowledge. | Page 5 |
| Objectives | 4 | Provide an explicit statement of the objective(s) or question(s) the review addresses. | Page 5 |
| **METHODS** | | |  |
| Eligibility criteria | 5 | Specify the inclusion and exclusion criteria for the review and how studies were grouped for the syntheses. | Page 6-7 |
| Information sources | 6 | Specify all databases, registers, websites, organisations, reference lists and other sources searched or consulted to identify studies. Specify the date when each source was last searched or consulted. | Page 6 |
| Search strategy | 7 | Present the full search strategies for all databases, registers and websites, including any filters and limits used. | See Table S2 |
| Selection process | 8 | Specify the methods used to decide whether a study met the inclusion criteria of the review, including how many reviewers screened each record and each report retrieved, whether they worked independently, and if applicable, details of automation tools used in the process. | Page 6 |
| Data collection process | 9 | Specify the methods used to collect data from reports, including how many reviewers collected data from each report, whether they worked independently, any processes for obtaining or confirming data from study investigators, and if applicable, details of automation tools used in the process. | Page 7-8 |
| Data items | 10a | List and define all outcomes for which data were sought. Specify whether all results that were compatible with each outcome domain in each study were sought (e.g. for all measures, time points, analyses), and if not, the methods used to decide which results to collect. | Page 7-8 |
|  | 10b | List and define all other variables for which data were sought (e.g. participant and intervention characteristics, funding sources). Describe any assumptions made about any missing or unclear information. | Page 7-8 |
| Study risk of bias assessment | 11 | Specify the methods used to assess risk of bias in the included studies, including details of the tool(s) used, how many reviewers assessed each study and whether they worked independently, and if applicable, details of automation tools used in the process. | Page 7 |
| Effect measures | 12 | Specify for each outcome the effect measure(s) (e.g. risk ratio, mean difference) used in the synthesis or presentation of results. | Page 8 |
| Synthesis methods | 13a | Describe the processes used to decide which studies were eligible for each synthesis (e.g. tabulating the study intervention characteristics and comparing against the planned groups for each synthesis (item #5)). | Page 8 |
|  | 13b | Describe any methods required to prepare the data for presentation or synthesis, such as handling of missing summary statistics, or data conversions. | Page 8 |
|  | 13c | Describe any methods used to tabulate or visually display results of individual studies and syntheses. | Page 8 |
|  | 13d | Describe any methods used to synthesize results and provide a rationale for the choice(s). If meta-analysis was performed, describe the model(s), method(s) to identify the presence and extent of statistical heterogeneity, and software package(s) used. | Page 8-9 |
|  | 13e | Describe any methods used to explore possible causes of heterogeneity among study results (e.g. subgroup analysis, meta-regression). | Page 8-9 |
|  | 13f | Describe any sensitivity analyses conducted to assess robustness of the synthesized results. | Page 8-9 |
| Reporting bias assessment | 14 | Describe any methods used to assess risk of bias due to missing results in a synthesis (arising from reporting biases). | Page 7 |
| Certainty assessment | 15 | Describe any methods used to assess certainty (or confidence) in the body of evidence for an outcome. | Page 9 |
| **RESULTS** | | |  |
| Study selection | 16a | Describe the results of the search and selection process, from the number of records identified in the search to the number of studies included in the review, ideally using a flow diagram. | See Figure 1 |
|  | 16b | Cite studies that might appear to meet the inclusion criteria, but which were excluded, and explain why they were excluded. | Page 9 |
| Study characteristics | 17 | Cite each included study and present its characteristics. | See Table S5 |
| Risk of bias in studies | 18 | Present assessments of risk of bias for each included study. | See Table S6 |
| Results of individual studies | 19 | For all outcomes, present, for each study: (a) summary statistics for each group (where appropriate) and (b) an effect estimate and its precision (e.g. confidence/credible interval), ideally using structured tables or plots. | Page 9-10 |
| Results of syntheses | 20a | For each synthesis, briefly summarise the characteristics and risk of bias among contributing studies. | Page 10-11 |
|  | 20b | Present results of all statistical syntheses conducted. If meta-analysis was done, present for each the summary estimate and its precision (e.g. confidence/credible interval) and measures of statistical heterogeneity. If comparing groups, describe the direction of the effect. | Page 10-11 |
|  | 20c | Present results of all investigations of possible causes of heterogeneity among study results. | Page 10-11 |
|  | 20d | Present results of all sensitivity analyses conducted to assess the robustness of the synthesized results. | See Figure S3, Figure S4, and so on |
| Reporting biases | 21 | Present assessments of risk of bias due to missing results (arising from reporting biases) for each synthesis assessed. | Not applicable |
| Certainty of evidence | 22 | Present assessments of certainty (or confidence) in the body of evidence for each outcome assessed. | See Table S8 |
| **DISCUSSION** | | |  |
| Discussion | 23a | Provide a general interpretation of the results in the context of other evidence. | Page 11-12 |
|  | 23b | Discuss any limitations of the evidence included in the review. | Page 13-14 |
|  | 23c | Discuss any limitations of the review processes used. | Page 15 |
|  | 23d | Discuss implications of the results for practice, policy, and future research. | Page 24-25 |
| **OTHER INFORMATION** | | |  |
| Registration and protocol | 24a | Provide registration information for the review, including register name and registration number, or state that the review was not registered. | Page 6 |
|  | 24b | Indicate where the review protocol can be accessed, or state that a protocol was not prepared. | Page 6 |
|  | 24c | Describe and explain any amendments to information provided at registration or in the protocol. | Not applicable |
| Support | 25 | Describe sources of financial or non-financial support for the review, and the role of the funders or sponsors in the review. | Page 16 |
| Competing interests | 26 | Declare any competing interests of review authors. | Page 16 |
| Availability of data, code and other materials | 27 | Report which of the following are publicly available and where they can be found: template data collection forms; data extracted from included studies; data used for all analyses; analytic code; any other materials used in the review. | Page 16 |

Table S2 Protocol deviations

| **Section** | **Previous protocol** | **Publication** |
| --- | --- | --- |
| Title | Frailty in patients with chronic kidney disease: a systematic review and meta-analysis | Prevalence of physical frailty and impact on survival in patients with chronic kidney disease: A systematic review and meta-analysis |
| Author | Fan Zhang, Hui Wang, Yan Bai, Ying Zhang, Liuyan Huang, Qiuzi Sun, Huachun Zhang | Fan Zhang, Hui Wang, Yan Bai, Ying Zhang, Liuyan Huang, Huachun Zhang |
| Literature search (gray literature) | None reported. | We also reviewed conference abstracts from the International Society of Nephrology (https://www.theisn.org/), European Renal Association (https://www.era-online.org/en/), and American Society of Nephrology (https://www.asn-online.org/) for the past five years to look for other potential studies. |
| Strategy for data synthesis | Data analysis was performed with Stata version 12. StataCorp. | We performed a meta-analysis using meta and the metafor package in R software (version 4.2.0). |
| Analysis of subgroups or subsets | Subgroup analysis will be used to identify the incidence of frailty in different CKD stages. | We performed a subgroup analyses according to the CKD stages, different assessment tools, geographical location (i.e., the corresponding continent), and study design. |

Table S3 Search detailed for database

| **PubMed (n=2009)** | **#** | **Search strategy** |
| --- | --- | --- |
|  | 1 | "Renal Insufficiency, Chronic"[MeSH Terms] OR "Renal Insufficiency"[MeSH Terms] |
|  | 2 | "Chronic Renal Insufficiency"[Title/Abstract] OR "Chronic Kidney Insufficiency"[Title/Abstract] OR "Chronic Kidney Disease"[Title/Abstract] or "Chronic Renal Disease"[Title/Abstract] OR "Predialysis"[Title/Abstract] OR "Pre-dialysis"[Title/Abstract] |
|  | 3 | "CKD"[Title/Abstract] OR "CKF"[Title/Abstract] OR "CRD"[Title/Abstract] OR "CRF"[Title/Abstract] |
|  | 4 | "End-Stage Kidney"[Title/Abstract] OR "End-Stage Renal"[Title/Abstract] OR "Endstage Kidney"[Title/Abstract] Or "Endstage Renal"[Title/Abstract] |
|  | 5 | "ESRD"[Title/Abstract] OR "ESRF"[Title/Abstract] OR "ESKD"[Title/Abstract] OR "ESKF"[Title/Abstract] |
|  | 6 | "Renal Replacement Therapy"[MeSH Terms] |
|  | 7 | "Dialysis"[Title/Abstract] |
|  | 8 | "Hemodialysis"[Title/Abstract] OR "Haemodialysis"[Title/Abstract] OR "Hemodiafiltration"[Title/Abstract] OR "Haemodiafiltration"[Title/Abstract] OR "HD"[Title/Abstract] OR "Hemofiltration"[Title/Abstract] OR "Haemofiltration"[Title/Abstract] |
|  | 9 | "PD"[Title/Abstract] |
|  | 10 | "Renal Transplantation"[Title/Abstract] OR "Kidney Grafting"[Title/Abstract] OR "Kidney Transplantation"[Title/Abstract] |
|  | 11 | "KTRs"[Title/Abstract] |
|  | 12 | OR/1-11 |
|  | 13 | "Frailty"[MeSH Terms] |
|  | 14 | "Frail*"[Title/Abstract] OR "Debilit*"[Title/Abstract] |
|  | 15 | OR/13-14 |
|  | 17 | AND/12,15 |
| 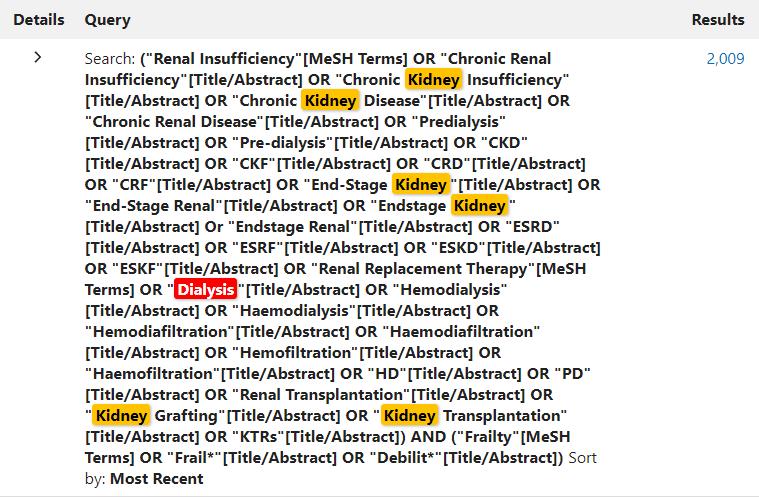 | | |
| **CENTRAL (n=98)** | 1 | MeSH descriptor: [Renal Insufficiency] explode all trees |
|  | 2 | ("chronic kidney disease" or "chronic renal disease" or "chronic kidney failure" or "chronic renal failure"):ti,ab,kw |
|  | 3 | (CKF or CKD or CRF or CRD):ti,ab,kw |
|  | 4 | ("end-stage kidney" or "end-stage renal" or "endstage kidney" or "endstage renal"):ti,ab,kw |
|  | 5 | (ESRD or ESRF or ESKD or ESKF):ti,ab,kw |
|  | 6 | MeSH descriptor: [Renal Replacement Therapy] explode all trees |
|  | 7 | dialysis:ti,ab,kw |
|  | 8 | (hemodialysis or haemodialysis or hemodiafiltration or haemodiafiltration "hemofiltration or haemofiltration or HD):ti,ab,kw |
|  | 9 | PD:ti,ab,kw |
|  | 10 | ("renal transplantation" or "kidney grafting" or "kidney transplantation"):ti,ab,kw |
|  | 11 | KTRs:ti,ab,kw |
|  | 12 | #1 or #2 or #3 or #4 or #5 or #6 or #7 or #8 or #9 or #10 or #11 |
|  | 13 | MeSH descriptor: [Frailty] explode all trees |
|  | 14 | ("frail*" or "debilit*"):ti,ab,kw |
|  | 15 | #13 or #14 |
|  | 16 | #12 and #15 |
| 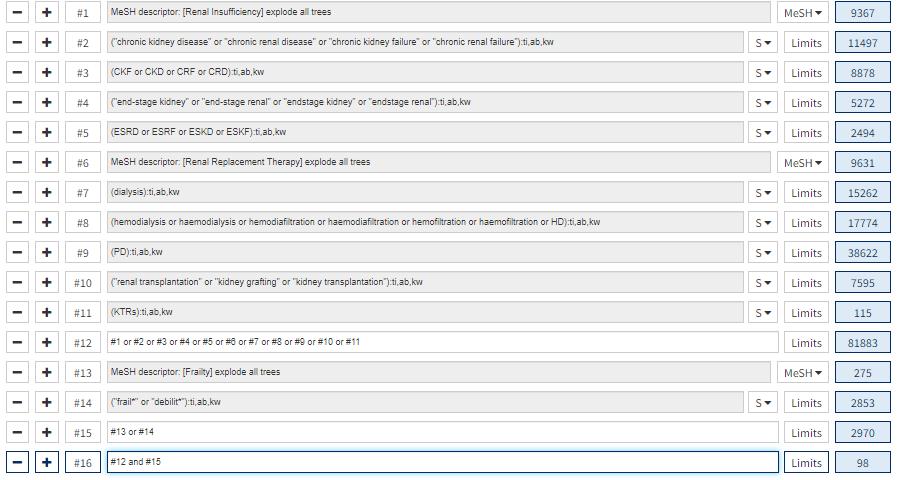 | | |
| **Embase (n=7664)** | 1 | 'kidney disease'/exp |
|  | 2 | ('chronic kidney disease' or 'chronic renal disease' or 'chronic kidney failure' or 'chronic renal failure'):ti,ab,kw |
|  | 3 | (CKF or CKD or CRFor CRD):ti,ab,kw |
|  | 4 | ('end-stage kidney' or 'end-stage renal' or 'endstage kidney' or 'endstage renal'):ti,ab,kw |
|  | 5 | (ESRD or ESRF or ESKD or ESKF):ti,ab,kw |
|  | 6 | 'renal replacement therapy'/exp |
|  | 7 | 'dialysis':ti,ab,kw |
|  | 8 | (hemodialysis or haemodialysis or hemofiltration or haemofiltration or hemodiafiltration or haemodiafiltration or HD):ti,ab,kw |
|  | 9 | PD:ti,ab,kw |
|  | 10 | ('renal transplantation' or 'kidney grafting' or 'kidney transplantation'):ti,ab,kw |
|  | 11 | KTRs:ti,ab,kw |
|  | 12 | or/1-11 |
|  | 13 | 'frailty'/exp |
|  | 14 | ('frail*' or 'debilit*'):ti,ab,kw |
|  | 15 | or/13-14 |
|  | 16 | and/12,15 |
| 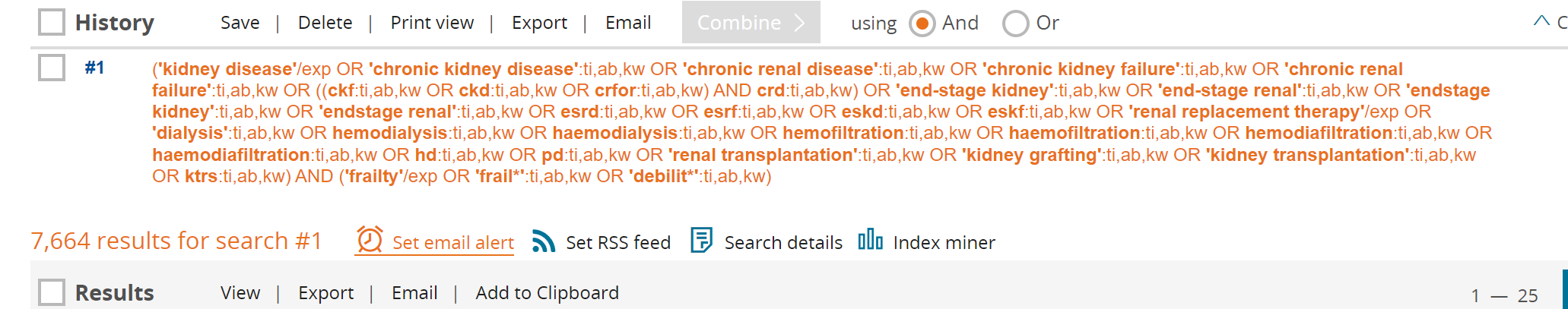 | | |
| **Web of Science (n=2576)** | 1 | TS: ("chronic kidney disease" or "chronic renal disease" or "chronic kidney failure" or "chronic renal failure" or CKD or CRD or CKF or CRF) |
|  | 2 | TS: ("end-stage kidney" or "end-stage renal" or "endstage kidney" or "endstage renal" or ESKD or ESKF or ESRD or ESRF) |
|  | 3 | TS: ("renal replacement therapy" or dialysis or hemodialysis or haemodialysis or hemofiltration or haemofiltration or hemodiafiltration or haemodiafiltration or HD or PD) |
|  | 4 | TS: ("renal transplantation" or "kidney grafting" or "kidney transplantation" or KTRs) |
|  | 5 | #1 or #2 or #3 or #4 |
|  | 6 | TS: ("frail*" or "debilit*") |
|  | 7 | #5 and #6 |
| 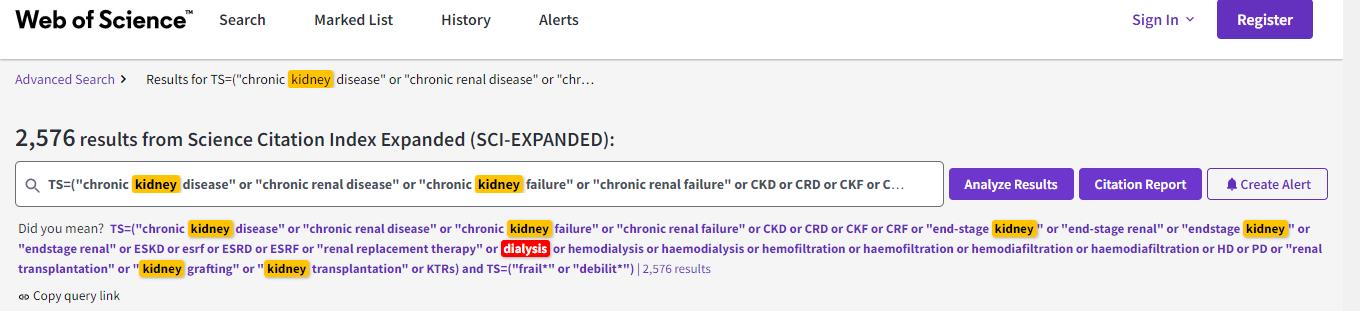 | | |
| Clinicaltrial.gov (n=11) | 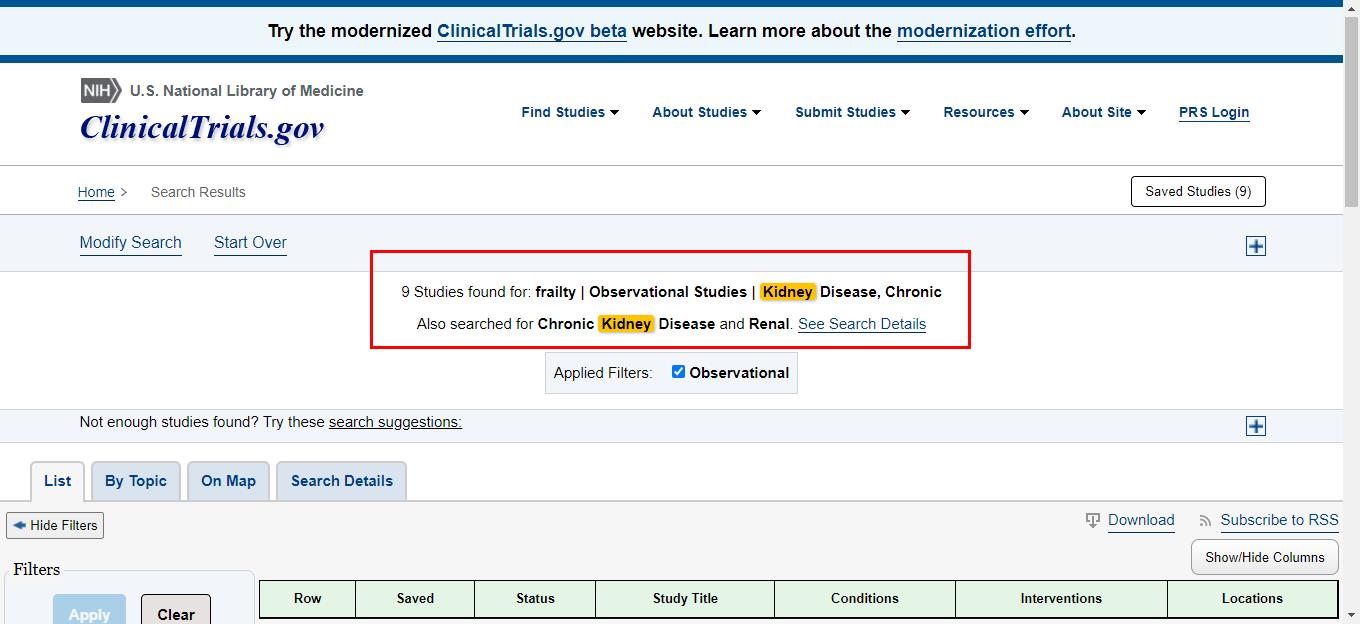 | |
|  | 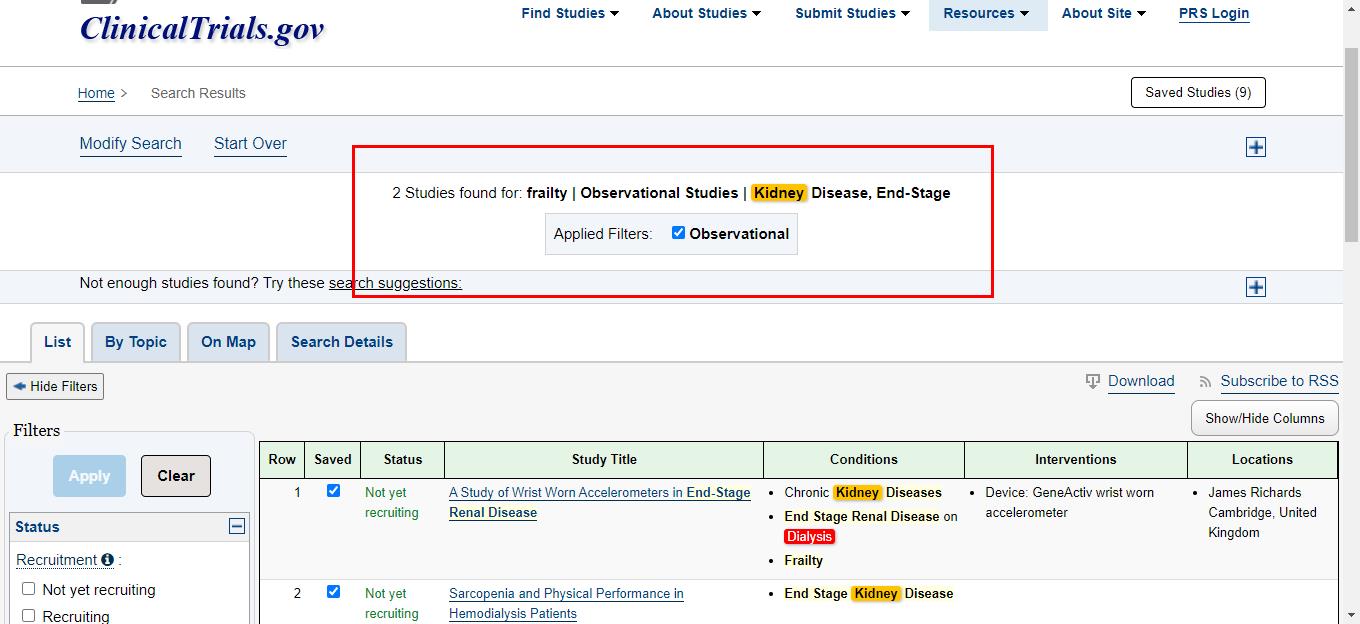 | |

Table S4 List of studies excluded at full-text review and reasons for exclusion

| NO. | References | Reasons |
| --- | --- | --- |
| 1 | Chao C, Lee Y, Li C, et al. Advanced Age and Chronic Kidney Disease Modify the Association Between Metabolic Syndrome and Frailty Among Community-Dwelling Elderly[J]. Rejuvenation Research, 2020, 23(4): 333-340. | Assessed for frailty but did not report incidence |
| 2 | Mcadams-Demarco M A, Olorundare I O, Ying H, et al. Frailty and Postkidney Transplant Health-Related Quality of Life[J]. Transplantation, 2018, 102(2): 291-299. | Assessed for frailty but did not report incidence |
| 3 | Iyasere O, Brown E, Gordon F, et al. Longitudinal Trends in Quality of Life and Physical Function in Frail Older Dialysis Patients: A Comparison of Assisted Peritoneal Dialysis and In-Center Hemodialysis[J]. Peritoneal Dialysis International: Journal of the International Society for Peritoneal Dialysis, 2019, 39(2): 112-118. | Assessed for frailty but did not report incidence |
| 4 | Yun H J, Ryoo S R, Kim J, et al. Trabecular bone score may indicate chronic kidney disease-mineral and bone disorder (CKD-MBD) phenotypes in hemodialysis patients: a prospective observational study[J]. BMC Nephrology, 2020, 21(1). | Assessed for frailty but did not report incidence |
| 5 | Goffin E, Candellier A, Vart P, et al. COVID-19-related mortality in kidney transplant and haemodialysis patients: a comparative, prospective registry-based study[J]. Nephrology Dialysis Transplantation, 2021, 36(11): 2094-2105. | Assessed for frailty but did not report incidence |
| 6 | Hendra H, Sridharan S, Farrington K, et al. Determinants of active energy expenditure in haemodialysis patients[J]. Clinical Physiology and Functional Imaging, 2022. | Assessed for frailty but did not report incidence |
| 7 | Chu N M, Deng A, Ying H, et al. Dynamic Frailty Before Kidney Transplantation: Time of Measurement Matters[J]. Transplantation, 2019, 103(8): 1700-1704. | Assessed for frailty but did not report incidence |
| 8 | Schweitzer C D, Anagnostakos J P, Nagarsheth K H. Frailty as a Predictor of Adverse Outcomes After Peripheral Vascular Surgery in Patients with End-Stage Renal Disease[J]. The American Surgeon, 2022, 88(4): 686-691. | Assessed for frailty but did not report incidence |
| 9 | Mcadams-Demarco M A, King E A, Luo X, et al. Frailty, Length of Stay, and Mortality in Kidney Transplant Recipients[J]. Annals of Surgery, 2017, 266(6): 1084-1090. | Assessed for frailty but did not report incidence |
| 10 | Hemmelder M H, Noordzij M, Vart P, et al. Recovery of dialysis patients with COVID-19: health outcomes 3 months after diagnosis in ERACODA[J]. Nephrol Dial Transplant, 2022, 37(6): 1140-1151. | Assessed for frailty but did not report incidence |
| 11 | Hwang D, Lee E, Park S, et al. Validation of risk prediction tools in elderly patients who initiate dialysis[J]. International Urology and Nephrology, 2019, 51(7): 1231-1238. | Assessed for frailty but did not report incidence |
| 12 | Karava V, Dotis J, Christoforidis A, et al. Association between insulin growth factor-1, bone mineral density, and frailty phenotype in children with chronic kidney disease[J]. Pediatric Nephrology, 2021, 36(7): 1861-1870. | Children |
| 13 | Sgambat K, Matheson M B, Hooper S R, et al. Prevalence and outcomes of fragility: a frailty-inflammation phenotype in children with chronic kidney disease[J]. Pediatric Nephrology, 2019, 34(12): 2563-2569. | Children |
| 14 | Murray, A; Hoffman, A; Miles, C et al. Coexistence of frailty in kidney transplant candidates and their care partners. 21st Annual State of the Art Winter Symposium. Am J Transplant, 2021. DOI: 10.1111/ajt.16453 | Conference Abstract |
| 15 | Chiu, V; Gross, A L; Chu, N M et al. Development and Predictive Utility of a Comprehensive Geriatric Assessment for Older Adults with Chronic Kidney Disease, 2022. DOI: 10.1111/jgs.v70. S1 | Conference Abstract |
| 16 | Segal, J B; Chang, H Y; Varadhan, R et al. Frailty and the impact of primary care on outcomes among patients with chronic renal insufficiency. Journal of General Internal Medicine, 2017 | Conference Abstract |
| 17 | Beerli, N; Gschwind, C; Binet, I et al. Gait speed as single fried frailty phenotype item predictive for length of stay but not readmission in kidney transplantation: A secondary data analysis of a multicenter, prospective cohort study. Swiss Medical Weekly, 2021. | Conference Abstract |
| 18 | Chan, K N; Watford, D J; Cheng, X S et al. Longitudinal physical performance following kidney transplant. Journal of the American Society of Nephrology, 2020 | Conference Abstract |
| 19 | Wu, H; Van Mierlo, R; Dhaygude, A P et al. Predictors of functional status change in patients with CKD between two hip fracture events: A 6-year prospective study. Journal of the American Society of Nephrology, 2020. DOI: 10.1681/asn.2019020109. | Conference Abstract |
| 20 | Wittbrodt, E; Kushner, P; Barone, S et al. Prevalence of and factors associated with undiagnosed stage 3 chronic kidney disease in patient with a history of heart failure: A report from REVEAL-CKD. European Heart Journal, 2021. DOI: 10.1093/eurheartj/ehab724.0831 | Conference Abstract |
| 21 | Zemp, D; Giannini, O; Quadri, P et al. The frailty process in CKD patients: Analysing the role of haemodialysis, 2021. Swiss Medical Weekly. | Conference Abstract |
| 22 | Liu, C; Seo, J J; Wright, K A et al. Mobility in older hemodialysis patients: A mixed methods study. Journal of the American Society of Nephrology, 2020. DOI: 10.1681/asn.2019020109. | Conference Abstract |
| 23 | Barbosa, E; Pereira, A G; Mori, V et al. Association between frailty and all-cause hospitalization and mortality during 6 months in hemodialysis patients. Clinical Nutrition ESPEN, 2021.46: S688. |  |
| 24 | Ala S, Mamashli S, Mosavi K. Demographic characteristic of hemodialysis patients in Sari, 2014[J]. Journal of Mazandaran University of Medical Sciences, 2015, 25(121):380-385. | Full text unavailable |
| 25 | Rubio, M V R; Navarro, P M; Arnal, L M L et al. Frailty in patients with conservative management of end-stage renal disease [J]. Dialisis y Trasplante, 2017, 2(38): 92-99 | Full text unavailable |
| 26 | McAdams-DeMarco MA, Ying H, Van Pilsum Rasmussen S, et al. Prehabilitation prior to kidney transplantation: Results from a pilot study. Clin Transplant[J]. 2019;33(1): e13450. | Interventional studies |
| 27 | Nixon AC, Bampouras TM, Pendleton N, et al. Erratum: Frailty is independently associated with worse health-related quality of life in chronic kidney disease: a secondary analysis of the Frailty Assessment in Chronic Kidney Disease study. Clin Kidney J. 2020;14(3):1035. | Letter and erratum |
| 28 | Sieverdes J C, Rao V, Taber D J, et al. Feasibility and Functional Testing for Frailty in Chronic Kidney Disease[J]. Progress in Transplantation, 2018, 28(3): 299-300. | Letter and erratum |
| 29 | Jung H, Choi I Y, Shin D W, et al. Association between physical performance and incidence of end-stage renal disease in older adults: a national wide cohort study[J]. BMC Nephrology, 2021, 22(1). | Non-CKD |
| 30 | Liu Y, Yang J, Wang H, et al. Association between the combined effect of frailty and the estimated glomerular filtration rate and non-elective hospital readmission in elderly inpatients: a cohort study[J]. Annals of Palliative Medicine, 2022, 11(2): 766-773. | Non-CKD |
| 31 | Wang M, Sun X, Zhang W, et al. Frailty and the risk of kidney function decline in the elderly population: the Rugao Longevity and Ageing Study[J]. Nephrology Dialysis Transplantation, 2021, 36(12): 2274-2281. | Non-CKD |
| 32 | Chao C, Wang J, Huang J, et al. Frailty predicts a higher risk of incident urolithiasis in 525 368 patients with diabetes mellitus: a population-based study[J]. BMJ Open Diabetes Research & Care, 2020, 8(1): e755. | Non-CKD |
| 33 | Aitken S J, Lujic S, Randall D A, et al. Predicting outcomes in older patients undergoing vascular surgery using the Hospital Frailty Risk Score[J]. British journal of surgery, 2020, 108(6): 659-666. | Non-CKD |
| 34 | Xie J, Hu Z, Sun Y, et al. Frailty Score and Renal Function Impairment in Elderly Patients with Hypertension: A Correlation Study[J]. Journal of Medical Imaging and Health Informatics, 2020, 10(8): 1809-1814. | Non-CKD |
| 35 | Kanauchi M, Kubo A, Kanauchi K, et al. Frailty, health-related quality of life and mental well-being in older adults with cardiometabolic risk factors[J]. International Journal of Clinical Practice, 2008, 62(9): 1447-1451. | Non-CKD |
| 36 | Yip K F, Wong T H, Alhamid S M, et al. Integrating advance care planning as part of comprehensive geriatric assessment for hospitalised frail elderly patients: findings of a cross-sectional study[J]. Singapore Medical Journal, 2020, 61(5): 254-259. | Non-CKD |
| 37 | Noto-Kadou-Kaza B, Teuwafeu DG, Sabi KA, et al. Chutes chez l'hémodialysé : incidence et facteurs de risque [Falls among hemodialysis patients: Incidence and risk factors]. Nephrol Ther. 2015;11(4):246-249. | Non-English |
| 38 | Nogueira Á, Álvarez G, Russo F, et al. Is SPPB useful as a screening method of functional capacity in patients with advanced chronic kidney disease Es útil el SPPB como método de screening de capacidad funcional en pacientes con enfermedad renal crónica avanzada?. Nefrologia (Engl Ed). 2019;39(5):489-496. | Non-English |
| 39 | Javier Loaiza-Félix, Saúl Huerta-Ramírez, César Iván Elizalde. [Proteinuria in patients with diabetic nephropathy and its association to fragility syndrome]. Medicina Interna de Mexico. 31(3): 233-240. | Non-English |
| 40 | Ríos Á, Herrera P, Morales Á, et al. Análisis de sobrevida en pacientes de edad avanzada que inician hemodiálisis crónica en Servicio de Salud Chileno [Survival of older patients starting hemodialysis in Chile]. Rev Med Chil. 2016;144(6):697-703. | Non-English |
| 41 | Gesualdo GD, Zazzetta MS, Say KG, et al. Fatores associados à fragilidade de idosos com doença renal crônica em hemodiálise [Factors associated with the frailty of elderly people with chronic kidney disease on hemodialysis]. Cien Saude Colet. 2016;21(11):3493-3498. | Non-English |
| 42 | Laurentius, T., Freitag, M., Maassen, B. et al. [Geriatrisches Basis-Assessment, Frailty und Sarkopenie]. Nephrologe. 2018; 13: 299–305. | Non-English |
| 43 | Heras Benito M. Impacto de la modalidad de terapia de reemplazo renal en adultos mayores frágiles: importancia de las preferencias del paciente [Impact of renal replacement therapy on frail older adults: Importance of patient's preferences]. Rev Esp Geriatr Gerontol. 2020;55(4):242. | Non-English |
| 44 | Jovic D, Dimkovic N, Rakocevic I, et al. Prevalence and factors associated with self-reported kidney disease among Serbian adults: Results of 2013 National Health Survey[J]. PLoS One, 2018, 13(9): e203620. | Non-physician diagnosed CKD |
| 45 | Margiotta E, Caldiroli L, Callegari M L, et al. Association of Sarcopenia and Gut Microbiota Composition in Older Patients with Advanced Chronic Kidney Disease, Investigation of the Interactions with Uremic Toxins, Inflammation and Oxidative Stress[J]. Toxins, 2021, 13(7): 472. | Non-reported frailty assessment tool |
| 46 | Arce C M, Mitani A A, Goldstein B A, et al. Hispanic Ethnicity and Vascular Access Use in Patients Initiating Hemodialysis in the United States[J]. Clinical journal of the American Society of Nephrology, 2012, 7(2): 289-296. | Non-reported frailty assessment tool |
| 47 | Fonseca-Correa J I, Farragher J F, Tomlinson G, et al. Longitudinal Changes in the Use of PD Assistance for Patients Maintained on Peritoneal Dialysis[J]. Kidney360, 2021, 2(3): 469-476. | Non-reported frailty assessment tool |
| 48 | Reid J, Noble HR, Adamson G, et al. Establishing a clinical phenotype for cachexia in end stage kidney disease - study protocol. BMC Nephrol[J]. 2018;19(1):38. | Protocol |
| 49 | Portilla Franco ME, Tornero Molina F, Gil Gregorio P. Frailty in elderly people with chronic kidney disease. La fragilidad en el anciano con enfermedad renal crónica. Nefrologia[J]. 2016;36(6):609-615. | Review |
| 50 | Soni R K, Weisbord S D, Unruh M L. Health-related quality of life outcomes in chronic kidney disease[J]. Current Opinion in Nephrology and Hypertension, 2010, 19(2): 153-159. | Review |
| 51 | Mcadams-Demarco M A, Chu N M, Segev D L. Frailty and Long-Term Post-Kidney Transplant Outcomes[J]. Current Transplantation Reports, 2019, 6(1): 45-51. | Review |
| 52 | Cheng X S, Lentine K L, Koraishy F M, et al. Implications of Frailty for Peritransplant Outcomes in Kidney Transplant Recipients[J]. Current Transplantation Reports, 2019, 6(1): 16-25. | Review |
| 53 | Lo D, Chiu E, Jassal S V. A Prospective Pilot Study to Measure Changes in Functional Status Associated With Hospitalization in Elderly Dialysis-Dependent Patients[J]. American Journal of Kidney Diseases, 2008, 52(5): 956-961. | No reported prevalence of frailty |
| 54 | Jaques D A, Henderson S, Davenport A. Association between bone mineral density at different anatomical sites and both mortality and fracture risk in patients receiving renal replacement therapy: a longitudinal study[J]. Clinical Kidney Journal, 2022, 15(6): 1188-1195. | No reported prevalence of frailty |
| 55 | Hanafusa N, Kamei D, Tsukada M, et al. Association between Increases in Normalized Protein Catabolic Rate and Increases in Creatinine Generation Rate in Dialysis Patients[J]. Contributions to nephrology, 2018, 195: 51. | No reported prevalence of frailty |
| 56 | Matsufuji S, Shoji T, Lee S, et al. Association between Levocarnitine Treatment and the Change in Knee Extensor Strength in Patients Undergoing Hemodialysis: A Post-Hoc Analysis of the Osaka Dialysis Complication Study (ODCS)[J]. Nutrients, 2022, 14(2): 343. | No reported prevalence of frailty |
| 57 | Lai S, Amabile M, Mazzaferro S, et al. Association between Multidimensional Prognostic Index and Hospitalization and Mortality among Older Adults with Chronic Kidney Disease on Conservative or on Replacement Therapy[J]. Journal of Clinical Medicine, 2020, 9(12): 3965. | No reported prevalence of frailty |
| 58 | Michelson A T, Tsapepas D S, Husain S A, et al. Association between the “Timed Up and Go Test” at transplant evaluation and outcomes after kidney transplantation[J]. Clinical Transplantation, 2018, 32(11): e13410. | No reported prevalence of frailty |
| 59 | Kuki A, Tanaka K, Kushiyama A, et al. Association of gait speed and grip strength with risk of cardiovascular events in patients on haemodialysis: a prospective study[J]. BMC Nephrology, 2019, 20(1). | No reported prevalence of frailty |
| 60 | Chen D C, Shlipak M G, Scherzer R, et al. Association of Intraindividual Difference in Estimated Glomerular Filtration Rate by Creatinine vs Cystatin C and End-stage Kidney Disease and Mortality[J]. JAMA Network Open, 2022, 5(2): e2148940. | No reported prevalence of frailty |
| 61 | Sheshadri A, Cullaro G, Johansen K L, et al. Association of Karnofsky Performance Status with waitlist mortality among older and younger adults awaiting kidney transplantation[J]. Clin Transplant, 2020, 34(6): e13848. | No reported prevalence of frailty |
| 62 | Sonnenberg E M, Cohen J B, Hsu J Y, et al. Association of Kidney Transplant Center Volume With 3-Year Clinical Outcomes[J]. American Journal of Kidney Diseases, 2019, 74(4): 441-451. | No reported prevalence of frailty |
| 63 | Myers J, Chan K N, Chen Y, et al. Association of physical function and performance with peak VO2 in elderly patients with end stage kidney disease[J]. Aging Clinical and Experimental Research, 2021, 33(10): 2797-2806. | No reported prevalence of frailty |
| 64 | Kimura H, Tanaka K, Saito H, et al. Association of Polypharmacy with Kidney Disease Progression in Adults with CKD[J]. Clin J Am Soc Nephrol, 2021, 16(12): 1797-1804. | No reported prevalence of frailty |
| 65 | Plantinga L C, Lynch R J, Patzer R E, et al. Association of Serious Fall Injuries among United States End Stage Kidney Disease Patients with Access to Kidney Transplantation[J]. Clinical Journal of the American Society of Nephrology, 2018, 13(4): 628-637. | No reported prevalence of frailty |
| 66 | Nakaya R, Shoji T, Nagata Y, et al. Associations of Serum Insulin-Like Growth Factor 1 with New Cardiovascular Events and Subsequent Death in Hemodialysis Patients: The DREAM Cohort[J]. Journal of Atherosclerosis and Thrombosis, | No reported prevalence of frailty |
| 67 | Sacher F, Jesel L, Borni-Duval C, et al. Cardiac Rhythm Disturbances in Hemodialysis Patients: Early Detection Using an Implantable Loop Recorder and Correlation With Biological and Dialysis Parameters. JACC Clin Electrophysiol[J]. 2018;4(3):397-408. | No reported prevalence of frailty |
| 68 | Bäck C, Hornum M, Møller C J H, et al. Cardiac surgery in patients with end-stage renal disease on dialysis[J]. Scandinavian cardiovascular journal: SCJ, 2017, 51(6): 334-338. | No reported prevalence of frailty |
| 69 | Ivarsson K M, Akaberi S, Isaksson E, et al. Cardiovascular and Cerebrovascular Events After Parathyroidectomy in Patients on Renal Replacement Therapy[J]. World Journal of Surgery, 2019, 43(8): 1981-1988. | No reported prevalence of frailty |
| 70 | de Jager D J, Grootendorst D C, Jager K J, et al. Cardiovascular and noncardiovascular mortality among patients starting dialysis[J]. JAMA, 2009, 302(16): 1782-1789. | No reported prevalence of frailty |
| 71 | Chan W, Jones D, Bosch J A, et al. Cardiovascular, muscular and perceptual contributions to physical fatigue in prevalent kidney transplant recipients[J]. Transpl Int, 2016, 29(3): 338-351. | No reported prevalence of frailty |
| 72 | Dimkovic NB, Prakash S, Roscoe J, et al. Chronic peritoneal dialysis in octogenarians. Nephrol Dial Transplant[J]. 2001;16(10):2034-2040. | No reported prevalence of frailty |
| 73 | Isoyama N, Qureshi A R, Avesani C M, et al. Comparative Associations of Muscle Mass and Muscle Strength with Mortality in Dialysis Patients[J]. Clinical Journal of the American Society of Nephrology, 2014, 9(10): 1720-1728. | No reported prevalence of frailty |
| 74 | Renaud C J, Francois M, Nony A, et al. Comparative outcomes of treated symptomatic versus non-treated asymptomatic high-grade central vein stenoses in the outflow of predominantly dialysis fistulas[J]. Nephrol Dial Transplant, 2012, 27(4): 1631-1638. | No reported prevalence of frailty |
| 75 | Srivastava K, Tudu J, Das R C, et al. Crosssectional study of quality of life after renal transplant in end stage renal disease[J]. Industrial Psychiatry Journal, 2014, 23(1): 40. | No reported prevalence of frailty |
| 76 | Dion M, Cristea O, Langford S, et al. Debilitating Lower Urinary Tract Symptoms in the Post-Renal Transplant Population Can Be Predicted Pretransplantation[J]. Transplantation, 2013, 95(4): 589-594. | No reported prevalence of frailty |
| 77 | Matsuzawa R, Kamitani T, Roshanravan B, et al. Decline in the Functional Status and Mortality in Patients on Hemodialysis: Results from the Japan Dialysis Outcome and Practice Patterns Study[J]. Journal of Renal Nutrition, 2019, 29(6): 504-510. | No reported prevalence of frailty |
| 78 | Arai Y, Kanda E, Kikuchi H, et al. Decreased mobility after starting dialysis is an independent risk factor for short-term mortality after initiation of dialysis[J]. Nephrology (Carlton), 2014, 19(4): 227-233. | No reported prevalence of frailty |
| 79 | de C·ssia Hel Mendonáa Ribeiro R, Santiago E, Comelis D, et al. Depression in the elderly on hemodialysis for chronic renal failure[J]. Acta Paul Enferm, 2009, 22: 505-508. | No reported prevalence of frailty |
| 80 | Son Y, Choi K, Park Y, et al. Depression, Symptoms and the Quality of Life in Patients on Hemodialysis for End-Stage Renal Disease[J]. American Journal of Nephrology, 2008, 29(1): 36-42. | No reported prevalence of frailty |
| 81 | Matsuzawa R, Suzuki Y, Yamamoto S, et al. Determinants of Health-Related Quality of Life and Physical Performance-Based Components of Frailty in Patients Undergoing Hemodialysis[J]. Journal of Renal Nutrition, 2021, 31(5): 529-536. | No reported prevalence of frailty |
| 82 | Brar A, Mallappallil M, Stefanov D G, et al. Dialysis in the Elderly and Impact of Institutionalization in the United States Renal Data System[J]. American Journal of Nephrology, 2017, 46(2): 114-119. | No reported prevalence of frailty |
| 83 | Wang Y, Heemskerk M B A, Michels W M, et al. Donor type and 3-month hospital readmission following kidney transplantation: results from the Netherlands organ transplant registry[J]. BMC Nephrology, 2021, 22(1). | No reported prevalence of frailty |
| 84 | Bonfante G M, Gomes I C, Andrade E I, et al. Duration of temporary catheter use for hemodialysis: an observational, prospective evaluation of renal units in Brazil[J]. BMC Nephrol, 2011, 12: 63. | No reported prevalence of frailty |
| 85 | Haugen C E, King E A, Bae S, et al. Early Hospital Readmission in Older and Younger Kidney Transplant Recipients[J]. American Journal of Nephrology, 2018, 48(4): 235-241. | No reported prevalence of frailty |
| 86 | Yazawa M, Kido R, Ohira S, et al. Early Mortality Was Highly and Strongly Associated with Functional Status in Incident Japanese Hemodialysis Patients: A Cohort Study of the Large National Dialysis Registry[J]. PLOS ONE, 2016, 11(6): e156951. | No reported prevalence of frailty |
| 87 | Kim J E, Lee Y H, Huh J H, et al. Early-stage chronic kidney disease, insulin resistance, and osteoporosis as risk factors of sarcopenia in aged population: The Fourth Korea National Health and Nutrition Examination Survey (KNHANES IV), 2008–2009[J]. Osteoporosis International, 2014, 25(9): 2189-2198. | No reported prevalence of frailty |
| 88 | Sakkas G K, Kent-Braun J A, Doyle J W, et al. Effect of Diabetes Mellitus on Muscle Size and Strength in Patients Receiving Dialysis Therapy[J]. American Journal of Kidney Diseases, 2006, 47(5): 862-869. | No reported prevalence of frailty |
| 89 | Chen J C, Thorsteinsdottir B, Vaughan L E, et al. End of Life, Withdrawal, and Palliative Care Utilization among Patients Receiving Maintenance Hemodialysis Therapy[J]. Clinical Journal of the American Society of Nephrology, 2018, 13(8): 1172-1179. | No reported prevalence of frailty |
| 90 | Silverberg D, Yalon T, Rimon U, et al. Endovascular treatment of lower extremity ischemia in chronic renal failure patients on dialysis: early and intermediate term results[J]. Isr Med Assoc J, 2013, 15(12): 734-738. | No reported prevalence of frailty |
| 91 | Chhokra M, Manocha S, Dodwad V, et al. Establishing an Association between Renal Failure and Periodontal Health: A Cross Sectional Study[J]. Journal of clinical and diagnostic research, 2013, 7(10): 2348-2350. | No reported prevalence of frailty |
| 92 | Alkhaqani A L, Ali D K A. Evaluate of the Physical Performance of Patients Undergoing Hemodialysis[J]. Journal of Cardiovascular Disease Research, 2021, 12(6): 197-205. | No reported prevalence of frailty |
| 93 | Tabata A, Yabe H, Katogi T, et al. Factors affecting health-related quality of life in older patients with chronic kidney disease: a single-center cross-sectional study[J]. International Urology and Nephrology, 2022. | No reported prevalence of frailty |
| 94 | Kutner N G, Zhang R, Huang Y, et al. Falls among hemodialysis patients: potential opportunities for prevention?[J]. Clinical Kidney Journal, 2014, 7(3): 257-263. | No reported prevalence of frailty |
| 95 | Cook W L, Tomlinson G, Donaldson M, et al. Falls and fall-related injuries in older dialysis patients[J]. Clin J Am Soc Nephrol, 2006, 1(6): 1197-1204. | No reported prevalence of frailty |
| 96 | Manay P, Ten Eyck P, Kalil R, et al. Frailty measures can be used to predict the outcome of kidney transplant evaluation[J]. Surgery, 2021, 169(3): 686-693. | No reported prevalence of frailty |
| 97 | Pladys A, Bayat S, Kolko A, et al. French patients on daily hemodialysis: clinical characteristics and treatment trajectories[J]. BMC Nephrology, 2016, 17(1). | No reported prevalence of frailty |
| 98 | Chao C, Huang J, Chiang C. Functional assessment of chronic illness therapy—the fatigue scale exhibits stronger associations with clinical parameters in chronic dialysis patients compared to other fatigue-assessing instruments[J]. PeerJ, 2016, 4: e1818. | No reported prevalence of frailty |
| 99 | Chu N M, Sison S, Muzaale A D, et al. Functional independence, access to kidney transplantation and waitlist mortality[J]. Nephrology Dialysis Transplantation, 2020, 35(5): 870-877. | No reported prevalence of frailty |
| 100 | Shah S, Leonard A C, Thakar C V. Functional status, pre-dialysis health and clinical outcomes among elderly dialysis patients[J]. BMC Nephrology, 2018, 19(1). | No reported prevalence of frailty |
| 101 | Kutner N G, Zhang R, Huang Y, et al. Gait Speed and Mortality, Hospitalization, and Functional Status Change Among Hemodialysis Patients: A US Renal Data System Special Study[J]. American Journal of Kidney Diseases, 2015, 66(2): 297-304. | No reported prevalence of frailty |
| 102 | Formiga F, Ferrer A, Cruzado J M, et al. Geriatric assessment and chronic kidney disease in the oldest old: The Octabaix study[J]. European Journal of Internal Medicine, 2012, 23(6): 534-538. | No reported prevalence of frailty |
| 103 | Delanaye P, Quinonez K, Buckinx F, et al. Hand grip strength measurement in haemodialysis patients: before or after the session[J]. Clin Kidney J, 2018, 11(4): 555-558. | No reported prevalence of frailty |
| 104 | Jesky M D, Dutton M, Dasgupta I, et al. Health-Related Quality of Life Impacts Mortality but Not Progression to End-Stage Renal Disease in Pre-Dialysis Chronic Kidney Disease: A Prospective Observational Study[J]. PLOS ONE, 2016, 11(11): e165675. | No reported prevalence of frailty |
| 105 | Marshall M R, Polkinghorne K R, Boudville N, et al. Home Versus Facility Dialysis and Mortality in Australia and New Zealand[J]. American Journal of Kidney Diseases, 2021, 78(6): 826-836. | No reported prevalence of frailty |
| 106 | Molnar A O, Moist L, Klarenbach S, et al. Hospitalizations in Dialysis Patients in Canada: A National Cohort Study[J]. Canadian Journal of Kidney Health and Disease, 2017, 5: 1063824235. | No reported prevalence of frailty |
| 107 | Yeoh L Y, Seow Y Y, Tan H C. Identifying high-risk hospitalised chronic kidney disease patient usingelectronic health records for serious illness conversation[J]. Annals of the Academy of Medicine, Singapore, 2022, 51(3): 161-169. | No reported prevalence of frailty |
| 108 | Villain C, Ecochard R, Genet L, et al. Impact of BMI Variations on Survival in Elderly Hemodialysis Patients[J]. Journal of Renal Nutrition, 2015, 25(6): 488-493. | No reported prevalence of frailty |
| 109 | Notomi S, Kitamura M, Yamaguchi K, et al. Impact of Cafeteria Service Discontinuation at a Dialysis Facility on Medium-Term Nutritional Status of Elderly Patients Undergoing Hemodialysis[J]. Nutrients, 2022, 14(8): 1628. | No reported prevalence of frailty |
| 110 | Onishi Y, Uchida H A, Takeuchi H, et al. Impaired mental health status in patients with chronic kidney disease is associated with estimated glomerular filtration rate decline[J]. Nephrology, 2019. | No reported prevalence of frailty |
| 111 | Iseri K, Carrero J J, Evans M, et al. Incidence of Fractures Before and After Dialysis Initiation[J]. Journal of Bone and Mineral Research, 2020, 35(12): 2372-2380. | No reported prevalence of frailty |
| 112 | Portolés J, Vega A, Lacoba E, et al. Is peritoneal dialysis suitable technique CKD patients over 65 years? A prospective multicenter study[J]. Nefrología, 2021, 41(5): 529-538. | No reported prevalence of frailty |
| 113 | Taylor K, Chu N M, Chen X, et al. Kidney Disease Symptoms before and after Kidney Transplantation[J]. Clinical journal of the American Society of Nephrology, 2021, 16(7): 1083-1093. | No reported prevalence of frailty |
| 114 | Daya N R, Voskertchian A, Schneider A L C, et al. Kidney Function and Fracture Risk: The Atherosclerosis Risk in Communities (ARIC) Study[J]. American Journal of Kidney Diseases, 2016, 67(2): 218-226. | No reported prevalence of frailty |
| 115 | Udayaraj U P, Haynes R, Winearls C G. Late presentation of patients with end-stage renal disease for renal replacement therapy--is it always avoidable?[J]. Nephrology Dialysis Transplantation, 2011, 26(11): 3646-3651. | No reported prevalence of frailty |
| 116 | Molfino A, Amabile M I, Ammann T, et al. Longitudinal Physical Activity Change During Hemodialysis and Its Association With Body Composition and Plasma BAIBA Levels[J]. Frontiers in Physiology, 2019, 10. | No reported prevalence of frailty |
| 117 | Adachi H, Fujimoto K, Fujii A, et al. Long-term retrospective observation study to evaluate effects of adiponectin on skeletal muscle in renal transplant recipients[J]. Scientific Reports, 2020, 10(1). | No reported prevalence of frailty |
| 118 | Kovarova L, Valerianova A, Kmentova T, et al. Low Cerebral Oxygenation Is Associated with Cognitive Impairment in Chronic Hemodialysis Patients[J]. Nephron, 2018, 139(2): 113-119. | No reported prevalence of frailty |
| 119 | Marcus R L, Lastayo P C, Ikizler T A, et al. Low Physical Function in Maintenance Hemodialysis Patients Is Independent of Muscle Mass and Comorbidity[J]. Journal of Renal Nutrition, 2015, 25(4): 371-375. | No reported prevalence of frailty |
| 120 | Plantinga L C, Johansen K L, Schillinger D, et al. Lower socioeconomic status and disability among US adults with chronic kidney disease, 1999-2008[J]. Preventing chronic disease, 2012, 9: E12. | No reported prevalence of frailty |
| 121 | Otobe Y, Hiraki K, Hotta C, et al. Mild cognitive impairment in older adults with pre‐dialysis patients with chronic kidney disease: Prevalence and association with physical function[J]. Nephrology, 2018, 24(1): 50-55. | No reported prevalence of frailty |
| 122 | Viscogliosi G, De Nicola L, Vanuzzo D, et al. Mild to moderate chronic kidney disease and functional disability in community-dwelling older adults. The Cardiovascular risk profile in Renal patients of the Italian Health Examination Survey (CARHES) study[J]. Archives of Gerontology and Geriatrics, 2019, 80: 46-52. | No reported prevalence of frailty |
| 123 | Bossola M, Pepe G, Antocicco M, et al. Mini‐Mental State Examination predicts mortality in patients on chronic hemodialysis[J]. Seminars in Dialysis, 2022. | No reported prevalence of frailty |
| 124 | Moorthi R N, Fadel W F, Cranor A, et al. Mobility Impairment in Patients New to Dialysis[J]. American Journal of Nephrology, 2020, 51(9): 705-714. | No reported prevalence of frailty |
| 125 | Terjimanian M N, Underwood P W, Cron D C, et al. Morphometric age and survival following kidney transplantation[J]. Clinical Transplantation, 2017, 31(10). | No reported prevalence of frailty |
| 126 | Htay H, Cho Y, Pascoe E M, et al. Multicenter Registry Analysis of Center Characteristics Associated with Technique Failure in Patients on Incident Peritoneal Dialysis[J]. Clinical Journal of the American Society of Nephrology, 2017, 12(7): 1090-1099. | No reported prevalence of frailty |
| 127 | Puvanesarajah V, Amin R, Qureshi R, et al. Outcomes following surgical management of femoral neck fractures in elderly dialysis-dependent patients[J]. Archives of Orthopaedic and Trauma Surgery, 2018, 138(6): 757-764. | No reported prevalence of frailty |
| 128 | Woo K, Gascue L, Norris K, et al. Patient Frailty and Functional Use of Hemodialysis Vascular Access: A Retrospective Study of the US Renal Data System[J]. American Journal of Kidney Diseases, 2022, 80(1): 30-45. | No reported prevalence of frailty |
| 129 | Ifudu O, Paul H, Mayers J D, et al. Pervasive Failed Rehabilitation in Center-Based Maintenance Hemodialysis Patients[J]. American Journal of Kidney Diseases, 1994, 23(3): 394-400. | No reported prevalence of frailty |
| 130 | Gutman R A, Stead W W, Robinson R R. Physical activity and employment status of patients on maintenance dialysis[J]. N Engl J Med, 1981, 304(6): 309-313. | No reported prevalence of frailty |
| 131 | Matsuzawa R, Roshanravan B, Shimoda T, et al. Physical Activity Dose for Hemodialysis Patients: Where to Begin? Results from a Prospective Cohort Study[J]. Journal of Renal Nutrition, 2018, 28(1): 45-53. | No reported prevalence of frailty |
| 132 | Okubo R, Kondo M, Tsunoda R, et al. Physical functioning in patients with chronic kidney disease stage G3b-5 in Japan: Thereach-J CKD cohort study[J]. Nephrology, 2021, 26(12): 981-987. | No reported prevalence of frailty |
| 133 | Joo Y S, Jhee J H, Kim H W, et al. Physical performance and chronic kidney disease development in elderly adults: results from a nationwide cohort study[J]. Aging (Albany NY), 2020, 12(17): 17393-17417. | No reported prevalence of frailty |
| 134 | Wilkinson T J, Nixon D G D, Smith A C. Postural stability during standing and its association with physical and cognitive functions in non-dialysis chronic kidney disease patients[J]. International Urology and Nephrology, 2019, 51(8): 1407-1414. | No reported prevalence of frailty |
| 135 | Lee J, Turin T C, Nicholl D D M, et al. Predictors of successful completion of diagnostic home sleep testing in patients with chronic kidney disease[J]. Sleep and Breathing, 2015, 19(2): 669-675. | No reported prevalence of frailty |
| 136 | Stack S, Chertow G M, Johansen K L, et al. Pre-ESRD Changes in Body Weight and Survival in Nursing Home Residents Starting Dialysis[J]. Clinical journal of the American Society of Nephrology, 2013, 8(10): 1734-1740. | No reported prevalence of frailty |
| 137 | Nastasi A J, Bryant T S, Le J T, et al. Pre-kidney transplant lower extremity impairment and transplant length of stay: a time-to-discharge analysis of a prospective cohort study[J]. BMC Geriatrics, 2018, 18(1). | No reported prevalence of frailty |
| 138 | Harhay M N, Chen X, Chu N M, et al. Pre-kidney transplant unintentional weight loss leads to worse post-kidney transplant outcomes[J]. Nephrology Dialysis Transplantation, 2021, 36(10): 1927-1936. | No reported prevalence of frailty |
| 139 | Kim J, Choi S R, Choi M J, et al. Prevalence of and factors associated with sarcopenia in elderly patients with end-stage renal disease[J]. Clinical Nutrition, 2014, 33(1): 64-68. | No reported prevalence of frailty |
| 140 | Saucedo-Crespo H, Haakinson D J, Croome K P, et al. Prognostic factors in kidney transplantation in the septuagenarian: a multicenter analysis[J]. Clinical Transplantation, 2016, 30(7): 828-835. | No reported prevalence of frailty |
| 141 | De Biase V, Tobaldini O, Boaretti C, et al. Prolonged conservative treatment for frail elderly patients with end-stage renal disease: the Verona experience[J]. Nephrology Dialysis Transplantation, 2007, 23(4): 1313-1317. | No reported prevalence of frailty |
| 142 | Neto A W G, Boslooper-Meulenbelt K, Geelink M, et al. Protein Intake, Fatigue and Quality of Life in Stable Outpatient Kidney Transplant Recipients[J]. Nutrients, 2020, 12(8): 2451. | No reported prevalence of frailty |
| 143 | van Loon I N, Bots M L, Boereboom F T J, et al. Quality of life as indicator of poor outcome in hemodialysis: relation with mortality in different age groups[J]. BMC Nephrology, 2017, 18(1). | No reported prevalence of frailty |
| 144 | Gilbertson D T, Monda K L, Bradbury B D, et al. RBC Transfusions Among Hemodialysis Patients (1999-2010): Influence of Hemoglobin Concentrations Below 10 g/dL[J]. American Journal of Kidney Diseases, 2013, 62(5): 919-928. | No reported prevalence of frailty |
| 145 | Kojima M, Inaguma D, Koide S, et al. Relationship between History of Ischemic Stroke and All-Cause Mortality in Incident Dialysis Patients[J]. Nephron, 2019, 143(1): 43-53. | No reported prevalence of frailty |
| 146 | Lemoine M, Titeca B D, Lobbedez T, et al. Risk Factors for Early Graft Failure and Death After Kidney Transplantation in Recipients Older Than 70 Years[J]. Kidney Int Rep, 2019, 4(5): 656-666. | No reported prevalence of frailty |
| 147 | Hall R K, Luciano A, Pendergast J F, et al. Self-reported Physical Function Decline and Mortality in Older Adults Receiving Hemodialysis[J]. Kidney Medicine, 2019, 1(5): 288-295. | No reported prevalence of frailty |
| 148 | Mahmood U, Healy H G, Kark A, et al. Spectrum (characteristics) of patients with chronic kidney disease (CKD) with increasing age in a major metropolitan renal service[J]. BMC Nephrology, 2017, 18(1). | No reported prevalence of frailty |
| 149 | Bataille S, Landrier J, Astier J, et al. The “Dose-Effect” Relationship Between 25-Hydroxyvitamin D and Muscle Strength in Hemodialysis Patients Favors a Normal Threshold of 30 ng/mL for Plasma 25-Hydroxyvitamin D[J]. Journal of Renal Nutrition, 2016, 26(1): 45-52. | No reported prevalence of frailty |
| 150 | van Diepen A T N, Tomlinson G A, Jassal S V. The Association between Exit Site Infection and Subsequent Peritonitis among Peritoneal Dialysis Patients[J]. Clinical journal of the American Society of Nephrology, 2012, 7(8): 1266-1271. | No reported prevalence of frailty |
| 151 | Ko E J, Kim Y K, Cho J, et al. The differential effects of anemia on mortality in young and elderly end-stage renal disease patients[J]. Kidney Research and Clinical Practice, 2020, 39(2): 192-201. | No reported prevalence of frailty |
| 152 | Bai Y, Lai L, Lee B, et al. The impact of depression on fatigue in patients with haemodialysis: a correlational study[J]. Journal of Clinical Nursing, 2015, 24(13-14): 2014-2022. | No reported prevalence of frailty |
| 153 | Hung L, Hwang Y, Huang G, et al. The influence of renal dialysis and hip fracture sites on the 10-year mortality of elderly hip fracture patients[J]. Medicine, 2017, 96(37): e7618. | No reported prevalence of frailty |
| 154 | Turkistani I, Nuqali A, Badawi M, et al. The prevalence of anxiety and depression among end-stage renal disease patients on hemodialysis in Saudi Arabia[J]. Renal failure, 2014, 36(10): 1510-1515. | No reported prevalence of frailty |
| 155 | Junqué Jiménez A, Esteve Simó V, Andreu Periz L, et al. The Relationship between Physical Activity Levels and Functional Capacity in Patients with Advanced Chronic Kidney Disease[J]. Clinical Nursing Research, 2021, 30(3): 360-368. | No reported prevalence of frailty |
| 156 | Nakazato Y, Kurane R, Hirose S, et al. Variability of laboratory parameters is associated with frailty markers and predicts non-cardiac mortality in hemodialysis patients[J]. Clinical and Experimental Nephrology, 2015, 19(6): 1165-1178. | No reported prevalence of frailty |
| 157 | Schold J D, Meier-Kriesche H U. Which renal transplant candidates should accept marginal kidneys in exchange for a shorter waiting time on dialysis?[J]. Clin J Am Soc Nephrol, 2006, 1(3): 532-538. | No reported prevalence of frailty |
| 158 | Rebollo Rubio A, Morales Asencio J M, Pons Raventos M E. Biomarkers associated with mortality in patients undergoing dialysis[J]. Journal of renal care, 2017, 43(3): 163-174. | No reported prevalence of frailty |
| 159 | Rampersad C, Darcel J, Harasemiw O, et al. Change in Physical Activity and Function in Patients with Baseline Advanced Nondialysis CKD[J]. Clin J Am Soc Nephrol, 2021. | No reported prevalence of frailty |
| 160 | Chu N M, Chen X, Bae S, et al. Changes in Functional Status Among Kidney Transplant Recipients: Data From the Scientific Registry of Transplant Recipients[J]. Transplantation, 2021, 105(9): 2104-2111. | No reported prevalence of frailty |
| 161 | Corsonello A, Pedone C, Lattanzio F, et al. Chronic kidney disease and 1-year survival in elderly patients discharged from acute care hospitals: a comparison of three glomerular filtration rate equations[J]. Nephrology Dialysis Transplantation, 2010, 26(1): 360-364. | No reported prevalence of frailty |
| 162 | Ishikawa H, Hibino T, Moriyama Y. Chronic kidney disease is associated with physical impairment[J]. Journal of Rehabilitation Medicine – Clinical Communications, 2019, 2(1): 1000013. | No reported prevalence of frailty |
| 163 | Kosmadakis G, Correia E, Somda F, et al. Comparison of intradialytic hemodynamic tolerance between on-line hemodiafiltration and acetate-free biofiltration with profiled potassium dialysate concentration[J]. Saudi J Kidney Dis Transpl, 2017, 28(3): 558-565. | No reported prevalence of frailty |
| 164 | Kosmadakis G, Correia E D C, Albaret J, et al. Comparison of the hemodynamic tolerance and the biological parameters of four acetate-free hemodialysis methods[J]. Néphrologie & Thérapeutique, 2017, 13(7): 532-536. | No reported prevalence of frailty |
| 165 | Chen G, Zhang H, Du X, et al. Comparison of the prevalence and associated factors of cognitive frailty between elderly and middle-young patients receiving maintenance hemodialysis[J]. International Urology and Nephrology, 2022. | No reported prevalence of frailty |
| 166 | Susanto C, Kooman J, Courtens A M, et al. Conservative care as a treatment option for patients aged 75 years and older with CKD stage V: a National survey in the Netherlands[J]. European Geriatric Medicine, 2018, 9(2): 235-242. | No reported prevalence of frailty |
| 167 | Jhamb M, Argyropoulos C, Steel J L, et al. Correlates and Outcomes of Fatigue among Incident Dialysis Patients[J]. Clinical journal of the American Society of Nephrology, 2009, 4(11): 1779-1786. | No reported prevalence of frailty |
| 168 | Hsu C M, Weiner D E, Aweh G, et al. COVID-19 Among US Dialysis Patients: Risk Factors and Outcomes From a National Dialysis Provider[J]. Am J Kidney Dis, 2021, 77(5): 748-756. | No reported prevalence of frailty |
| 169 | Crespo M, Pérez-Sáez M J, Redondo-Pachón D, et al. COVID-19 in elderly kidney transplant recipients[J]. Am J Transplant, 2020, 20(10): 2883-2889. | No reported prevalence of frailty |
| 170 | Poppe E S J M, Polinder-Bos H A, Huberts M, et al. Creatinine synthesis rate and muscle strength and self-reported physical health in dialysis patients[J]. Clinical Nutrition, 2020, 39(5): 1600-1607. | No reported prevalence of frailty |
| 171 | Yanishi M, Tsukaguchi H, Kimura Y, et al. Evaluation of physical activity in sarcopenic conditions of kidney transplantation recipients[J]. International Urology and Nephrology, 2017, 49(10): 1779-1784. | No reported prevalence of frailty |
| 172 | Yao Y H, Chou Y J, Huang N. Facility size and mortality in hospital‐based and freestanding haemodialysis units: A nationwide retrospective cohort study[J]. Nephrology, 2019, 24(11): 1157-1164. | No reported prevalence of frailty |
| 173 | 286Tao X, Chow S, Zhang H, et al. Family caregiver's burden and the social support for older patients undergoing peritoneal dialysis[J]. J Ren Care, 2020, 46(4): 222-232. | No reported prevalence of frailty |
| 174 | Gobbi L, Baruzzo E, Iodice L, et al. Fatigue in hemodialysis patients: A single-center cross-sectional study[J]. Clinical Nephrology, 2021, 96(5): 281-288. | No reported prevalence of frailty |
| 175 | Feng S, Wolfe R A, Port F K. Frailty Survival Model Analysis of the National Deceased Donor Kidney Transplant Dataset Using Poisson Variance Structures[J]. Journal of the American Statistical Association, 2014, 100: 728-735. | No reported prevalence of frailty |
| 176 | 321Iqbal M S, Iqbal Q, Iqbal S, et al. Hemodialysis as long term treatment: Patients satisfaction and its impact on quality of life[J]. Pak J Med Sci, 2021, 37(2): 398-402. | No reported prevalence of frailty |
| 177 | Anderson B, Khalil K, Evison F, Nath J, Sharif A. Hypoalbuminaemia at time of surgery is associated with an increased risk for overall graft loss after kidney transplantation. Nephrology (Carlton)[J]. 2019;24(8):841-848. | No reported prevalence of frailty |
| 178 | Bohm C J, Storsley L J, Hiebert B M, et al. Impact of Exercise Counseling on Physical Function in Chronic Kidney Disease: An Observational Study[J]. Canadian Journal of Kidney Health and Disease, 2017, 5: 1063926911. | No reported prevalence of frailty |
| 179 | Ali H, Soliman K, Mohamed M M, et al. Impact of kidney transplantation on functional status[J]. Annals of Medicine, 2021, 53(1): 1303-1309. | No reported prevalence of frailty |
| 180 | Meier RPH, Noguchi H, Kelly YM, et al. Impact of Sarcopenia on Simultaneous Pancreas and Kidney Transplantation Outcomes: A Retrospective Observational Cohort Study. Transplant Direct[J]. 2020;6(10):e610. | No reported prevalence of frailty |
| 181 | Hall R K, Haines C, Gorbatkin S M, et al. Incorporating Geriatric Assessment into a Nephrology Clinic: Preliminary Data from Two Models of Care[J]. Journal of the American Geriatrics Society, 2016, 64(10): 2154-2158. | No reported prevalence of frailty |
| 182 | Bellin E Y, Hellebrand A M, Kaplan S M, et al. Post‐dialysis recovery time in ESRD patients receiving more frequent hemodialysis in skilled nursing facilities[J]. Hemodialysis International, 2022. | No reported prevalence of frailty |
| 183 | Manay P, Ten Eyck P, Siniff E, et al. Psychosocial characteristics of patients evaluated for kidney transplant and associations with functional and frailty metrics at a veterans affairs hospital. Clin Transplant. 2022;36(2):e14530. | No reported prevalence of frailty |
| 184 | Marino C, Angelici L, Pistolesi V, et al. SARS-CoV-2 Infection in Patients on Dialysis: Incidence and Outcomes in the Lazio Region, Italy[J]. Journal of Clinical Medicine, 2021, 10(24): 5818. | No reported prevalence of frailty |
| 185 | Gamboa J L, Roshanravan B, Towse T, et al. Skeletal Muscle Mitochondrial Dysfunction Is Present in Patients with CKD before Initiation of Maintenance Hemodialysis[J]. Clin J Am Soc Nephrol, 2020, 15(7): 926-936. | No reported prevalence of frailty |
| 186 | Li C, Chen L, He L, et al. Study on the relationship between sarcopenia and its components and anorexia in elderly maintenance haemodialysis patients. Nurs Open[J]. 2022;9(2):1096-1104. | No reported prevalence of frailty |
| 187 | Ch'Ng C C, Ong L M, Beh K K M, et al. Survival advantage of initiating dialysis in elderly and non‐elderly incident end‐stage kidney disease patients[J]. Nephrology, 2020, 25(8): 644-651. | No reported prevalence of frailty |
| 188 | Chen J H C, Brown M A, Jose M, et al. Temporal changes and risk factors for death from early withdrawal within 12 months of dialysis initiation—a cohort study[J]. Nephrology Dialysis Transplantation, 2022, 37(4): 760-769. | No reported prevalence of frailty |
| 189 | Kosoku A, Ishihara T, Iwai T, et al. The Change in Muscle Mass Among Kidney Transplant Recipients: A Prospective Cohort Study[J]. Transplantation Proceedings, 2022, 54(2): 346-350. | No reported prevalence of frailty |

Table S5 The list of studies that were excluded from the meta-analysis due to data overlapping

| NO. | Title | Reasons |
| --- | --- | --- |
|  | ACTIVE/ADIPOSE cohort (Overlap with the study of Johansen KL et al. [1]) |  |
| 1 | Johansen KL, Dalrymple LS, Delgado C, et al. Association between body composition and frailty among prevalent hemodialysis patients: a US Renal Data System special study. J Am Soc Nephrol. 2014 Feb;25(2):381-9. doi: 10.1681/ASN.2013040431. | Overlap |
| 2 | Johansen KL, Dalrymple LS, Glidden D, et al. Association of Performance-Based and Self-Reported Function-Based Definitions of Frailty with Mortality among Patients Receiving Hemodialysis. Clin J Am Soc Nephrol. 2016 Apr 7;11(4):626-32. doi: 10.2215/CJN.03710415. | Overlap |
| 3 | Sy J, McCulloch CE, Johansen KL. Depressive symptoms, frailty, and mortality among dialysis patients. Hemodial Int. 2019 Apr;23(2):239-246. doi: 10.1111/hdi.12747. | Overlap |
| 4 | Chiang JM, Kaysen GA, Segal M, et al. Low testosterone is associated with frailty, muscle wasting and physical dysfunction among men receiving hemodialysis: a longitudinal analysis. Nephrol Dial Transplant. 2019 May 1;34(5):802-810. doi: 10.1093/ndt/gfy252. | Overlap |
| 5 | Kimura H, Kalantar-Zadeh K, Rhee CM, et al. Polypharmacy and Frailty among Hemodialysis Patients. Nephron. 2021;145(6):624-632. doi: 10.1159/000516532. | Overlap |
| 6 | Kutner NG, Zhang R, Huang Y, et al. Risk factors for frailty in a large prevalent cohort of hemodialysis patients. Am J Med Sci. 2014 Oct;348(4):277-82. doi: 10.1097/MAJ.0000000000000250. | Overlap |
| 7 | Johansen KL, Delgado C, Kaysen GA, et al. Frailty Among Patients Receiving Hemodialysis: Evolution of Components and Associations with Mortality. J Gerontol A Biol Sci Med Sci. 2019 Feb 15;74(3):380-386. doi: 10.1093/gerona/gly206. | Overlap^#^ |
| 8 | Sy J, Streja E, Grimes B, et al. The Marginal Cost of Frailty Among Medicare Patients on Hemodialysis. Kidney Int Rep. 2019 Dec 6;5(3):289-295. doi: 10.1016/j.ekir.2019.11.020. | Overlap |
| 9 | Johansen KL, Painter P, Delgado C, et al. Characterization of physical activity and sitting time among patients on hemodialysis using a new physical activity instrument. J Ren Nutr. 2015 Jan;25(1):25-30. doi: 10.1053/j.jrn.2014.06.012. | Overlap |
| 10 | Johansen KL, Dalrymple LS, Delgado C, et al. Comparison of self-report-based and physical performance-based frailty definitions among patients receiving maintenance hemodialysis. Am J Kidney Dis. 2014 Oct;64(4):600-7. doi: 10.1053/j.ajkd.2014.03.016. | Overlap |
| 11 | Kutner NG, Zhang R, Allman RM, et al. Correlates of ADL difficulty in a large hemodialysis cohort. Hemodial Int. 2014 Jan;18(1):70-7. doi: 10.1111/hdi.12098. | Overlap |
|  | CanFIT cohort (Overlap with the study of Brar RS et al. [2]) |  |
| 12 | Walker SR, Brar R, Eng F, et al. Frailty and physical function in chronic kidney disease: the CanFIT study. Can J Kidney Health Dis. 2015 Sep 5; 2: 32. doi: 10.1186/s40697-015-0067-4. | Overlap |
|  | LCDP cohort (Overlap with the study of Chao CT et al. [3]) |  |
| 13 | Lee SY, Wang J, Chao CT, et al. Frailty modifies the association between opioid use and mortality in chronic kidney disease patients with diabetes: a population-based cohort study. Aging (Albany NY). 2020 Nov 7;12(21):21730-21746. doi: 10.18632/aging.103978. | Overlap |
| 14 | Chao CT, Wang J, Huang JW, et al. Chronic kidney disease-related osteoporosis is associated with incident frailty among patients with diabetic kidney disease: a propensity score-matched cohort study. Osteoporos Int. 2020 Apr;31(4):699-708. doi: 10.1007/s00198-020-05353-9. | Overlap |
| 15 | Chao CT, Huang JW; COGENT (COhort of GEriatric Nephrology in NTUH) study group. Geriatric syndromes are potential determinants of the medication adherence status in prevalent dialysis patients. PeerJ. 2016 Jun 14;4: e2122. doi: 10.7717/peerj.2122. | Overlap |
| 16 | Chao CT, Hsu YH, Chang PY, et al. Simple self-report FRAIL scale might be more closely associated with dialysis complications than other frailty screening instruments in rural chronic dialysis patients. Nephrology (Carlton). 2015 May;20(5):321-8. doi: 10.1111/nep.12401. | Overlap |
| 17 | Chao CT, Lai HJ, Tsai HB, et al. Frail phenotype is associated with distinct quantitative electroencephalographic findings among end-stage renal disease patients: an observational study. BMC Geriatr. 2017 Dec 2;17(1):277. doi: 10.1186/s12877-017-0673-3. | Overlap |
| 18 | Lee SY, Wang J, Chao CT, et al. Frailty is associated with a higher risk of developing delirium and cognitive impairment among patients with diabetic kidney disease: A longitudinal population-based cohort study. Diabet Med. 2021 Jul;38(7): e14566. doi: 10.1111/dme.14566. | Overlap |
| 19 | Lee SY, Wang J, Tsai HB, et al. Muscle relaxant use and the associated risk of incident frailty in patients with diabetic kidney disease: a longitudinal cohort study. Ther Adv Drug Saf. 2021 Jun 11; 12: 20420986211014639. doi: 10.1177/20420986211014639. | Overlap |
| 20 | Chao CT, Huang JW, Chan DC, et al. Frail phenotype might herald bone health worsening among end-stage renal disease patients. PeerJ. 2017 Jul 10;5: e3542. doi: 10.7717/peerj.3542. | Overlap |
|  | CRIC cohort (Overlap with the study of Slaven A et al. [4]) |  |
| 21 | Ghazi L, Yaffe K, Tamura MK, et al. Association of 24-Hour Ambulatory Blood Pressure Patterns with Cognitive Function and Physical Functioning in CKD. Clin J Am Soc Nephrol. 2020 Apr 7;15(4):455-464. doi: 10.2215/CJN.10570919. | Overlap |
| 22 | Reese PP, Cappola AR, Shults J, et al. Physical performance and frailty in chronic kidney disease. Am J Nephrol. 2013;38(4):307-15. doi: 10.1159/000355568. | Overlap |
|  | China cohort (Overlap with the study of Chan et al. [5]) |  |
| 23 | Chan GC, Ng JK, Chow KM, et al. Depression does not predict clinical outcome of Chinese peritoneal Dialysis patients after adjusting for the degree of frailty. BMC Nephrol. 2020 Aug 5;21(1):329. doi: 10.1186/s12882-020-01994-4. | Overlap |
| 24 | Chan GC, N G JK, Chow KM, et al. Interaction between central obesity and frailty on the clinical outcome of peritoneal dialysis patients. PLoS One. 2020 Oct 26;15(10): e0241242. doi: 10.1371/journal.pone.0241242. | Overlap |
|  | Baltimore cohort (Overlap with the study of Haugen CE et al. [6], Chu NM et al. [7], and Chen X et al. [8]) |  |
| 25 | McAdams-DeMarco MA, Isaacs K, Darko L, et al. Changes in Frailty After Kidney Transplantation. J Am Geriatr Soc. 2015 Oct;63(10):2152-7. doi: 10.1111/jgs.13657. | Overlap |
| 26 | McAdams-DeMarco MA, Law A, Salter ML, et al. Frailty and early hospital readmission after kidney transplantation. Am J Transplant. 2013 Aug;13(8):2091-5. doi: 10.1111/ajt.12300. | Overlap |
| 27 | McAdams-DeMarco MA, Suresh S, Law A, et al. Frailty and falls among adult patients undergoing chronic hemodialysis: a prospective cohort study. BMC Nephrol. 2013 Oct 16; 14: 224. doi: 10.1186/1471-2369-14-224. | Overlap |
| 28 | McAdams-DeMarco MA, Ying H, Olorundare I, et al. Individual Frailty Components and Mortality in Kidney Transplant Recipients. Transplantation. 2017 Sep;101(9):2126-2132. doi: 10.1097/TP.0000000000001546. | Overlap |
| 29 | Chu NM, Ruck J, Chen X, et al. Long-term Trajectories of Frailty and its Components after Kidney Transplantation. J Gerontol A Biol Sci Med Sci. 2022 Feb 20: glac051. doi: 10.1093/gerona/glac051. | Overlap |
| 30 | Chu NM, Gross AL, Shaffer AA, et al. Frailty and Changes in Cognitive Function after Kidney Transplantation. J Am Soc Nephrol. 2019 Feb;30(2):336-345. doi: 10.1681/ASN.2018070726. | Overlap |
| 31 | McAdams-DeMarco MA, Law A, King E, et al. Frailty and mortality in kidney transplant recipients. Am J Transplant. 2015 Jan;15(1):149-54. doi: 10.1111/ajt.12992. | Overlap^#^ |
| 32 | McAdams-DeMarco MA, Law A, Salter ML, et al. Frailty as a novel predictor of mortality and hospitalization in individuals of all ages undergoing hemodialysis. J Am Geriatr Soc. 2013 Jun;61(6):896-901. doi: 10.1111/jgs.12266. | Overlap^#^ |
| 33 | Chu NM, Shi Z, Berkowitz R, et al. Poor Outcomes in Kidney Transplant Candidates and Recipients with History of Falls. Transplantation. 2020 Aug;104(8):1738-1745. doi: 10.1097/TP.0000000000003057. | Overlap |
| 34 | Garonzik-Wang JM, Govindan P, Grinnan JW, et al. Frailty and delayed graft function in kidney transplant recipients. Arch Surg. 2012 Feb;147(2):190-3. doi: 10.1001/archsurg.2011.1229. | Overlap |
| 35 | McAdams-DeMarco MA, Law A, Tan J, et al. Frailty, mycophenolate reduction, and graft loss in kidney transplant recipients. Transplantation. 2015 Apr;99(4):805-10. doi: 10.1097/TP.0000000000000444. | Overlap |
| 36 | Nastasi AJ, McAdams-DeMarco MA, Schrack J, et al. Pre-Kidney Transplant Lower Extremity Impairment and Post-Kidney Transplant Mortality. Am J Transplant. 2018 Jan;18(1):189-196. doi: 10.1111/ajt.14430. | Overlap |
| 37 | Thomas AG, Ruck JM, Shaffer AA, et al. Kidney Transplant Outcomes in Recipients with Cognitive Impairment: A National Registry and Prospective Cohort Study. Transplantation. 2019 Jul;103(7):1504-1513. doi: 10.1097/TP.0000000000002431. | Overlap |
| 38 | Haugen CE, Agoons D, Chu NM,et al. Physical Impairment and Access to Kidney Transplantation. Transplantation. 2020 Feb;104(2):367-373. doi: 10.1097/TP.0000000000002778. | Overlap |
| 39 | Haugen CE, Gross A, Chu NM, et al. Development and Validation of an Inflammatory-Frailty Index for Kidney Transplantation. J Gerontol A Biol Sci Med Sci. 2021 Feb 25;76(3):470-477. doi: 10.1093/gerona/glaa167. | Overlap |
|  | GOLD cohort (Overlap with the study of van Loon IN et al. [9]) |  |
| 40 | Goto NA, van Loon IN, Boereboom FTJ, et al. Association of Initiation of Maintenance Dialysis with Functional Status and Caregiver Burden. Clin J Am Soc Nephrol. 2019 Jul 5;14(7):1039-1047. doi: 10.2215/CJN.13131118. | Overlap |
| 41 | Goto NA, van Loon IN, Morpey MI, et al. Geriatric Assessment in Elderly Patients with End-Stage Kidney Disease. Nephron. 2019;141(1):41-48. doi: 10.1159/000494222. | Overlap |
|  | Korea cohort (Overlap with the study of Lee SY et al. [10]) |  |
| 42 | Kang SH, Do JY, Kim JC. The relationship between disability and clinical outcomes in maintenance dialysis patients. Yeungnam Univ J Med. 2021 Apr;38(2):127-135. doi: 10.12701/yujm.2020.00346. | Overlap |
| 43 | Kang SH, Do JY, Lee SY, et al. Effect of dialysis modality on frailty phenotype, disability, and health-related quality of life in maintenance dialysis patients. PLoS One. 2017 May 3;12(5): e0176814. doi: 10.1371/journal.pone.0176814. | Overlap |
| 44 | Kang SH, Do JY, Jeong HY, et al. The Clinical Significance of Physical Activity in Maintenance Dialysis Patients. Kidney Blood Press Res. 2017;42(3):575-586. doi: 10.1159/000480674. | Overlap |
|  | UK cohort (Overlap with the study of Zanotto T et al. [11]) |  |
| 45 | Zanotto T, Mercer TH, van der Linden ML, et al. The relative importance of frailty, physical and cardiovascular function as exercise-modifiable predictors of falls in haemodialysis patients: a prospective cohort study. BMC Nephrol. 2020 Mar 14;21(1):99. doi: 10.1186/s12882-020-01759-z. | Overlap |
|  | Spain cohort (Overlap with the study of Pérez-Sáez MJ et al. [12]) |  |
| 46 | Pérez-Sáez MJ, Arias-Cabrales CE, Dávalos-Yerovi V, et al. Frailty among chronic kidney disease patients on the kidney transplant waiting list: the sex-frailty paradox. Clin Kidney J. 2021 Jul 10;15(1):109-118. doi: 10.1093/ckj/sfab133. | Overlap |
| 47 | Pérez-Sáez MJ, Dávalos-Yerovi V, Redondo-Pachón D, et al. Frailty in kidney transplant candidates: a comparison between physical frailty phenotype and FRAIL scales. J Nephrol. 2022 Jan 3. doi: 10.1007/s40620-021-01234-4. | Overlap |
|  | A secondary analysis of the Frailty Assessment in Chronic Kidney Disease study (Overlap with the study of Nixon AC et al. [13]) |  |
| 48 | Nixon AC, Bampouras TM, Pendleton N, et al. Frailty is independently associated with worse health-related quality of life in chronic kidney disease: a secondary analysis of the Frailty Assessment in Chronic Kidney Disease study. Clin Kidney J. 2019 Apr 30;13(1):85-94. doi: 10.1093/ckj/sfz038. | Overlap |

#This study was excluded from the meta-analysis of frailty prevalence but included the meta-analysis of frailty and mortality.

Table S6 Characteristic of included 187 articles

| Author | Study design | Region | Age (y, mean±SD) ^①^ | Male | BMI (kg/m^2^, mean±SD) ^①^ | CKD stage | Total | Prefrail | Frailty | Tool | Death | Follow up | Adjusted |
| --- | --- | --- | --- | --- | --- | --- | --- | --- | --- | --- | --- | --- | --- |
| 139 included articles | | | | | | | | | | | | | |
| Roshanravan B [14] | Cohort study | North America (USA) | 59±13 | 0.81 | 31.4±7.4 | Predialysis (stage 1-4) | 336 | 175 | 47 | Fried phenotype | 30 | 987 d | Age, sex, BMI, eGFR, diabetes and cardiovascular disease. |
| Davenport A [15] | Cross-sectional study | Europe (UK) | 64.6±16.6 | 0.602 | 25.7±6.0 | HD | 2,089 | — | 890 | CFS | — | — | — |
| Ali H [16] | Cohort study | Europe (UK) | 78.2±20.3 † | 0.51 | Not reported | Predialysis | 103 | — | 58 | PRISMA | 17 | 20 months | Calcium, hemoglobin, baseline eGFR, Karnofsky grade, ethnicity, age, sex, average albumin, Charlson comorbidity score. |
| Orlandi FD [17] | Cross-sectional study | South America (Brazil) | 71.1±6.8 | 0.7 | Not reported | HD | 60 | 28 | 11 | EFS | — | — | — |
| Kosaka S [18] | Cross-sectional study | Asia (Japan) | 72.1±9.8 † | 0.661 | 23.8±3.9 | Predialysis (stage 3-5) | 109 | — | 31 | Fried phenotype | — | — | — |
| Yoneki K [19] | Cross-sectional study | Asia (Japan) | 67.2±7.5 † | 0.58 | 20.7±3.7 † | HD | 214 | 84 | 64 | Fried phenotype | — | — | — |
| Santos DGMD [20] | Cross-sectional study | South America (Brazil) | 60.0 | 0.45 | Not reported | HD | 80 | 17 | 59 | SFA | — | — | — |
| Usui N [21] | Cross-sectional study | Asia (Japan) | 74.1±6.8 | 0.72 | 22.2±3.2 | HD | 158 | — | 73 | Fried phenotype | — | — | — |
| Yabuuchi J [22] | Cross-sectional study | Asia (Japan) | 67.2 | 0.22 | Not reported | HD | 37 | 24 | 8 | Fried phenotype | — | — | — |
| Delgado C [23] | Cohort study | North America (USA) | 51.6±14.1 † | 0.605 | Not reported | Predialysis | 812 | 430 | 130 | Fried phenotype | 371 | 17 y | Urine protein, sex, age, race, BMI and albumin quartiles and group randomization by diet, blood pressure assignment, and eGFR. |
| Delgado C [24] | Cross-sectional study | North America (USA) | 55±13 | 0.63 | Not reported | HD | 80 | — | 47 | Fried phenotype | — | — | — |
| Mansur HN [25] | Longitudinal studies | North America (Brazil) | 60.5±11.5 | 0.59 | Not reported | Predialysis (stage 3-5) | 61 | — | 26 | Fried phenotype | — | — | — |
| Luo CM [26] | Cohort study | Asia (China) | 65±13 | 0.54 | Not reported | HD | 761 | 269 | 236 | Fried phenotype | — | — | — |
| Delgado C [27] | Cohort study | North America (USA) | 62.6±15.6 † | 0.55 | 29.6±8.2 | HD | 1,053 | — | 808 | Fried phenotype | — | — | — |
| Smith G [28] | Cross-sectional study | Europe (UK) | 74±7.1 | 0.71 | 28.6±4.6 | Predialysis (stage 4-5) | 276 | 148 | 86 | Fried phenotype | — | — | — |
| Zhang R [29] | Cross-sectional study | Asia (China) | 62.08±14.04 | 0.48 | Not reported | PD | 170 | 73 | 54 | Clinical Frailty Scale | — | — | — |
| Lysak N [30] | Cohort study | North America (USA) | 74.6±6.9 † | 0.59 | Not reported | Predialysis | 2,465 | 781 | 1,390 | Scores based on ICD-9 codes | — | — | — |
| Kim JC [31] | Cross-sectional study | Asia (Korea) | 56.5±11.9 † | 0.53 | 23.7±3.6 † | HD | 83 | — | 24 | Fried phenotype | — | — | — |
| Mitra S [32] | Cohort study | Europe | 64±15 | 0.61 | 27±5 | KTR | 1,423 | — | — | CFS | 28 | 28 d | Age, tobacco use, autoimmune disease, shortness of breath. |
| Fernandez MP [33] | Cohort study | North America (USA) | 53.8±13.5 | 0.599 | Category | Mixed | 2,086 | 1,312 | 378 | Fried phenotype | — | — | — |
| Davenport A [34] | Cross-sectional study | Europe (UK) | 60.9±16.13 | 0.61 | 26.2±5.1 | PD | 368 | — | 71 | CFS | — | — | — |
| Kim JC [35] | Cross-sectional study | Asia (Korea) | 56.4±13.2 | 0.565 | 22.1±3.2 | HD | 1,247 | — | 421 | Fried phenotype | — | — | — |
|  | Cross-sectional study | Asia (Korea) | 54.1±11.9 | 0.536 | 23.5±3.1 | PD | 364 | — | 136 | Fried phenotype | — | — | — |
| Szeto CC [36] | Cohort study | Asia (China) | 60.8±11.8 | 0.57 | Not report | PD | 178 | — | 111 | Chinese Frailty Score | — | — | — |
| Konel JM [37] | Cohort study | North America (USA) | 54±14 | 0.622 | Not reported | KTR | 773 | — | 126 | Fried phenotype | Not reported | 5 years |  |
| Nixon AC [13] | Cross-sectional study | Europe (UK) | 69±13 | 0.5 | 28.8±6.0 † | Mixed | 90 | 42 | 19 | Fried phenotype | — | — | — |
| van Munster BC [38] | Cross-sectional study | Europe (The Netherlands) | 65.2±12 | 0.568 | 27.0±4.0 † | Mixed | 95 | — | 35 | FI | — | — | — |
| Van Pilsum, Rasmussen S [39] | Cross-sectional study | North America (USA) | Category | 0.613 | Not reported | Mixed | 460 | — | 95 | Fried phenotype | — | — | — |
| Johansen KL [1] | Cohort study | North America (USA) | 57.2±14.2 | 0.593 | 28.2±6.9 | HD | 762 | 427 | 240 | Fried phenotype | — | — | — |
| Hubbard RE [40] | Cross-sectional study | Oceania (Australia) | 65.2±14.6 | 0.536 | Not reported | Predialysis | 110 | — | 21 | Fried phenotype | — | — | — |
| Bancu I [41] | Cross-sectional study | Europe (Spain) | 70.26±13.85 | 0.594 | 25.93±5.18 | HD | 320 | — | 18 | FRAIL Scale | — | — | — |
| Wilhelm-Leen ER [42] | Cohort study | North America (USA) | Not reported | Not reported | Not reported | Mixed (CKD 1-5) | 1,289 | — | 103 | Fried phenotype | — | — | — |
| McAdams-DeMarco MA [43] | Cohort study | North America (USA) | 54.8±13.3 | 0.565 | 29.7±8.3 † | HD | 324 | 122 | 110 | Fried phenotype | — | — | — |
| Pugh J [44] | Cohort study | Europe (UK) | 72.6±13.4 † | 0.56 | 28.2±6.6 † | Predialysis | 283 | 131 | 51 | CFS | 117 | 3 y | Age, gender and eGFR. |
| McAdams-DeMarco MA [45] | Longitudinal study | North America (USA) | 53.3±14.0 | 0.579 | 28.2±5.3 † | Mixed | 233 | 62 | 55 | Fried phenotype | — | — | — |
| Alfaadhel TA [46] | Cohort study | North America (Canada) | 63±15 | 0.67 | 29.4±6.7 † | Dialysis | 390 | — | 205 | CFS | 96 | 1.7 y | Age, race, sex, Charlson Comorbidity Index, diabetic ESRD, eGFR, albumin, dialysis modality, and location of dialysis start. |
| Mansur HN [47] | Cross-sectional study | South America (Brazil) | 60.5±11.5 | 0.59 | 25.9±5.0 | Predialysis | 61 | — | 26 | Fried phenotype | — | — | — |
| Noori N [48] | Cross-sectional study | North America (Canada) | 64±14 | 0.56 | 27.0±5.4 | HD | 151 | — | 74 | Fried phenotype | — | — | — |
| SchopmeyerL [49] | Cross-sectional study | Europe (The Netherlands) | 51.81±14.5 | 0.626 | 25.9±4.7 † | KTR | 139 | — | 23 | GFI | — | — | — |
| Ng JK [50] | Cohort study | Asia (China) | 60.6±12.1 | 0.5 | 24.9±4.7 † | PD | 193 | — | 134 | Chinese Frailty Score | 60 | 2 years | Not reported |
| Poveda V [51] | Cross-sectional study | Europe (Portugal) | 64.3±14.6 | 0.53 | 25.7±4.4 † | HD | 83 | 45 | 24 | FRAIL Scale | — | — | — |
| Clark DA [52] | Cross-sectional study | North America (Canada) | 61±14 | 0.58 | 28.4±5.3 † | Dialysis | 98 | — | 32 | CFS | — | — | — |
| Guo Y [53] | Cohort study | Asia (China) | 71.65±5.89 | 0.554 | 23.95±4.28 | HD | 204 | 41 | 147 | Fried phenotype | — | 52 weeks | Age, history of diabetes, MoCA<26, single-pool Kt/V, and albumin and iPTH. |
| Vettoretti S [54] | Cross-sectional study | Europe (Italy) | 80±6 | 0.7 | 28±4.8 | Predialysis (CKD 3b-5) | 112 | — | 50 | Fried phenotype | — | — | — |
| Bloomfield K [55] | Cohort study | Oceania (New Zealand) | 61.5±13.5 | 0.493 | Not reported | HD | 138 | 62 | 51 | Fried phenotype | Prefrail: 18  Frail: 51 | 3 years | Age, gender, ethnicity, marital status, smoking status, aetiology of renal disease, albumin, haemoglobin, Kt/V, natural logarithm transformed months on dialysis. |
|  | Cohort study | Oceania (New Zealand) | 61.5±13.5 | 0.493 | Not reported | HD | 138 | 48 | 51 | EFS | Prefrail: 48  Frail: 51 | 3 years | Age, gender, ethnicity, marital status, smoking status, aetiology of renal disease, albumin, haemoglobin, Kt/V log(months on dialysis). |
| Clark D [56] | Cohort study | North America (Canada) | 62±15 | 0.631 | 29±7 | Dialysis | 564 | 235 | 79 | CFS | 220 | 2.4 years | Cause of end stage kidney disease, age, sex, race, early nephrology referral, comorbidities (cancer, coronary artery disease, congestive heart failure, cerebrovascular disease, pulmonary disease, liver disease, diabetes ), and albumin |
| Moreno-Useche LD [57] | Cross-sectional study | South America (Colombia) | 63.9±8.3 † | 0.636 | Not reported | Dialysis | 66 | — | 36 | FRAIL Scale | — | — | — |
| Fitzpatrick J [58] | Cohort study | North America (USA) | 54.9±13.1 | 0.58 | 29.3±7.9 | HD | 370 | — | 193 | Fried phenotype | 81 | 2.48 years | Age, sex, race, Charlson Comorbidity Index and albumin. |
| Tsai MD [59] | Cross-sectional study | Asia (China) | 66.8±9.1 | 0.729 | Not reported | Predialysis (CKD 3b-5) | 144 | — | 54 | SOF | — | — | — |
| Adame Perez SI [60] | Cross-sectional study | North America (Canada) | 69.6±6.9 † | 0.63 | 31±5.5 | Predialysis | 41 | — | 7 | EFS | — | — | — |
| McAdams-DeMarco MA [61] | Cohort study | North America (USA) | 53.7±13.5 | 0.595 | 29.2±5.9 † | Dialysis | 1,975 | 1,239 | 363 | Fried phenotype | 27 | 1.1 years | Age, race, sex, blood type, and cause of ESRD. |
| Lopez-Montes A [62] | Cohort study | Europe (Spain) | 78.1±4.1 | 0.632 | 26.4±6.0 | HD | 117 | — | 63 | Fried phenotype | 117 | 1 year | Age, sex, Charlson index and BMI. |
| Hornik B [63] | Cross-sectional study | Europe (Poland) | 57.8±16.0 | 0.5 | 24.5±4.5 | HD | 72 | 11 | 29 | CSHA-CFS | — | — | — |
| Meulendijks FG [64] | Cohort study | Europe (The Netherlands) | 77.8±19.7 † | 0.65 | 27.3±16.7 † | Predialysis | 63 | — | 20 | GFI | — | — | — |
| Drost D [65] | Cross-sectional study | Europe (The Netherlands) | 65.2±12.0 | 0.43 | 27.0±4.0 † | Mixed | 95 | — | 35 | FI | — | — | — |
|  |  |  |  |  |  |  | 88 | 49 | 24 | Fried phenotype |  |  |  |
| Weng SC [66] | Cohort study | Asia (China) | 81.3±6.6 | 0.7 | 24.7±4.0 † | Predialysis | 331 | 68 | 246 | Rockwood frailty index | 44 | 3.1 | Age and gender. |
| Lee SJ [67] | Cross-sectional study | Asia (Korea) | 65.9±13.9 † | 0.63 | Not reported | Stage 2-4 | 168 | — | 63 | Fried phenotype | — | — | — |
| Lee SW [68] | Cohort study | Asia (Korea) | 71.9±6.5 † | 0.37 | 22.2±3.7 † | Predialysis (stage 5) | 46 | — | 15 | Multidimensional frailty score | — | — | — |
| Warsame F [69] | Cohort study | North America (USA) | Category | 0.729 | 29.9±6.7 | Mixed | 140 | 107 | 23 | Fried phenotype | — | — | — |
| Guo F [70] | Cross-sectional study | Asia (China) | 31±7.63 | 0.51 | NP | HD | 196 | — | 92 | Fried phenotype | — | — | — |
| Polinder-Bos HA [71] | Cohort study | Europe (The Netherlands) | 63.1±3.5 † | 0.63 | Not reported | Dialysis | 353 | — | 198 | Fried phenotype | — | — | — |
| Mentias A [72] | Cohort study | North America (USA) | 65.6±6.9 † | 0.65 | Not reported | HD | 6,695 | — | 2,846 | Frailty Score | — | — | — |
| Hernandez-Agudelo SY [73] | Cross-sectional study | South America (Colombia) | 58.3±16.0 † | 0.49 | Not reported | HD | 82 | 29 | 24 | CFS | — | — | — |
| Pérez-Sáez MJ [12] | Cohort study | Europe (Spain) | 60.4±14.1 | 0.686 | 27.9±5.2 | Mixed | 449 | 273 | 47 | Fried phenotype | 34 | 26 months | Age, sex, race, hypertension, diabetes mellitus, any cardiovascular disease, chronic obstructive pulmonary disease, dialysis vintage. |
|  |  |  |  |  |  |  |  | 186 | 16 | FRAIL Scale |  |  |  |
| Farragher JF [74] | Cross-sectional study | North America (Canada) | 69.2±10.1 | 0.67 | Not reported | PD | 110 | — | 71 | Fried phenotype | — | — | — |
| Salter ML [75] | Cross-sectional study | North America (USA) | 61.1±3.4 † | 0.534 | 26.9±2.1 † | HD | 110 | 31 | 30 | Fried phenotype | — | — | — |
| Okuyama M [76] | Cross-sectional study | Asia (Japan) | 67.4±11.8 † | 0.61 | 21.9±3.5 † | HD | 362 | — | 75 | Fried phenotype | — | — | — |
| Meyer AM [77] | Cohort study | Europe (Germany) | 78±9 | 0.645 | IQR25.7(7) | Mixed | 375 | 210 | 79 | CGA | — | — | — |
| Chan GCK [5] | Longitudinal study | Asia (China) | Not reported | Not reported | Not reported | PD | 267 | 74 | 120 | Chinese Frailty Score | — | — | — |
| Iyasere OU [78] | Cross-sectional study | Europe (UK) | 76.0±2.1 † | 0.589 | Not reported | PD | 129 | — | 67 | CFS | — | — | — |
|  | Cross-sectional study | Europe (UK) | 75.0±2.1 † | 0.598 | Not reported | HD | 122 | — | 52 | CFS | — | — | — |
| Candemir B [79] | Cross-sectional study | Asia (Turkey) | 72.2±2.4 † | 0.541 | 27.3±1.3 † | Mixed | 109 | — | 46 | Fried phenotype | — | — | — |
| Kamijo Y [80] | Cross-sectional study | Asia (Japan) | 66.8±13.2 | 0.706 | Not reported | PD | 119 | — | 10 | CFS | — | — | — |
| Johansen KL [81] | Cohort study | North America (USA) | 58.2±15.5 | 0.534 | 25.8±5.8 | Dialysis | 2,275 | — | 1,540 | Fried phenotype | Not reported | 1 year | Age, gender, race or ethnicity, body size, dialysis modality, comorbidities, albumin. |
| Slaven A [4] | Cross-sectional study | North America (USA) | 71.7±4.76 | 0.575 | 31.7±6.67 | Mixed | 1,709 | 1,147 | 495 | Fried phenotype | — | — | — |
| Wang J [82] | Cohort study | Asia (China) | 55.6±13.5 | 0.48 | 20.7±2.7 † | HD | 185 | — | 72 | TFI | — | — | — |
| Inoue T [83] | Cross-sectional study | Asia (Japan) | 78.0±2.3 † | 0.5 | 24.6±1.0 † | Stage 1-3 | 630 | 198 | 254 | Kihon Checklist | — | — | — |
| Zachciał J [84] | Cross-sectional study | Europe (Poland) | 61.65±12.11 | 0.53 | Not reported | KTR | 190 | — | 110 | TFI | — | — | — |
| Mutevelić-Turković A [85] | Cross-sectional study | Europe (Bosnia and Herzegovina) | Category | 0.601 | Not reported | HD | 281 | 58 | 126 | Fried phenotype | — | — | — |
| Li Y [86] | Cohort study | Asia (China) | 69.4±8.2 † | 0.497 | 23.1±3.8 † | HD | 150 | — | 52 | FRAIL Scale | 15 | 1 y | Age, gender, albumin, mini-nutrition assessment short form, medical history of diabetes, coronary heart disease, urea reduction rate. |
| Chen CH [87] | Cohort study | Asia (China) | 66.13±12.47 | 0.575 | 23.81±4.17 | HD | 313 | 92 | 126 | Fried phenotype | — | — | — |
| Takeuchi H [88] | Cross-sectional study | Asia (Japan) | 67.2±11.9 | 0.624 | 21.8±3.6 † | HD | 388 | 204 | 83 | Fried phenotype | — | — | — |
| Gopinathan JC [89] | Cross-sectional study | Asia (India) | 78.03±3.9 | 0.795 | Not reported | HD | 39 | — | 22 | Fried phenotype | — | — | — |
| Lee SY [10] | Cohort study | Asia (Korea) | 55.9±12.9 | 0.557 | 22.4±3.2 | Dialysis | 1,658 | 757 | 577 | Fried phenotype | 87 | 17.1 m | Age, sex, comorbidities, dialysis modality, disability, serum albumin and creatinine, and the other factors. |
| Jegatheswaran J [90] | Cohort study | North America (Canada) | 63.3±15.6 | 0.63 | Not reported | Dialysis | 261 | 145 | 77 | FRAIL Scale | — | — | — |
| Lorenz EC [91] | Cohort study | North America (USA) | 61.8±9.3 | 0.621 | 30.2±5.7 | KTC (dialysis) | 272 | — | 39 | Fried phenotype | 7 | 12.4 months | Age |
| Miller LM [92] | Cross-sectional study | North America (USA) | 73.2±9.0 † | 0.6 | 29.5±5.9 † | Predialysis (stage 3-4) | 2,253 | 1,196 | 806 | FI | — | — | — |
| Saitoh M [93] | Cross-sectional study | Asia (Japan) | 64±12 | 0.65 | 23.8±4.6 † | HD | 116 | — | 36 | Fried phenotype | — | — | — |
| Hendra H [94] | Cross-sectional study | Europe (UK) | 60.2±16.5 | 0.66 | 25.2±5.8 | HD | 172 | — | 54 | CFS | — | — | — |
| Davenport A [95] | Cross-sectional study | Europe (UK) | 65±12.5 | 0.727 | 26.1±4.3 † | HD | 22 | — | 12 | CFS | — | — | — |
| Imamura K [96] | Cohort study | Asia (Japan) | 68.1±11.9 | 0.61 | 22.1±4.1 | HD | 315 | — | 76 | Fried phenotype | — | — | — |
|  |  |  |  |  |  |  |  |  | 46 | SOF |  |  |  |
|  |  |  |  |  |  |  |  |  | 92 | SPPB |  |  |  |
|  |  |  |  |  |  |  |  |  | 106 | FI |  |  |  |
|  |  |  |  |  |  |  |  |  | 87 | FRAIL Scale |  |  |  |
|  |  |  |  |  |  |  |  |  | 56 | CFS |  |  |  |
| Anderson BM [97] | Cross-sectional study | Europe (UK) | 63.4±15.6 † | 0.586 | 27.5±6.8 † | HD | 485 | 219 | 203 | Fried phenotype | — | — | — |
|  |  |  |  |  |  |  |  | 100 | 307 | FI |  |  |  |
|  |  |  |  |  |  |  |  | 117 | 244 | EFS |  |  |  |
|  |  |  |  |  |  |  |  | 85 | 261 | CFS |  |  |  |
| Hilbrands LB [98] | Cohort study | Europe | 65.0±14.1 † | 0.61 | 26.7±5.4 † | Mixed (KTR and dialysis) | 1,073 | — | — | CFS | 257 | 28 d | Age, respiratory rate, >25% higher creatinine, Use of prednisone |
| Kakio Y [99] | Cross-sectional study | Asia (Japan) | 67.4±11.8 † | 0.61 | 21.8±3.6 † | HD | 333 | 173 | 71 | Fried phenotype | — | — | — |
| Tabinor M [100] | Cohort study | Europe (UK) | 65.1±13.6 † | 0.57 | 29.9±6.1 † | Mixed | 185 | 90 | 16 | CFS | 56 | 8 W | Age,deprivation, co-morbidities, and race. |
| Yuan H [101] | Cross-sectional study | Asia (China) | 53.2±14.4 | 0.524 | Category | HD | 187 | 68 | 11 | FRAIL Scale | — | — | — |
| Haugen CE [6] | Cohort study | North America (USA) | 52±13 | 0.39 | Not reported | KTC (Dialysis) | 4,552 | — | 561 | Fried phenotype | 903 | 5 years | Age, sex, race, BMI, cause of ESRD, and blood type. |
| Gesualdo GD [102] | Cross-sectional study | South America (Brazil) | 54.3±14.85 | 0.673 | Not reported | Dialysis | 107 | 48 | 51 | Fried phenotype | — | — | — |
| Chi CY [103] | Cross-sectional study | Asia (China) | 61.1±12.0 | 0.669 | 23.95±4.3 | HD | 151 | — | 29 | EFS | — | — | — |
|  |  |  |  |  |  |  |  |  | 19 | FRAIL Scale |  |  |  |
|  |  |  |  |  |  |  |  |  | 36 | SOF |  |  |  |
| Garcia-Canton C [104] | Cohort study | Europe (Spain) | 63.6±14.9 † | 0.657 | 27.0±4.6 † | HD | 277 | 53 | 82 | EFS | Prefrail: 16  Frailty: 48 | 22 months | Charlson Comorbidity Index without age, BMI, albumin, and creatin kinase. |
| Soldati A [105] | Cohort study | Europe (Italy) | 79.1±7.6 | 0.65 | Not reported | HD | 105 | — | 58 | FI | 29 | 21 months | Age and sex. |
| Kosoku A [106] | Cross-sectional study | Asia (Japan) | 55.0±14.9 † | 0.43 | 22.4±3.7 † | KTR | 205 | 55 | 23 | Kihon Checklist | — | — | — |
| Chao CT [3] | Cohort study | Asia (China) | 61.5±14.6 † | 0.45 | Not reported | Mixed | 165,461 | 54,785 | 1,090 | FRAIL Scale | Prefrailty: 17,362  Frailty: 661 | 4.1 years | Age, sex, smoking, alcoholism, comorbidities, aDCSI, stage 5 CKD or not, cardiac procedures, medications used. |
| Dos Santos Mantovani M [107] | Longitudinal study | South America (Brazil) | 44.7±12.3 † | 0.59 | Category | KTR | 87 | — | 32 | Fried phenotype | — | — | — |
| Wei Y [108] | Cross-sectional study | Asia (China) | 59.77±10.55 | 0.692 | Not reported | HD | 143 | 45 | 38 | FRAIL Scale | — | — | — |
| Chu NM [7] | Cross-sectional study | North America (USA) | 55.0±11.9 † | 0.579 | Not reported | HD | 378 | — | 250 | Fried phenotype | — | — | — |
|  |  |  | 55.3±14.8 † | 0.592 | Not reported | KTC (Mixed) | 4,304 | — | 1,019 | Fried phenotype | — | — | — |
|  |  |  | 53.6±15.6 † | 0.6 | Not reported | KTR | 1,396 | — | 271 | Fried phenotype | — | — | — |
| Neradova A [109] | Cohort study | Europe (UK) | 65.4 ± 15.8 | 0.575 | Not reported | Mixed | 174 | — | 102 | CFS | 44 | 28 d | Age, ethnicity, obesity, stage of CKD, corbidity, C-reactive protein, the neutrophilto-lymphocyte count. |
| Bao Y [110] | Cohort study | North America (USA) | 59.6±14.2 | 0.555 | Not reported | Dialysis | 1,576 | — | 1,155 | Fried phenotype | 522 | 2.9 y | Age, gender, race, current smoker, eGFR, albumin, hemoglobin, comorbidity, dialysis modality |
| Van Loon IN [9] | Cohort study | Europe (The Netherlands) | 75±7 | 0.67 | 26±5 | Dialysis | 192 | — | 76 | CGA | 29 | 1 year | Age, sex, CIRS-G comorbidity burden, smoking, residual renal function and dialysis modality. |
|  |  |  |  |  |  |  | 192 | — | 46 | FI | 28 | 1 year | — |
|  |  |  |  |  |  |  | 192 | — | 61 | GFI | 29 | 1 year | Univariate. |
| Zhou H [111] | Cross-sectional study | North America (USA) | 64.6±8.5 † | 0.594 | 31.4±7.4 † | HD | 74 | — | 30 | Fried phenotype | — | — | — |
| Chan GC [112] | Cohort study | Asia (China) | 59.3±11.6 | 0.542 | 24.4±4.3 | PD | 432 | — | 286 | FRAIL Scale | — | — | — |
|  |  |  |  |  |  |  |  |  | 280 | CFS |  |  |  |
| Kumarasinghe AP [113] | Cross-sectional study | Oceania (Australia) | 72.7±8.0 † | 0.594 | Not reported | Predialysis | 82 | 24 | 22 | CFS | — | — | — |
| Jiang X [114] | Cohort study | North America (USA) | Category | 0.51 | Not reported | Dialysis | 1,424,026 | — | 173,371 | Scores based on ICD-9 codes | Not reported | Not reported | Age, sex, race, income, insurance status, Charlson comorbidity index, location and teaching status of hospital, and region of hospital. |
| Thomas AG [115] | Cohort study | North America (USA) | 52.6±15.6 † | 0.628 | 27.8±5.5 † | KTR | 465 | — | 62 | Fried phenotype | — | — | — |
| Tylicki L [116] | Cohort study | Europe (Poland) | 73.0±9.0 † | 0.53 | 25.6±5.2 † | HD | 133 | — | — | CFS | 53 | 3 m | Age, chronic vitamin D supplementation, blood type, C-reactive protein and D-dimer. |
| Yi C [117] | Cohort study | Asia (China) | 48.8±14.6 | 0.592 | 22.2±3.3 | PD | 784 | 94 | 216 | CFS | 110 | 28.1 m | Age, gender, education level, diabetes mellitus, cardiovascular disease, duration of dialysis, high-sensitivity C-reactive protein, albumin, sodium, creatinine, intact parathyroid hormone, eGFR, and cognitive function status. |
| Haugen CE [118] | Cross-sectional study | North America (USA) | 52.2±13.4 † | 0.543 | 29.3±6.1 † | KTC (mixed) | 4,616 | 1,142 | 612 | Fried phenotype | — | — | — |
|  |  |  | 52.2±15.4 † | 0.601 | 28.3±5.7 † | KTR | 1,763 | 421 | 235 | Fried phenotype | — | — | — |
| Worthen G [119] | Cohort study | North America (Canada) | 54±14 | 0.64 | Category | KTC (mixed) | 542 | — | 88 | Fried phenotype | — | — | — |
|  |  |  |  |  |  |  |  |  | 183 | FI |  |  |  |
|  |  |  |  |  |  |  |  |  | 81 | CFS |  |  |  |
| Wu HHL [120] | Cohort study | Europe (UK) | 83.5±9.2 | 0.37 | 23±6.1 | Mixed | 397 | 40 | 357 | CFS | Not reported | 27.2 m | Age, gender, intracapsular hip fracture, stage of CKD, Co-morbidity Index. |
| Brar RS [2] | Cohort study | North America (Canada) | 67.3±14.3 † | 0.59 | Not reported | Mixed (ESRD and dialysis) | 603 | — | 204 | Fried phenotype | 226 | 1,455 d | Age, sex, and comorbidity count. |
| Iyasere O [121] | Cross-sectional study | Europe (UK) | 83.7±6.2 † | 0.5 | Not reported | Predialysis | 28 | — | 11 | CFS | — | — | — |
|  |  |  | 82.8±7.0 † | 0.5 | Not reported | PD | 28 | — | 17 | CFS | — | — | — |
|  |  |  | 81.6±5.5 † | 0.43 | Not reported | HD | 28 | — | 11 | CFS | — | — | — |
| Shrestha P [122] | Cross-sectional study | American | 55±13 | 0.6 | Not reported | KTC (mixed） | 1,003 | 286 | 192 | Fried phenotype | — | — | — |
| Lee S [123] | Cross-sectional study | Asian | 73.6±5.5 | 0.458 | 23±3 | Mixed | 8,343 | — | 712 | Fried phenotype | — | — | — |
| Santos DGMD [124] | Cross-sectional study | South American | 59.63±14.14 | 0.45 | Not reported | HD | 80 | — | 75 | TFI | — | — | — |
| Chen X [8] | Cohort study | North America (USA) | 52.9±13.8 | 0.614 | 27.2±5.4 | KTR | 1,113 | 722 | 207 | Fried phenotype | 236 | 6.3 y | Age at KT, sex, Black race, donor type and Charlson comorbidity index. |
| Heybeli C [125] | Cross-sectional study | Asia (Turkey) | 77.4±8.2 † | 0.317 | 30.8±6.6 † | Predialysis (stage 3-4) | 205 | — | 85 | Fried phenotype | — | — | — |
| Carvalho TC [126] | Cross-sectional study | South America (Brazil) | 56.09 | 0.526 | Not reported | HD | 103 | — | 37 | TFI | — | — | — |
| Zanotto T [11] | Cross-sectional study | Europe (UK) | 61.1±14 | 0.539 | 29.0±6.3 † | HD | 76 | 42 | 28 | Fried phenotype | — | — | — |
| Fu W [127] | Cohort study | Asia (China) | 60.5±12.7 | 0.543 | 20.9±4.0 † | HD | 208 | 95 | 53 | Fried phenotype | — | — | — |
| Demircioglu DT [128] | Cross-sectional study | Asia (Turkey) | 50.91±10.14 | 0.55 | 24.89±5.23 | HD | 74 | 13 | 39 | Fried phenotype | — | — | — |
| Jafari M [129] | Cohort study | North America (Canada) | 62.86±15.44 | 0.58 | 29.33±7.26 | HD | 100 | 26 | 66 | Fried phenotype | — | — | — |
| Schaenman J [130] | Cross-sectional study | North America (USA) | 52.4±13.0 † | 365 | Not reported | KTR | 60 | — | 17 | FRS | — | — | — |
| Brar R [131] | Cohort study | North America (Canada) | 55.6±16.8 † | 0.33 | Not reported | Dialysis | 109 | — | 31 | Fried phenotype | 38 | 3.3 y | Age, sex, albumin, hemoglobin and comorbidity count. |
| van Loon IN [132] | Cohort study | Europe (UK) | 75±7 | 0.6 | Not reported | Dialysis | 203 | — | — | CSHA Clinical Frailty Scale | 59 | 2 y | Age, gender, dialysis modality, depression, use of sedative frugs, falls. |
| Zhang B [133] | Cross-sectional study | Asia (China) | 45.17 ±12.95 | 0.659 | Not reported | KTR | 185 | 33 | 18 | TFI | — | — | — |
| Gong WY [134] | Cross-sectional study | Asia (China) | 61.96±13.68 | 0.61 | 22.2±3.61 | HD | 300 | — | 225 | TFI | — | — | — |
| Montesanto A [135] | Cohort study | European (Italy) | 83.4±11.5 † | 0.53 | Not reported | Predialysis (CKD 3b-5) | 1,038 | 288 | 530 | Fried phenotype | — | — | — |
| Rodriguez VI [136] | Cross-sectional study | Europe (Spain) | 79±4.98 | 0.52 | Not reported | ESRD (predialysis) | 56 | 28 | 0 | Fried phenotype | — | — | — |
| Shlipak MG [137] | Cohort study | South America (USA) | 76 | 0.61 | Not reported | Mixed | 648 | — | 80 | Fried phenotype | — | — | — |
| Yadla M [138] | Cohort study | Asia (India) | 44.95±13.27 | 0.69 | Category | HD | 205 | — | 167 | Fried phenotype | Not reported | 1 year | Not reported |
| Painter P [139] | Cross-sectional study | North America (USA) | 54.4±15.8 | 0.436 | 26.4±6.3 | HD | 188 | — | 147 | Fried phenotype | — | — | — |
| Overlapping populations, 48 articles not included in the analysis | | | | | | | | | | | | | |
| Johansen KL [140] | Cross-sectional study | North America (USA) | 56.8±14.5 | 0.58 | 29.0±7.1 | HD | 638 | — | 190 | Fried phenotype | — | — | — |
| Ghazi L [141] | Cohort study | North America (USA) | 63±10 | 0.56 | Not reported | Predialysis | 1,464 | — | 275 | Fried phenotype | — | — | — |
| Goto NA [142] | Cross-sectional study | Europe (The Netherlands) | 75±7 | 0.67 | Not reported | Dialysis | 187 | — | 148 | CGA | — | — | — |
|  |  |  |  |  |  |  |  |  | 82 | FI |  |  |  |
|  |  |  |  |  |  |  |  |  | 115 | GFI |  |  |  |
| Johansen KL [143] | Cohort study | North America (USA) | 57.1±14.2 | 0.593 | 28.2±6.9 | HD | 762 | — | 240 | Fried phenotype | 73 | 1.7 y | Age, sex, race, BMI, diabetes, heart failure, and coronary artery disease, serum albumin, C–reactive protein and dialysis via central venous catheter. |
| McAdams-DeMarco MA [144] | Longitudinal study | North America (USA) | 53.3±14.2 | 0.619 | Not reported | KTR | 349 | 120 ^②^ | 69 | Fried phenotype | — | — | — |
| Johansen KL [145] | Cross-sectional study | North America (USA) | 59±14 | 0.59 | 27.8±6.4 | HD | 68 | — | 23 | Fried phenotype | — | — | — |
| Chao CT [146] | Cohort study | Asia (Japan) | 67.2±12.4 | 0.446 | Not reported | Mixed | 36,081 | 22,132 | 8652 | Fried Scale | — | — | — |
| Johansen KL [147] | Cross-sectional study | North America (USA) | 57.3±14.0 | 0.59 | 29.1±7.1 | HD | 731 | — | 232 | Fried phenotype | — | — | — |
| Kutner NG [148] | Cross-sectional study | North America (USA) | 57.2±14.1 | 0.594 | Category | HD | 742 | 433 | 104 | Fried phenotype | — | — | — |
| Chan GC [149] | Cohort study | Asia (China) | 62.9±12 | 0.49 | Not reported | PD | 267 | 74 | 120 | Chinese Frailty Score | — | — | — |
| Sy J [150] | Cohort study | North America (USA) | 57.2±14.2 | 0.594 | 28.9±6.9 | Dialysis | 746 | — | 238 | Fried phenotype |  |  | Age, sex, race, comorbidities, and inflammatory markers (interleukin-6 and C-reaction protein). |
| Kang SH [151] | Cohort study | Asia (Korea) | 56.4±13.2 | 0.566 | 22.1±3.2 | HD | 1,250 | 578 | 422 | Fried phenotype | 61 | 489 d | Age, sex, BMI, education level, dialysis vintage, diabetes mellitus, cerebrovascular disease, coronary artery disease, albumin, urea nitrogen, creatinine, intact parathyroid hormone, and total cholesterol. |
|  |  |  | 54.1±11.9 | 0.533 | 23.5±3.1 | PD | 366 | 165 | 137 |  | 25 |  |  |
| Chao CT [152] | Cross-sectional study | Asia (China) | 69.1 ± 9.1 | 0.472 | 22.8 ± 3.2 | HD | 43 | 22 | 6 | FRAIL Scale | — | — | — |
| Garonzik-Wang JM [153] | Cross-sectional study | North America (USA) | 53.4±14.0 | 0.637 | 26.6±5.6 | KTR | 183 | — | 46 | Fried phenotype | — | — | — |
| McAdams-DeMarco MA [154] | Cross-sectional study | North America (USA) | 53.5±13.9 | 0.603 | 27.4±5.4 | KTR | 383 | — | 72 | Fried phenotype | — | — | — |
| McAdams-DeMarco MA [155] | Cohort study | North America (USA) | 60.5±12.6 | 0.537 | 29.1±8.4 | HD | 95 | 27 | 44 | Fried phenotype | — | — | — |
| Walker SR [156] | Cross-sectional study | North America (Canada) | 69.8±14.3 | 0.6 | Not reported | Predialysis | 217 | — | 122 | TUGT | — | — | — |
| Lee SY [157] | Cohort study | Asia (China) | 61±14.2 | 0.558 | Not reported | Mixed | 149,145 | 100,951 | 1,912 | FRAIL Scale | — | — | — |
| McAdams-DeMarco MA [158] | Cohort study | North America (USA) | 53±14 | 0.612 | 27.5±5.9 | KTR | 525 | 174 | 102 | Fried phenotype | — | — | — |
| Goto NA [159] | Cross-sectional study | Europe (The Netherlands) | 75±7 | 0.67 | 27±5 | Dialysis | 196 | — | 85 | FI | — | — | — |
|  |  |  |  |  |  |  |  |  | 121 | GFI |  |  |  |
|  |  |  | 82±6 | 0.56 | 26±5 | Predialysis (stage 5) | 89 | — | 32 | FI | — | — | — |
|  |  |  |  |  |  |  |  |  | 57 | GFI |  |  |  |
| Chao CT [160] | Cross-sectional study | Asia (China) | 68±11.8 | 0.43 | Not reported | HD | 51 | 24 | 10 | FRAIL Scale | — | — | — |
| McAdams-DeMarco MA [161] | Cohort study | North America (USA) | 53±13.9 | 0.62 | Category | KTR | 663 | 210 | 129 | Fried phenotype | — | — | — |
| Chu NM [162] | Longitudinal study | North America (USA) | 52.7±14.2 | 0.603 | Not reported | KTR | 1,336 | — | 208 | Fried phenotype | — | — | — |
| Chiang JM [163] | Cohort study | North America (USA) | 56.1±14.2 | 0.96 | 28.2±6.5 | HD | 440 | — | 127 | Fried phenotype | — | — | — |
| Lee SY [164] | Cohort study | Asia (China) | 66.5±15.1 | 0.42 | Not reported | Mixed | 23,274 | 18,348 | — | Fried phenotype | — | — | — |
| Haugen CE [165] | Cohort study | North America (USA) | 54±14 | 0.6 | Not reported | KTC (mixed) | 3,143 | — | 1,821 | Fried phenotype | — | 5 years | — |
| Reese PP [166] | Cohort study | North America (USA) | 65.0±2.1 | 0.53 | 30.0±1.4 | Mixed | 1,111 | 447 | 73 | Fried phenotype | — | — | — |
| Kimura H [167] | Cohort study | North America (USA) | 56±13 | 0.55 | 27.9±1.7 | HD | 337 | — | 94 | Fried phenotype | — | — | — |
| Kutner NG [168] | Cohort study | North America (USA) | 57.1±14.1 | 0.595 | 28.2±6.9 | HD | 745 | — | 103 | Fried phenotype | — | — | — |
| Chao CT [169] | Cross-sectional study | Asia (Japan) | 67.3±11.9 | 0.43 | 22.9±3.0 | HD | 46 | — | 32 | SF | — | — | — |
|  |  |  |  |  |  |  |  |  | 20 | EFS |  |  |  |
|  |  |  |  |  |  |  |  |  | 9 | FRAIL Scale |  |  |  |
|  |  |  |  |  |  |  |  |  | 25 | GFI |  |  |  |
|  |  |  |  |  |  |  |  |  | 38 | G8 questionnaire |  |  |  |
|  |  |  |  |  |  |  |  |  | 14 | TFI |  |  |  |
| Kang SH [170] | Cohort study | Asia (Korea) | 55.9±13.0 | 0.78 | 22.4±3.2 | Dialysis | 1,161 | 741 | 557 | Fried phenotype | — | — | — |
| Kang SH [171] | Cohort study | Asia (Korea) | 55.8±12.9 | 0.559 | 22.4±3.2 | Dialysis | 1,615 | — | 558 | Fried phenotype | — | — | — |
| Haugen CE [172] | Cohort study | North America (USA) | 54±13 | 0.657 | Not reported | KTC (mixed) | 1,154 | — | 219 | Fried phenotype | — | — | — |
|  |  |  | 53±14 | 0.659 | Not reported | KTR | 378 | — | 55 | Fried phenotype | — | — | — |
|  |  |  |  |  |  |  |  |  | 152 | IL6-Fried phenotype | 28 | 5 y | Recipient age, sex, Charlson Comorbidity Index, race, cause of ESRD, and living donor. |
|  |  |  |  |  |  |  |  |  | 144 | TNFa-Fried phenotype | 24 | 5 y |  |
|  |  |  |  |  |  |  |  |  | 153 | CRP-Fried phenotype | 19 | 5 y |  |
| Chao CT [173] | Cross-sectional study | Asia (China) | 68.9±10.4 | 0.37 | 23.5±4 | HD | 35 | — | 19 | FRAIL Scale | — | — | — |
| Pérez-Sáez MJ [174] | Cohort study | Europe (Spain) | 60.6±12.4 | 0.684 | 27.8±12.4 | KTC (dialysis) | 455 | 91 | 47 | Fried phenotype | — | — | — |
| Johansen KL [175] | Cohort study | North America (USA) | 57.2±14.3 | 0.592 | 28.1±6.9 | HD | 727 | 409 | 230 | Fried phenotype | 204 | 3.8 y | Age, sex, race, ethnicity, BMI, diabetes, atherosclerotic heart disease, heart failure, dialysis via a catheter, and albumin. |
| Chu NM [176] | Cohort study | North America (USA) | 52±14.2 | 0.612 | Not reported | KTR | 665 | — | 100 | Fried phenotype | — | — | — |
| McAdams-DeMarco MA [177] | Cohort study | North America (USA) | 53±14 | 0.6 | 27.4±5.9 | KTR | 537 | 177 | 107 | Fried phenotype | 50 | 2.7 years | Recipient age, sex, race, diabetes, time on dialysis and preemptive KT, donor type and cold ischemia time. |
| McAdams-DeMarco MA [178] | Cohort study | North America (USA) | 60.6±13.6 | 0.534 | Not reported | HD | 146 | 47 | 61 | Fried phenotype | 67 | 3 years | Age, sex, comorbidity, and disability. |
| Pérez-Sáez MJ [179] | Cross-sectional study | Europe (Spain) | 60.9±12.2 | 0.683 | 27.9±5.3 | KTC (Mixed) | 451 | 275 | 47 | Fried phenotype | — | — | — |
|  |  |  |  |  |  |  | 451 | 181 | 24 | FRAIL Scale |  |  |  |
| Nixon AC [180] | Cross-sectional study | Europe (UK) | 69±13 | 0.5 | 29±6 | Mixed | 90 | — | 19 | Fried phenotype | — | — | — |
| Lee SY [181] | Cohort study | Asia (China) | 62.9±13.6 | 0.52 | Not reported | Mixed | 52,058 | 40,516 | 969 | FRAIL Scale | — | — | — |
| Chan GCK [182] | Cohort study | Asia (China) | 62.5±12.1 | 0.49 | 25.8±4.2 | PD | 267 | — | 120 | FRAIL Scale | — | — | — |
| Thomas AG [183] | Cohort study | North America (USA) | 53.0±3.3 | 0.616 | 28.0±1.3 | KTR | 864 | — | 144 | Fried phenotype | — | — | — |
| Chu NM [184] | Cohort study | North America (USA) | 54±14 | 0.619 | 28.9±6.5 | KTC (mixed) | 3,666 | 1,122 | 766 | Fried phenotype | — | — | — |
|  |  |  | 54.3±14.0 | 0.36 | 28.1±5.9 | KTR | 770 | 237 | 119 |  |  |  |  |
| Nastasi AJ [185] | Cohort study | North America (USA) | 51.6±14.2 | 0.623 | Not reported | KTR | 719 | — | 113 | Fried phenotype | — | — | — |
| Sy J [186] | Cohort study | North America (USA) | 56.8±13.3 | 0.576 | 29.2±7.1 | HD | 425 | — | 125 | Fried phenotype | — | — | — |
| Zanotto T [187] | Cross-sectional study | Europe (UK) | 61.7±13.3 | 0.449 | 29.1±6.4 | HD | 69 | — | 26 | Fried phenotype | — | — | — |

USA, the United State; UK, the United Kingdom; SFA, Subjective Frailty Assessment; EFS, Edmonton Frail Scale; SOF, Study of Osteoporotic Fractures Scale; CSHA-CFS, Canadian Study of Health and Aging Scale; GFI, Groningen Frailty Index; FI, Frailty Index; CGA, Comprehensive Geriatric Assessment; SF, Strawbridge frailty questionnaire; TFI, Tilburg Frailty Indicator; FRS, Frailty Risk Score; KTC, Kidney Transplant Candidates; KTR, Kidney Transplant Recipient; HD, hemodialysis; PD, peritoneal dialysis; CKD, chronic kidney disease; BMI, body mass index; eGFR, estimated glomerular filtration rate; PRISMA, Programon Research for Integrating Services for the Maintenance of Autonomy; ICD-9, International Classification of Diseases-9; ESRD, end-stage renal disease

①† indicates the data converted by the formula.

②Data from Figure via Getdata software.

Table S7 Assessment of risk of bias in the included studies using Newcastle-Ottawa Scale (NOS)

| Author | Study design | Selection | | | | Comparability | Outcome | | |
| --- | --- | --- | --- | --- | --- | --- | --- | --- | --- |
|  |  | Representativeness of the exposed cohort | Selection of the non-exposed cohort | Ascertainment of exposure | Demonstration that outcome of interest was not present at start of study | Controls for age or other factors | Ascertainment of outcome | Was follow-up long enough for outcomes to occur (3 years) | Adequacy of follow up of cohorts |
| Roshanravan B 2012 | Cohort study | a | a | b | a | a/b | b | b | a |
| Davenport A 2022 | Cross-sectional study | b | a | b | — | a/b | — | — | — |
| Ali H 2018 | Cohort study | b | a | b | a | b | b | b | a |
| Orlandi FD 2014 | Cross-sectional study | b | a | b | — | NP | — | — | — |
| Kosaka S 2020 | Cross-sectional study | b | a | b | — | a/b | — | — | — |
| Johansen KL 2014 | Cross-sectional study | b | a | b | — | a/b | — | — | — |
| Yoneki K 2019 | Cross-sectional study | b | a | b | — | b | — | — | — |
| Santos DGMD 2022 | Cross-sectional study | b | a | b | — | a/b | — | — | — |
| Usui N 2021 | Cross-sectional study | b | a | b | — | a/b | — | — | — |
| Ghazi L 2020 | Cohort study | b | a | b | a | a/b | b | b | a |
| Yabuuchi J 2020 | Cross-sectional study | b | a | b | — | NP | — | — | — |
| Delgado C 2015 | Cohort study | a | a | b | a | a | b | a | b |
| Delgado C 2013 | Cross-sectional study | b | a | b | — | a/b | — | — | — |
| Mansur HN 2015 | Longitudinal studies | b | a | b | a | a/b | b | b | b |
| Luo CM 2022 | Cohort study | a | a | b | a | a | b | b | b |
| Goto NA 2019 | Cross-sectional study | b | a | b | — | a/b | — | — | — |
| Johansen KL 2016 | Cohort study | b | a | b | a | a/b | b | b | a |
| Delgado C 2015 | Cohort study | a | a | c | a | a/b | c | b | a |
| Smith G 2021 | Cross-sectional study | b | a | b | — | a/b | — | — | — |
| Zhang R 2020 | Cross-sectional study | b | a | b | — | b | — | — | — |
| Lysak N 2020 | Cohort study | b | a | b | a | a/b | b | a | b |
| McAdams-DeMarco MA 2015 | Longitudinal studies | b | a | b | a | a/b | b | b | b |
| Johansen KL 2015 | Cross-sectional study | b | a | b | — | NP | — | — | — |
| Chao CT 2020 | Cohort study | a | a | a | a | a | c | b | b |
| Kim JC 2022 | Cross-sectional study | b | a | b | — | b | — | — | — |
|  |  |  |  |  |  |  |  |  |  |
| Fernandez MP 2019 | Cohort study | b | b | c | a | a/b | b | b | a |
| Davenport A 2022 | Cross-sectional study | b | a | b | — | a/b | — | — | — |
| Johansen KL 2014 | Cross-sectional study | b | a | b | — | NP | — | — | — |
| Kim JC 2021 | Cross-sectional study | b | a | b | — | NP | — | — | — |
| Kutner NG 2014 | Cross-sectional study | b | a | b | — | a/b | — | — | — |
| Szeto CC 2018 | Cohort study | b | a | b | a | NP | c | b | a |
| Chan GC 2020 | Cohort study | b | a | b | a | NP | b | b | a |
| Konel JM 2018 | Cohort study | b | a | b | a | a/b | b | b | a |
| Sy J 2019 | Cohort study | b | a | b | a | a/b | b | b | a |
| Nixon AC 2019 | Cross-sectional study | b | a | b | — | NP | — | — | — |
| van Munster BC 2016 | Cross-sectional study | b | a | b | — | NP | — | — | — |
| Kang SH (1) 2017 | Cohort study | a | a | b | a | a/b | b | b | a |
| Van Pilsum, Rasmussen S 2018 | Cross-sectional study | b | a | b | — | NP | — | — | — |
| Johansen KL 2017 | Cohort study | a | a | b | a | a/b | b | b | a |
| Hubbard RE 2015 | Cross-sectional study | b | a | b | — | NP | — | — | — |
| Bancu I 2017 | Cross-sectional study | b | a | b | — | NP | — | — | — |
| Chao CT 2017 | Cross-sectional study | b | a | b | — | NP | — | — | — |
| Wilhelm-Leen ER 2009 | Cohort study | a | a | b | a | a/b | b | a | b |
| McAdams-DeMarco MA 2015 | Cohort study | a | a | b | a | a/b | c | b | a |
| Pugh J 2016 | Cohort study | a | a | c | a | a/b | b | a | a |
| Garonzik-Wang JM 2012 | Cross-sectional study | b | a | b | — | a/b | — | — | — |
| McAdams-DeMarco MA 2013 | Cross-sectional study | b | a | b | — | NP | — | — | — |
| McAdams-DeMarco MA 2013 | Cohort study | b | a | b | a | a/b | c | b | a |
| McAdams-DeMarco MA 2016 | Longitudinal studies | b | a | b | a | NP | c | b | a |
| Alfaadhel TA 2015 | Cohort study | b | a | b | a | a/b | b | b | a |
| Walker SR 2015 | Cross-sectional study | b | a | b | — | a/b | — | — | — |
| Mansur HN 2014 | Cross-sectional study | b | a | b | — | NP | — | — | — |
| Noori N 2018 | Cross-sectional study | b | a | b | — | a/b | — | — | — |
| SchopmeyerL 2019 | Cross-sectional study | b | a | b | — | a/b | — | — | — |
| Ng JK 2016 | Cohort study | b | a | b | a | a/b | b | b | a |
| Poveda V 2017 | Cross-sectional study | b | a | b | — | b | — | — | — |
| Clark DA 2017 | Cross-sectional study | b | a | b | — | NP | — | — | — |
| Guo Y 2022 | Cohort study | b | a | b | a | a/b | b | b | a |
| Lee SY 2021 | Cohort study | a | a | b | a | a/b | b | a | b |
| Vettoretti S 2020 | Cross-sectional study | b | a | b | — | NP | — | — | — |
| Bloomfield K 2021 | Cohort study | b | a | b | a | a/b | b | a | a |
| Clark D 2021 | Cohort study | a | a | b | a | a/b | b | b | b |
| Moreno-Useche LD 2021 | Cross-sectional study | b | a | b | — | NP | — | — | — |
| Fitzpatrick J 2019 | Cohort study | b | a | b | a | a/b | b | b | a |
| Tsai MD 2022 | Cross-sectional study | b | a | b | — | a/b | — | — | — |
| Adame Perez SI 2019 | Cross-sectional study | b | a | b | — | NP | — | — | — |
| McAdams-DeMarco MA 2018 | Cohort study | a | a | b | a | a/b | b | b | a |
| McAdams-DeMarco MA 2015 | Cohort study | a | a | b | a | a/b | b | a | a |
| Lopez-Montes A 2020 | Cohort study | b | a | b | a | a/b | b | b | a |
| Hornik B 2019 | Cross-sectional study | b | a | b | — | b | — | — | — |
| Goto NA 2019 | Cross-sectional study | b | a | b | — | NP | — | — | — |
| Chao CT 2016 | Cross-sectional study | b | a | b | — | NP | — | — | — |
| Meulendijks FG 2015 | Cohort study | b | a | b | a | NP | b | b | a |
| Drost D 2016 | Cross-sectional study | b | a | b | — | NP | — | — | — |
| Weng SC 2021 | Cohort study | b | a | b | a | a/b | b | a | a |
| McAdams-DeMarco MA 2017 | Cohort study | b | a | b | a | a/b | b | a | a |
| Lee SJ 2015 | Cross-sectional study | b | a | b | — | a/b | — | — | — |
| Lee SW 2017 | Cohort study | b | a | b | a | a/b | b | b | a |
| Warsame F 2019 | Cohort study | a | a | b | a | a/b | b | b | a |
| Guo F 2021 | Cross-sectional study | b | a | b | — | a/b | — | — | — |
| Chu NM 2022 | Longitudinal study | b | a | b | a | NP | b | a | a |
| Chiang JM 2019 | Cohort study | a | a | b | a | a/b | b | b | a |
| Polinder-Bos HA 2017 | Cohort study | b | a | b | a | a/b | b | b | a |
| Mentias A 2020 | Cohort study | a | a | a | a | a/b | b | b | a |
|  |  |  |  |  |  |  |  |  |  |
| Hernandez-Agudelo SY 2021 | Cross-sectional study | b | a | b | — | NP | — | — | — |
| Pérez-Sáez MJ 2022 | Cohort study | b | a | b | a | a/b | b | b | a |
| Farragher JF 2019 | Cross-sectional study | b | a | b | — | NP | — | — | — |
| Salter ML 2015 | Cross-sectional study | b | a | b | — | NP | — | — | — |
| Okuyama M 2018 | Cross-sectional study | b | a | b | — | a/b | — | — | — |
| Haugen CE 2020 | Cohort study | a | a | b | a | a/b | b | b | a |
| Reese PP 2013 | Cohort study | a | a | a | a | a/b | b | b | a |
| Kimura H 2021 | Cohort study | a | a | b | a | a/b | b | b | a |
| Meyer AM 2022 | Cohort study | b | a | b | a | a/b | b | b | a |
| Chan GCK 2021 | Longitudinal study | b | a | b | a | NP | b | b | a |
| Iyasere OU 2016 | Cross-sectional study | b | a | b | — | a/b | — | — | — |
| Candemir B 2022 | Cross-sectional study | b | a | b | — | NP | — | — | — |
| Kutner NG 2014 | Cohort study | a | a | b | a | a/b | b | b | a |
| Kamijo Y 2018 | Cross-sectional study |  |  |  |  |  |  |  |  |
| Johansen KL 2007 | Cohort study | a | a | b | a | a/b | b | b | a |
| Chao CT 2015 | Cross-sectional study | b | a | b | — | NP | — | — | — |
| Slaven A 2021 | Cross-sectional study | b | a | b | — | a/b | — | — | — |
| Wang J 2022 | Cohort study | b | a | b | a | a/b | b | b | b |
| Inoue T 2021 | Cross-sectional study | b | a | b | — | a/b | — | — | — |
| Zachciał J 2022 | Cross-sectional study | b | a | b | — | a/b | — | — | — |
| Kang SH 2017 | Cohort study | b | a | b | a | a/b | b | b | b |
| Mutevelić-Turković A 2022 | Cross-sectional study | b | a | b | — | NP | — | — | — |
| Li Y 2021 | Cohort study | b | a | b | a | a/b | b | b | b |
| Chen CH 2022 | Cohort study | b | a | b | a | a/b | b | b | b |
| Takeuchi H 2018 | Cross-sectional study | b | a | b | — | a/b | — | — | — |
| Gopinathan JC 2020 | Cross-sectional study | b | a | b | — | NP | — | — | — |
| Lee SY 2017 | Cohort study | b | a | b | a | a/b | b | b | a |
| Kang SH 2021 | Cohort study | b | a | b | a | a/b | b | b | a |
| Jegatheswaran J 2020 | Cohort study | b | a | b | a | a | a | b | a |
| Lorenz EC 2019 | Cohort study | b | a | b | a | b | a | b | b |
| Miller LM 2021 | Cross-sectional study | b | a | b | — | a/b | — | — | — |
| Saitoh M 2020 | Cross-sectional study | b | a | b | — | a/b | — | — | — |
| Hendra H 2022 | Cross-sectional study | b | a | b | — | a/b | — | — | — |
| Davenport A 2022 | Cross-sectional study | b | a | b | — | a/b | — | — | — |
| Imamura K 2022 | Cohort study | b | a | b | a | a/b | b | b | b |
| Anderson BM 2022 | Cross-sectional study | b | a | b | — | NP | — | — | — |
|  |  |  |  |  |  |  |  |  |  |
| Haugen CE (1) 2021 | Cohort study | b | a | b | a | a/b | b | b | a |
| Kakio Y 2018 | Cross-sectional study | b | a | b | — | a/b | — | — | — |
| Tabinor M 2022 | Cohort study | b | a | b | a | a/b | b | b | a |
| Yuan H 2020 | Cross-sectional study | b | a | b | — | a/b | — | — | — |
| Chao CT 2017 | Cross-sectional study | b | a | b | — | NP | — | — | — |
| Pérez-Sáez MJ 2022 | Cross-sectional study | b | a | b | — | a/b | — | — | — |
| Johansen KL 2019 | Cohort study | a | a | b | a | a/b | b | b | a |
| Haugen CE 2019 | Cohort study | a | a | b | a | a/b | b | a | a |
| Gesualdo GD 2020 | Cross-sectional study | b | a | b | — | a/b | — | — | — |
| Chu NM 2019 | Cohort study | a | a | b | a | a/b | c | b | a |
| McAdams-DeMarco MA 2015 | Cohort study | b | a | b | a | a/b | b | b | a |
| McAdams-DeMarco MA 2013 | Cohort study | b | a | b | a | a/b | b | a | a |
| Chi, C Y 2022 | Cross-sectional study | b | a | b | — | b | — | — | — |
| Garcia-Canton C 2019 | Cohort study | b | a | b | a | a/b | b | b | a |
| Pérez-Sáez MJ 2022 | Cross-sectional study | b | a | b | — | NP | — | — | — |
| Soldati A 2022 | Cohort study | b | a | b | a | a | b | b | a |
| Kosoku A 2020 | Cross-sectional study | b | a | b | — | NP | — | — | — |
| Nixon C2019 | Cross-sectional study | b | a | b | — | NP | — | — | — |
| Lee SY 2020 | Cohort study | a | a | b | a | a/b | b | a | b |
| Chao CT 2019 | Cohort study | a | a | b | a | a/b | b | a | b |
| Dos Santos Mantovani M 2020 | Longitudinal study | b | a | b | a | NP | b | b | a |
| Wei Y 2021 | Cross-sectional study | b | a | b | — | a/b | — | — | — |
| Chu NM 2020 | Cross-sectional study | b | a | b | — | NP | — | — | — |
| Neradova A 2021 | Cohort study | b | a | b | a | a/b | b | b | a |
| Bao Y 2012 | Cohort study | a | a | b | a | a/b | b | a | a |
| Van Loon IN 2019 | Cohort study | b | a | b | a | a/b | b | b | a |
| Zhou H 2018 | Cross-sectional study | b | a | b | — | NP | — | — | — |
| Chan GC 2021 | Cohort study | b | a | b | a | a/b | b | a | a |
| Kumarasinghe AP 2021 | Cross-sectional study | b | a | b | — | NP | — | — | — |
| Jiang X 2020 | Cohort study | a | a | a | a | a/b | b | a | b |
| Chan GCK 2020 | Cohort study | b | a | b | a | a/b | b | b | a |
| Thomas AG 2019 | Cohort study | a | a | b | a | a/b | b | a | a |
| Thomas AG 2020 | Cohort study | a | a | c | a | a/b | b | a | a |
| Chu NM 2020 | Cohort study | a | a | c | a | a/b | b | b | a |
| Nastasi AJ 2018 | Cohort study | a | a | b | a | a/b | b | b | b |
| Yi C 2018 | Cohort study | b | a | b | a | a/b | b | b | a |
| Haugen CE 2020 | Cross-sectional study | a | a | b | — | NP | — | — | — |
| Worthen G 2021 | Cohort study | b | a | b | a | a | b | b | a |
| Wu HH 2021 | Cohort study | b | a | b | a | a/b | b | b | a |
| Brar RS 2021 | Cohort study | b | a | b | a | a/b | b | a | a |
| Iyasere O 2019 | Cross-sectional study | b | a | b | — | a/b | — | — | — |
| Shrestha P 2019 | Cross-sectional study | b | a | b | — | a/b | — | — | — |
| Lee S 2017 | Cross-sectional study | a | a | b | — | a/b | — | — | — |
| Santos DGMD 2021 | Cross-sectional study | b | a | b | — | NP | — | — | — |
| Chen X 2022 | Cohort study | b | a | b | a | a/b | b | a | a |
| Heybeli, C 2021 | Cross-sectional study | b | a | b | — | NP | — | — | — |
| Carvalho TC 2020 | Cross-sectional study | b | a | b | — | NP | — | — | — |
| Zanotto T 2021 | Cross-sectional study | b | a | b | — | a/b | — | — | — |
| Fu W 2021 | Cohort study | b | a | b | a | a/b | b | b | a |
| Demircioglu DT 2018 | Cross-sectional study | b | a | b | — | NP | — | — | — |
| Jafari M 2020 | Cohort study | b | a | b | a | a/b | b | b | a |
| Schaenman J 2019 | Cross-sectional study | b | a | b | — | NP | — | — | — |
| Brar R 2019 | Cohort study | b | a | b | a | a/b | b | a | a |
| Sy J 2020 | Cohort study | a | a | b | a | a/b | b | b | a |
|  |  |  |  |  |  |  |  |  |  |
| Zanotto T 2020 | Cross-sectional study | b | a | b | — | a/b | — | — | — |
| Zhang B 2021 | Cross-sectional study | b | a | b | — | a/b | — | — | — |
| Gong WY 2022 | Cross-sectional study | b | a | b | — | a/b | — | — | — |
| Montesanto A 2014 | Cohort study | b | a | a | a | a/b | b | b | a |
| Rodriguez VI 2014 | Cohort study | b | a | b | a | NP | b | b | a |
| Shlipak MG 2004 | Cohort study | a | a | b | a | a/b | b | b | a |
| Yadla M 2017 | Cohort study | b | a | b | a | a/b | b | b | a |
| Painter P 2012 | Cross-sectional study | b | a | b | — | a/b | — | — | — |

Abbreviation: NP, not report.

Description of the NOS scale

**Newcastle - Ottawa Scale for Cross-sectional studies**

Note: A study can be awarded a maximum of one star for each numbered item within the Selection and Exposure categories. A maximum of two stars can be given for Comparability.

**Selection**

1) Is the case definition adequate?

a) yes, with independent validation **🟑**

b) yes, eg record linkage or based on self reports

c) no description

2) Representativeness of the cases

a) consecutive or obviously representative series of cases **🟑**

b) potential for selection biases or not stated

3) Selection of Controls

a) community controls **🟑**

b) hospital controls

c) no description

4) Definition of Controls

a) no history of disease (endpoint) **🟑**

b) no description of source

**Comparability**

1) Comparability of cases and controls on the basis of the design or analysis

a) study controls for _______________ (Select the most important factor.) **🟑**

b) study controls for any additional factor **🟑** (This criteria could be modified to indicate specific control for a second important factor.)

**Exposure**

1) Ascertainment of exposure

a) secure record (eg surgical records) **🟑**

b) structured interview where blind to case/control status **🟑**

c) interview not blinded to case/control status

d) written self report or medical record only

e) no description

2) Same method of ascertainment for cases and controls

a) yes **🟑**

b) no

3) Non-Response rate

a) same rate for both groups **🟑**

b) non respondents described

c) rate different and no designation

**Newcastle - Ottawa Scale for Cohort studies**

Note: A study can be awarded a maximum of one star for each numbered item within the Selection and Outcome categories. A maximum of two stars can be given for Comparability

**Selection**

1) Representativeness of the exposed cohort

a) truly representative of the average _______________ (describe) in the community **🟑**

b) somewhat representative of the average ______________ in the community **🟑**

c) selected group of users eg nurses, volunteers

d) no description of the derivation of the cohort

2) Selection of the non exposed cohort

a) drawn from the same community as the exposed cohort **🟑**

b) drawn from a different source

c) no description of the derivation of the non exposed cohort

3) Ascertainment of exposure

a) secure record (eg surgical records) **🟑**

b) structured interview **🟑**

c) written self report

d) no description

4) Demonstration that outcome of interest was not present at start of study

a) yes **🟑**

b) no

**Comparability**

1) Comparability of cohorts on the basis of the design or analysis

a) study controls for _____________ (select the most important factor) **🟑**

b) study controls for any additional factor **🟑** (This criteria could be modified to indicate specific control for a second important factor.)

**Outcome**

1) Assessment of outcome

a) independent blind assessment **🟑**

b) record linkage **🟑**

c) self report

d) no description

2) Was follow-up long enough for outcomes to occur

a) yes (select an adequate follow up period for outcome of interest) **🟑**

b) no

3) Adequacy of follow up of cohorts

a) complete follow up - all subjects accounted for **🟑**

b) subjects lost to follow up unlikely to introduce bias - small number lost - > ____ % (select an adequate %) follow up, or description provided of those lost) **🟑**

c) follow up rate < ____% (select an adequate %) and no description of those lost

d) no statement

Table S8 Meta-regression for the prevalence of frailty

| Variable | Coefficient | Standard error | Lower 95% CI | Upper 95% CI | *P* | R^2^ |
| --- | --- | --- | --- | --- | --- | --- |
| Mean age (k=137)^†^ | 0.003 | 0.003 | -0.003 | 0.010 | 0.347 | 0.00% |
| Male (k=141)^§^ | -0.000 | 0.001 | -0.001 | 0.001 | 0.762 | 0.03% |
| Mean BMI (k=81)^‡^ | -0.009 | 0.009 | -0.027 | 0.009 | 0.322 | 0.00% |
| Geographical location |  |  |  |  |  | 0.00% |
| Asia (Ref.) | — | — | — | — | — |  |
| North America | -0.031 | 0.047 | -0.123 | 0.061 | 0.503 |  |
| South America | 0.099 | 0.080 | -0.057 | 0.255 | 0.212 |  |
| Europe | 0.012 | 0.050 | -0.087 | 0.110 | 0.814 |  |
| Oceania | -0.078 | 0.135 | -0.344 | 0.187 | 0.563 |  |
| Stage CKD |  |  |  |  |  | 12.35% |
| Predialysis (Ref.) | — | — | — | — | — |  |
| Dialysis | 0.069 | 0.049 | -0.0.27 | 0.165 | 0.161 |  |
| KTR | -0.145 | 0.077 | -0.296 | 0.005 | 0.058 |  |
| Mixed | -0.124 | 0.062 | -0.245 | -0.004 | 0.043 |  |
| Tool |  |  |  |  |  | 4.21% |
| Fried phenotype (Ref.) | — | — | — | — | — |  |
| CFS | 0.029 | 0.054 | -0.078 | 0.136 | 0.596 |  |
| FRAIL Scale | -0.114 | 0.074 | -0.259 | 0.030 | 0.121 |  |
| TFI | 0.192 | 0.093 | 0.009 | 0.375 | 0.040 |  |
| EFS | -0.126 | 0.132 | -0.385 | 0.133 | 0.339 |  |
| Chinese Frailty Score | 0.260 | 0.129 | 0.007 | 0.513 | 0.044 |  |
| FI | 0.045 | 0.113 | -0.177 | 0.266 | 0.693 |  |
| Other | 0.043 | 0.062 | -0.078 | 0.165 | 0.485 |  |

†Five studies reported age as a categorical variable or did not report.

§One study did not report.

‡Sixty-one studies reported BMI as a categorical variable or did not report.

Table S9 GRADE evidence profile: frailty and prefrail for mortality in patients with CKD

| GRADE evidence assessment (frailty) | | | | | | | | Summary of findings | | | Grading of evidence |
| --- | --- | --- | --- | --- | --- | --- | --- | --- | --- | --- | --- |
| Study design | Rating down factors | | | | | | | Study number | Number of patients | Adjusted HR | ⊕○○○  Very low |
| Observa  -tion studies  (-2) | Risk of bias | Inconsistency | | Indirectness | Imprecision | | Publication bias | 38 | 1,616,758 | 1.941 (95% CI 1.586 to 2.375) |  |
|  | No serious risk of bias (-0) | Serious Inconsistency (-2) | | No serious Indirectness (-0) | No serious Imprecision (-0) | | Suspected (-1) |  |  |  |  |
|  | Rating up factors | | | | | | |  |  |  |  |
|  | Large effect | | Dose response gradient | | | Direction of plausible confounding | |  |  |  |  |
|  | No (+0) | | No (+0) | | | Yes (+1) | |  |  |  |  |
| GRADE evidence assessment (prefrail) | | | | | | | | Summary of findings | | | Grading of evidence |
| Study design | Rating down factors | | | | | | | Study number | Number of patients | Adjusted HR | ⊕○○○  Very low |
| Observa  -tion studies  (-2) | Risk of bias | Inconsistency | | Indirectness | Imprecision | | Publication bias | 12 | 173,297 | 1.345 (95% CI 1.231 to 1.469) |  |
|  | No serious risk of bias (-0) | Serious Inconsistency (-2) | | No serious Indirectness (-0) | No serious Imprecision (-0) | | No serious publication bias (-0) |  |  |  |  |
|  | Rating up factors | | | | | | |  |  |  |  |
|  | Large effect | | Dose response gradient | | | Direction of plausible confounding | |  |  |  |  |
|  | No (+0) | | No (+0) | | | Yes (+1) | |  |  |  |  |

Table S10 Meta-regression for the association of frailty and mortality risk

| Variable | Coefficient | Standard error | Lower 95% CI | Upper 95% CI | *P* | Tau2 |
| --- | --- | --- | --- | --- | --- | --- |
| Mean age (k=37)^†^ | 0.012 | 0.007 | -0.003 | 0.026 | 0.109 | 0.125 |
| Male (k=38) | -0.467 | 0.820 | -2.073 | 1.140 | 0.569 | 0.173 |
| Mean BMI (k=23)^‡^ | 0.037 | 0.049 | -0.059 | 0.132 | 0.450 | 0.239 |
| Geographical location |  |  |  |  |  |  |
| Asia (Ref.) | — | — | — | — | — | 0.048 |
| North America | 0.408 | 0.139 | 0.135 | 0.681 | 0.003 |  |
| Europe | 0.598 | 0.162 | 0.280 | 0.916 | <0.001 |  |
| Stage CKD |  |  |  |  |  |  |
| Predialysis (Ref.) | — | — | — | — | — | 0.095 |
| Dialysis | 0.165 | 0.215 | -0.257 | 0.586 | 0.444 |  |
| KTR | -0.119 | 0.280 | -0.668 | 0.430 | 0.670 |  |
| Mixed | 0.187 | 0.235 | -0.274 | 0.647 | 0.427 |  |

†One studies did not report age.

‡Fifteen studies reported BMI as a categorical variable or did not report.


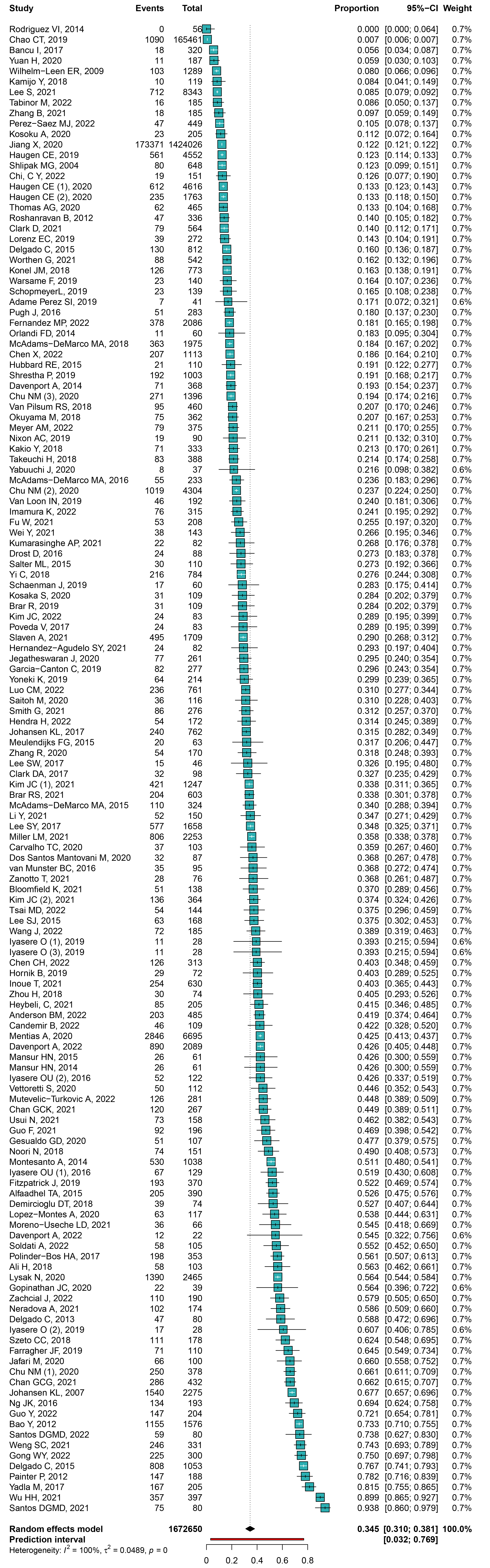
Figure S1 The physical frailty prevalence based on Freeman-Tukey double arcsine transformation


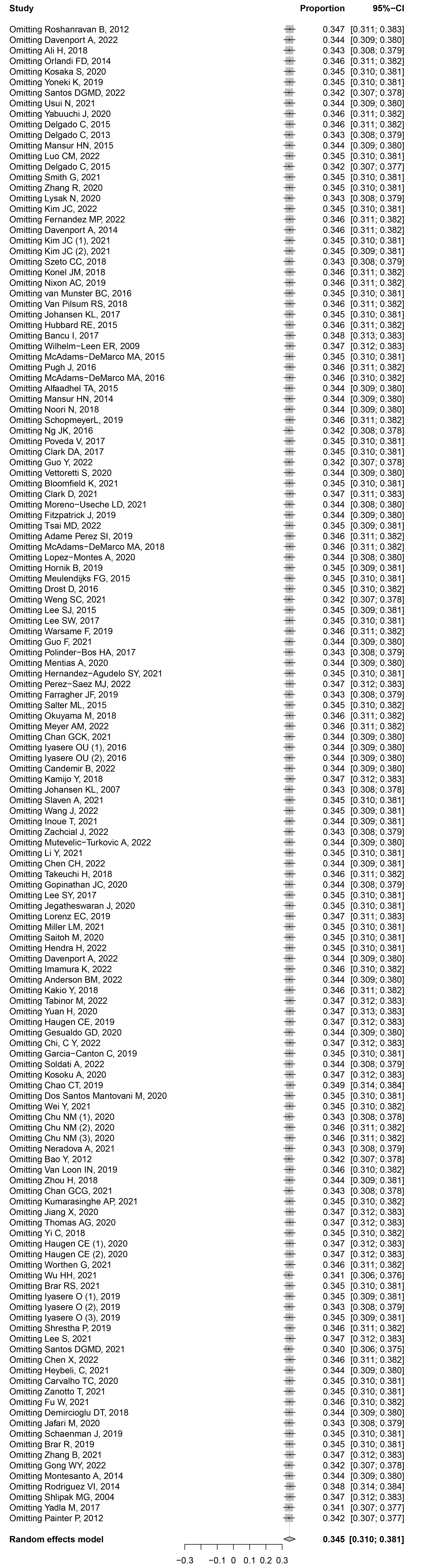
Figure S2 The sensitive analysis for the prevalence of physical frailty based on leave-one-out


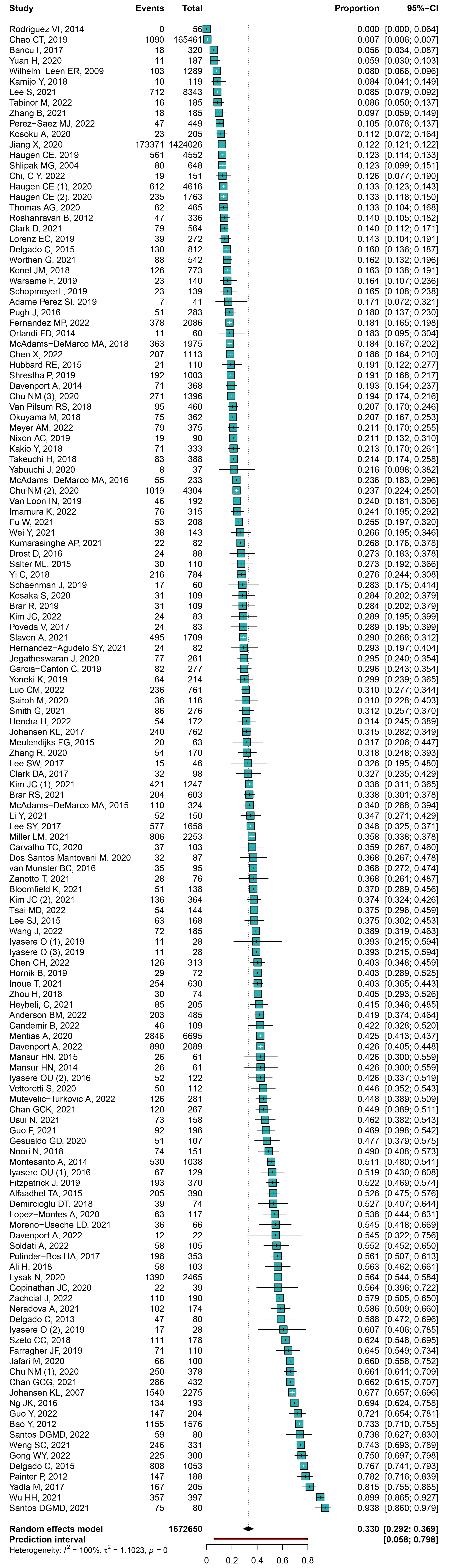
Figure S3 The physical frailty prevalence based on generalized linear mixed model


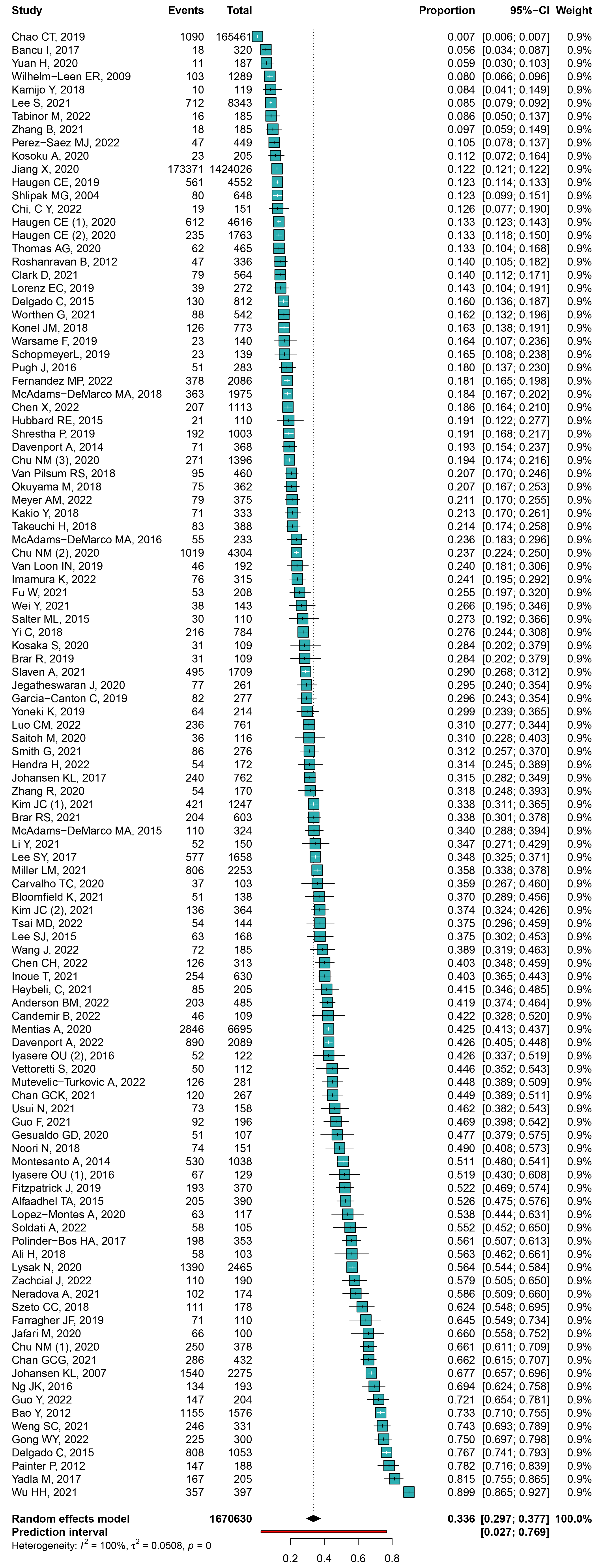
Figure S4 Forest plot of physical frailty prevalence after removing sample size below 100

Figure S5 The prefrail prevalence based on Freeman-Tukey double arcsine transformation


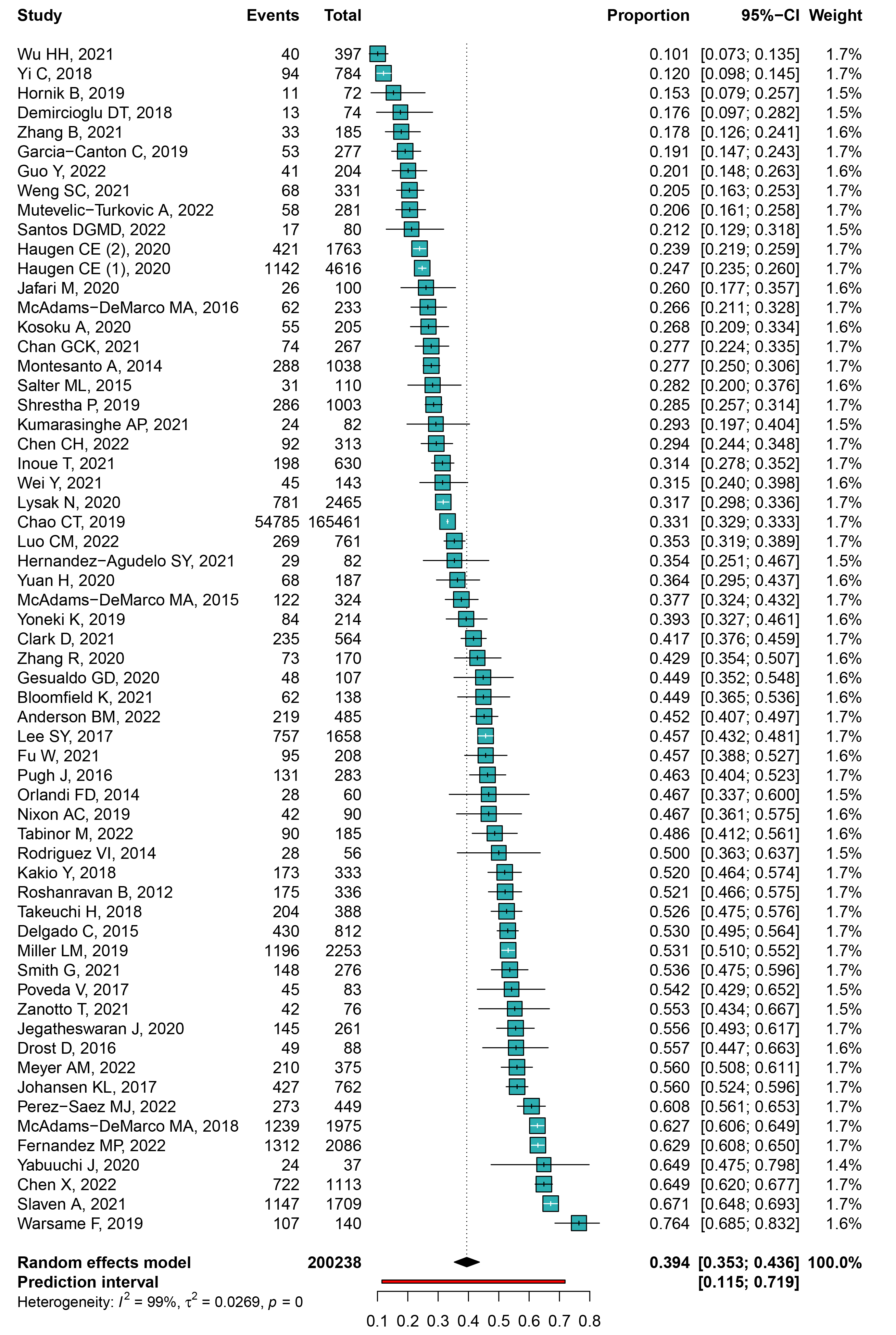


Figure S6 The sensitive analysis for the prevalence of prefrail based on leave-one-out


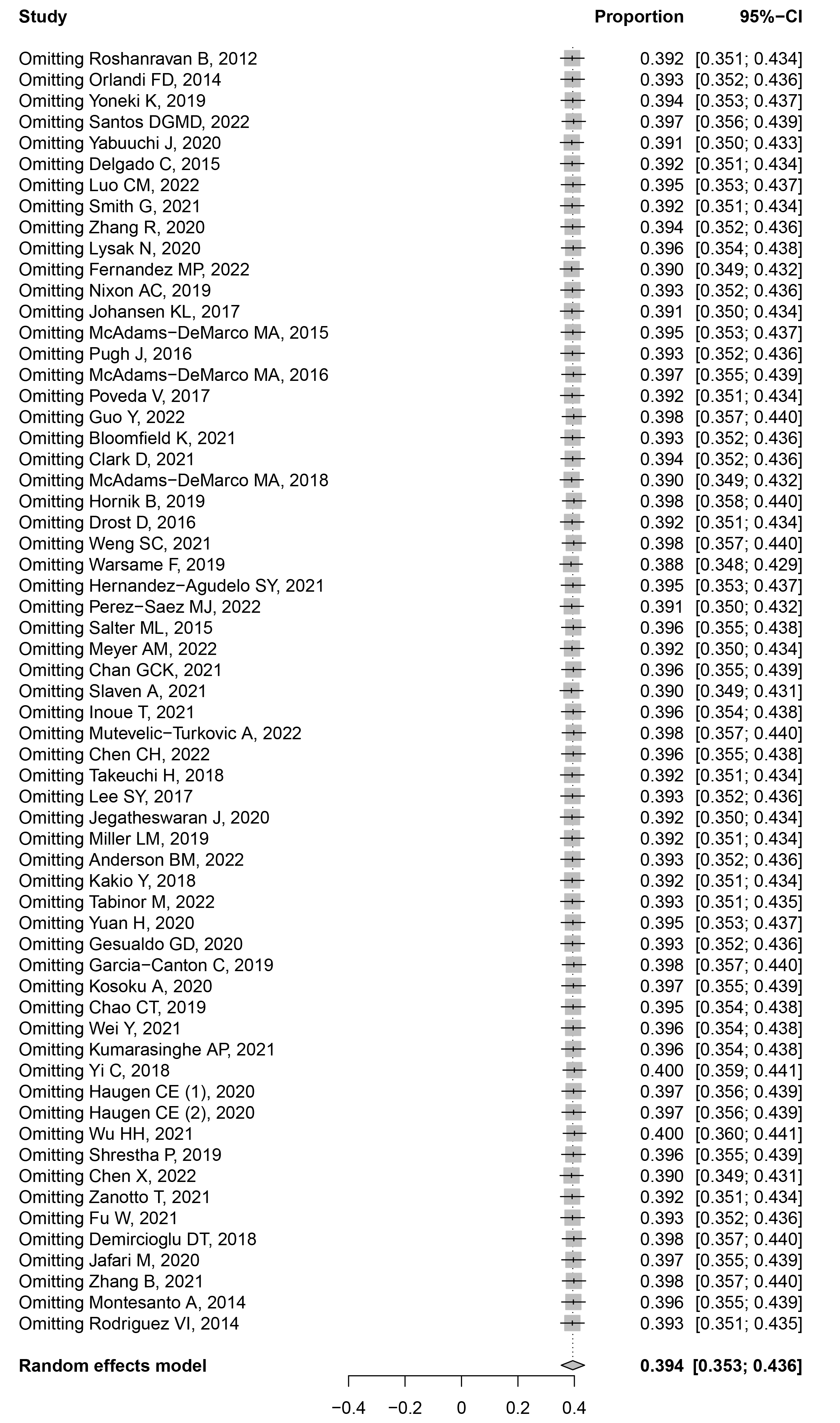


Figure S7 The physical frailty prevalence based on generalized linear mixed model


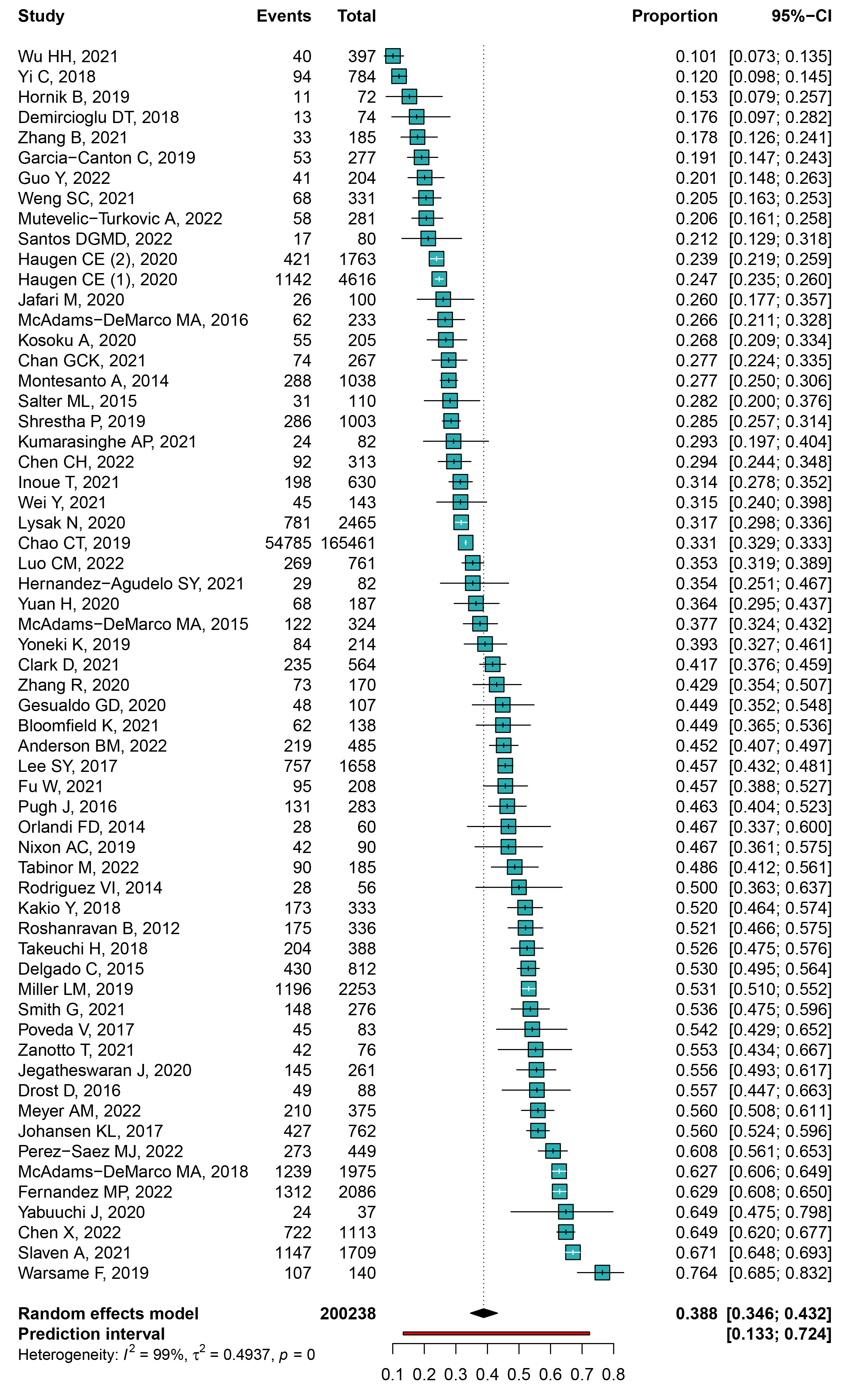


Figure S8 Prevalence of frailty in CKD patients based on country distribution


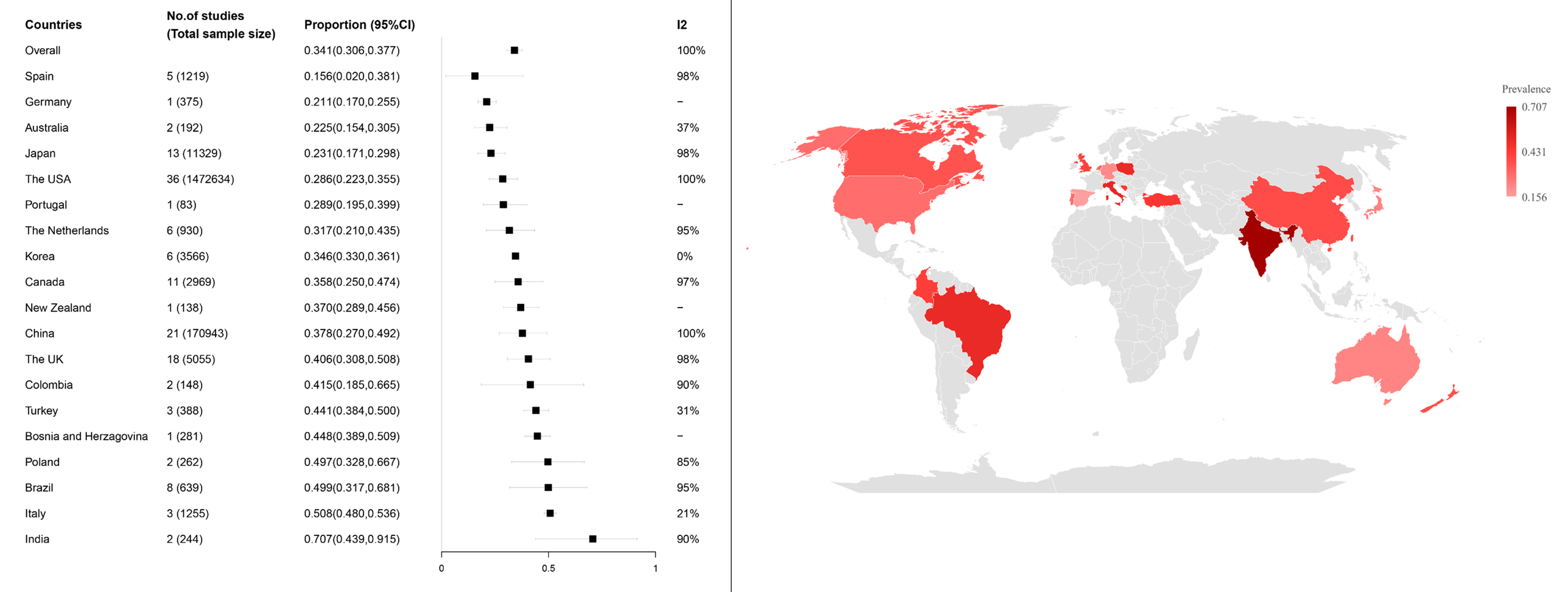


Figure S9 Association between frailty and risk of mortality in patients with CKD


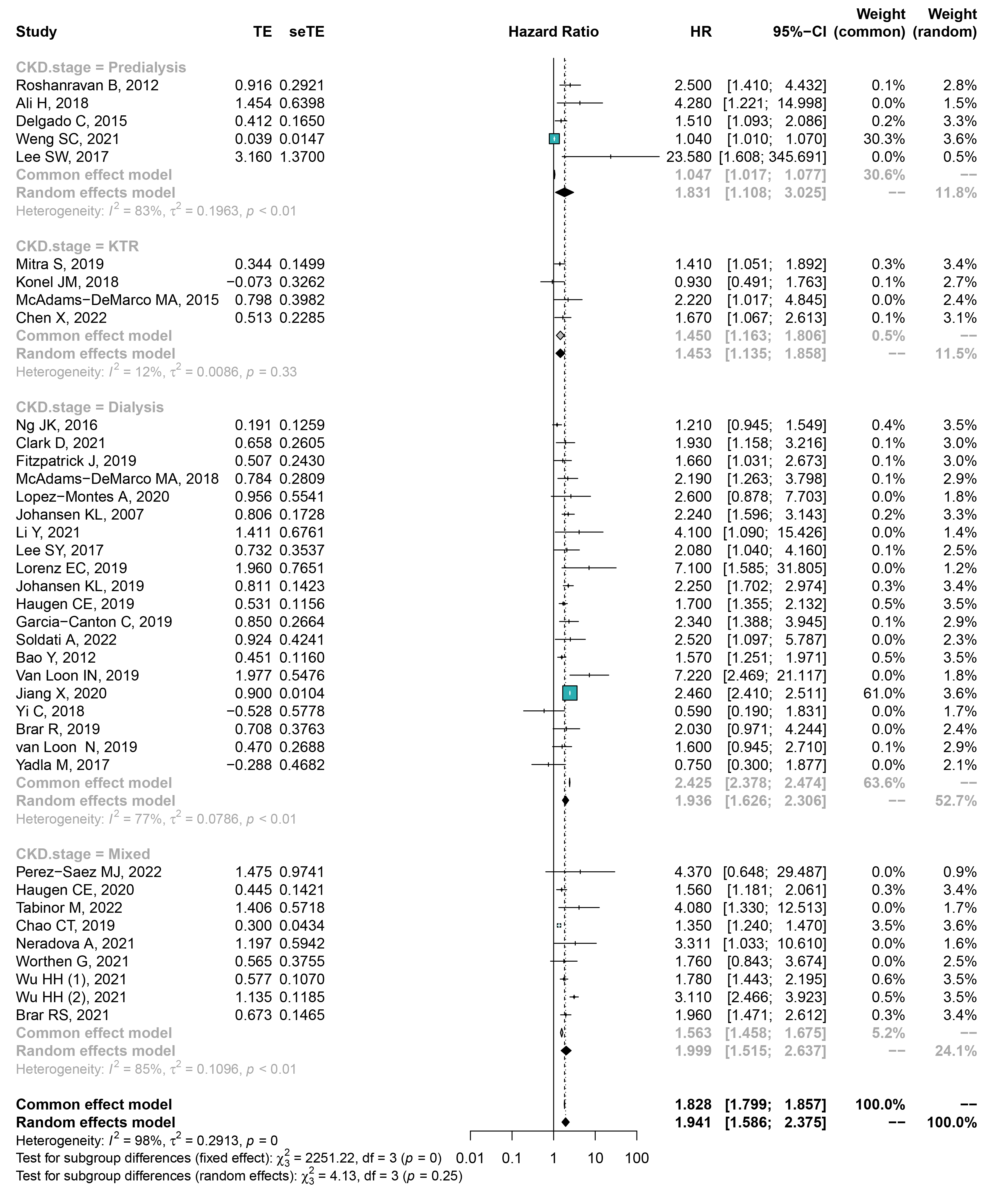


*Note:* The boxes in the forest plot indicate weights, with larger boxes indicating higher weights.

Figure S10 Association between prefrail and risk of mortality in patients with CKD


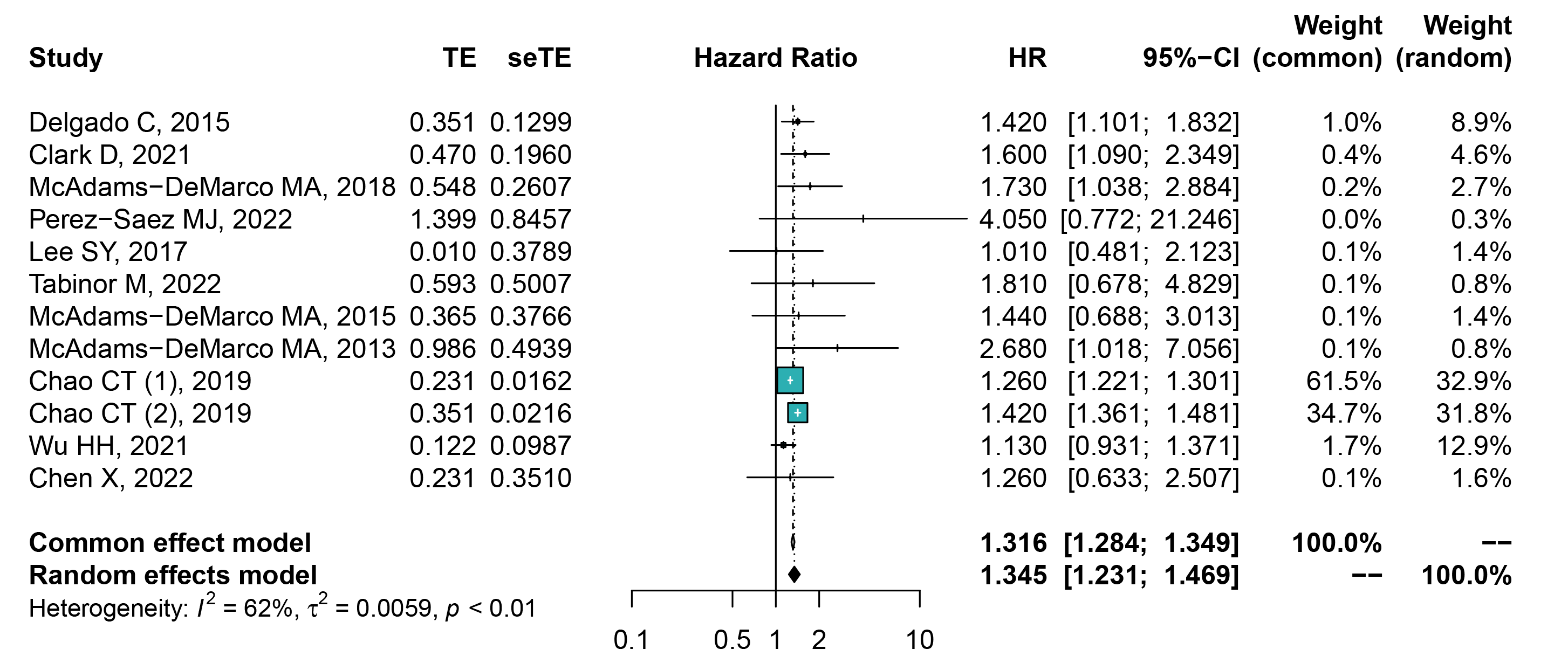


*Note:* The boxes in the forest plot indicate weights, with larger boxes indicating higher weights.

Figure S11 Association of each 1-unit increase in frailty score with the risk of mortality in patients with CKD


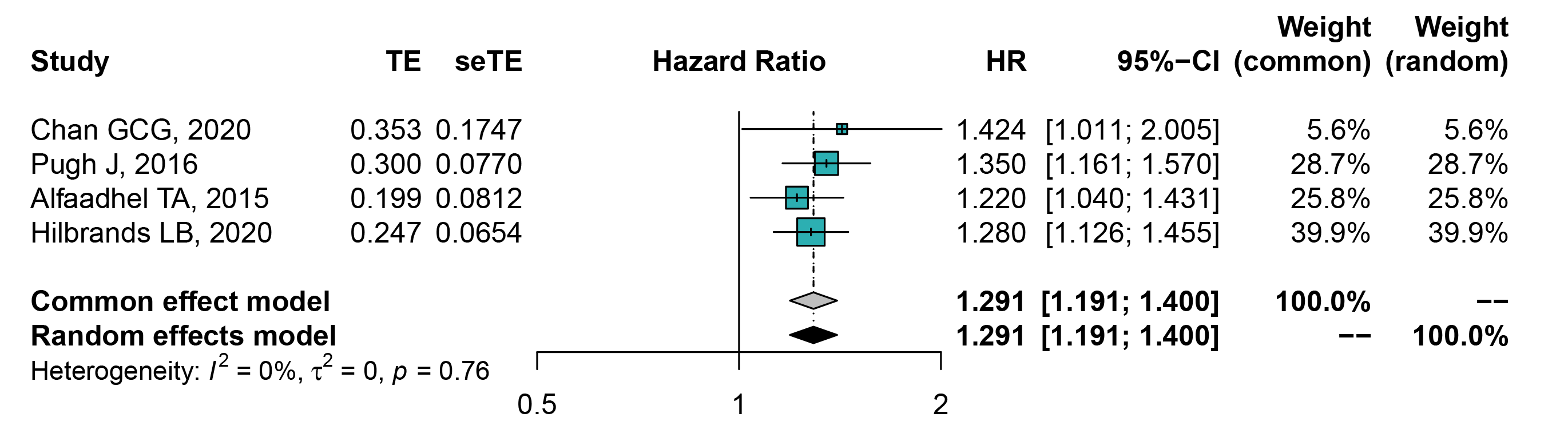


*Note:* The boxes in the forest plot indicate weights, with larger boxes indicating higher weights.

Figure S12 The sensitive analysis for the association between physical frailty and mortality risk based on leave-one-out


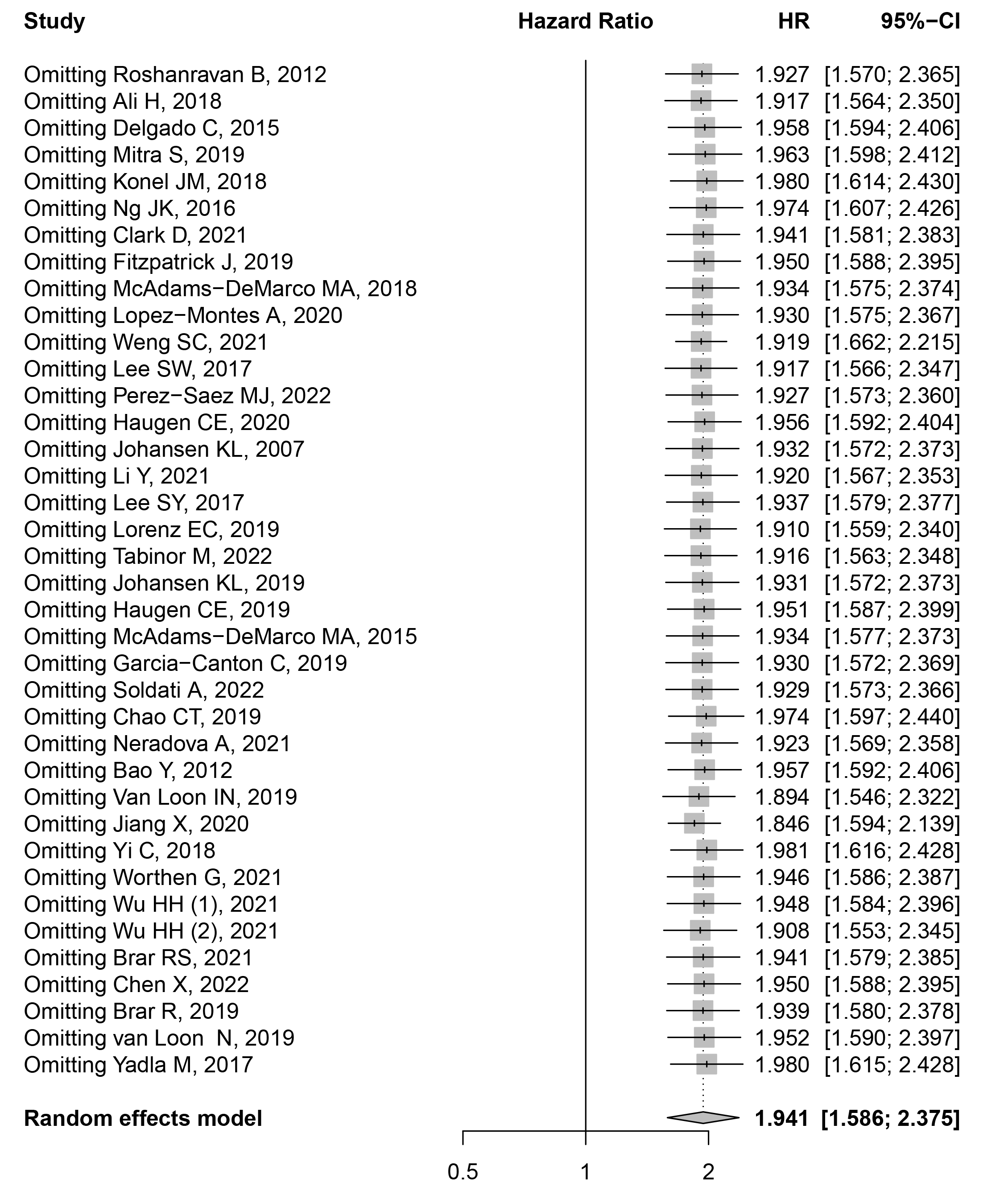


Figure S13 The sensitive analysis for the association between prefrail and mortality risk based on leave-one-out


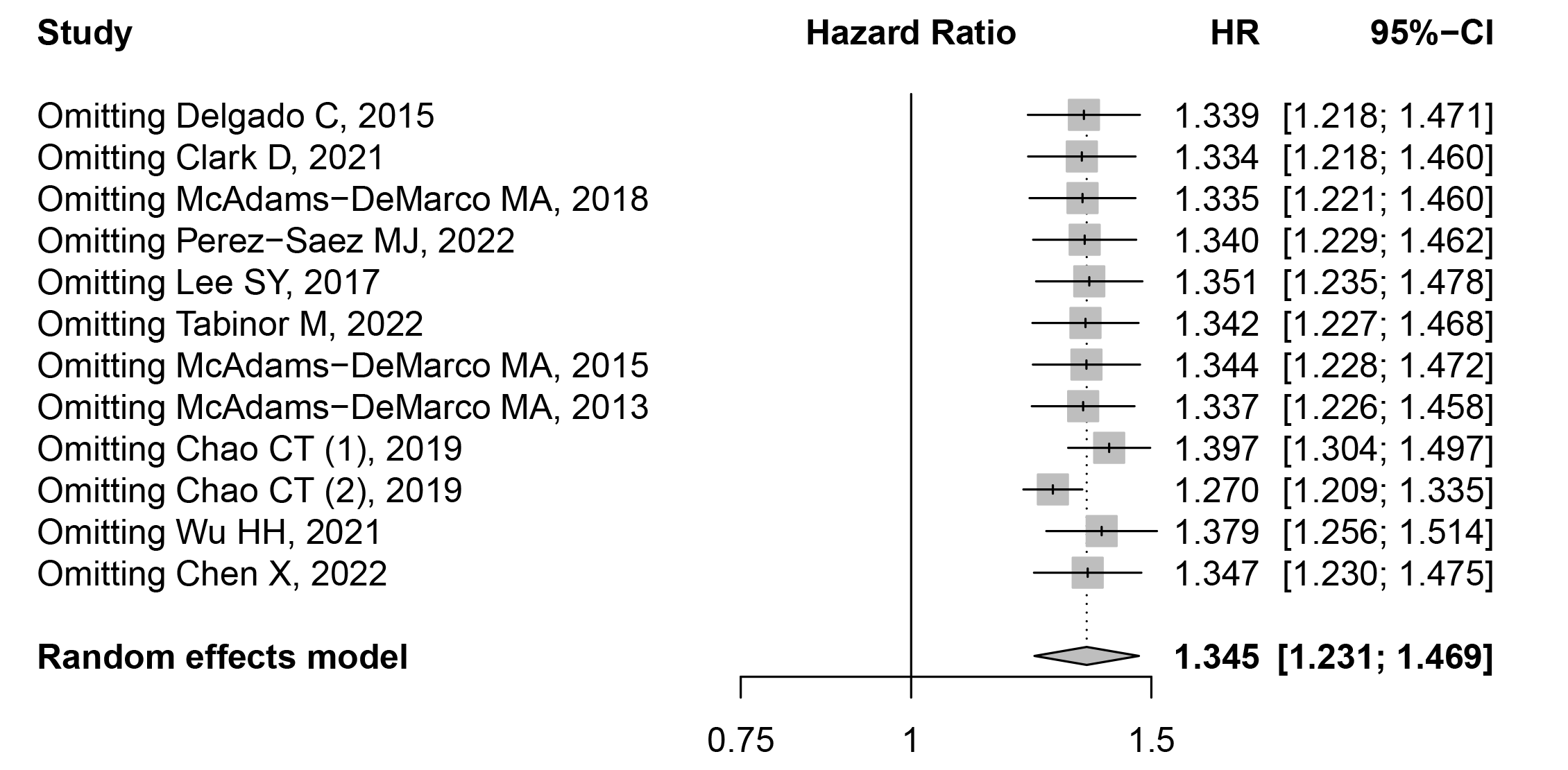


Figure S14 Univariable meta-regression for the prevalence of physical frailty according to study-level characteristics


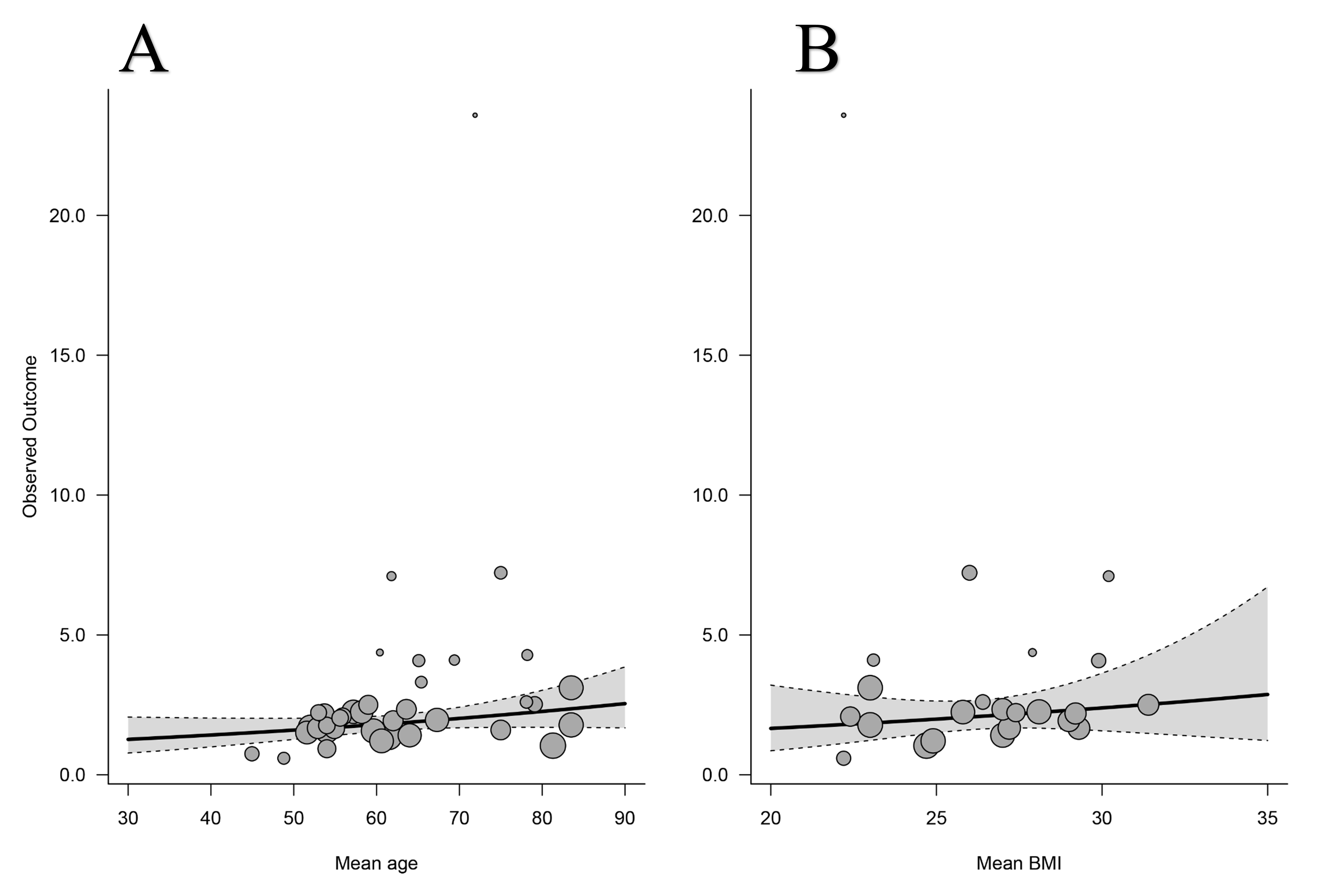


*Note:* Each circle represents one study, and the size of every circle indicates the sample size of each study.

Figure S15 Funnel plots corresponding to the four meta-analyses


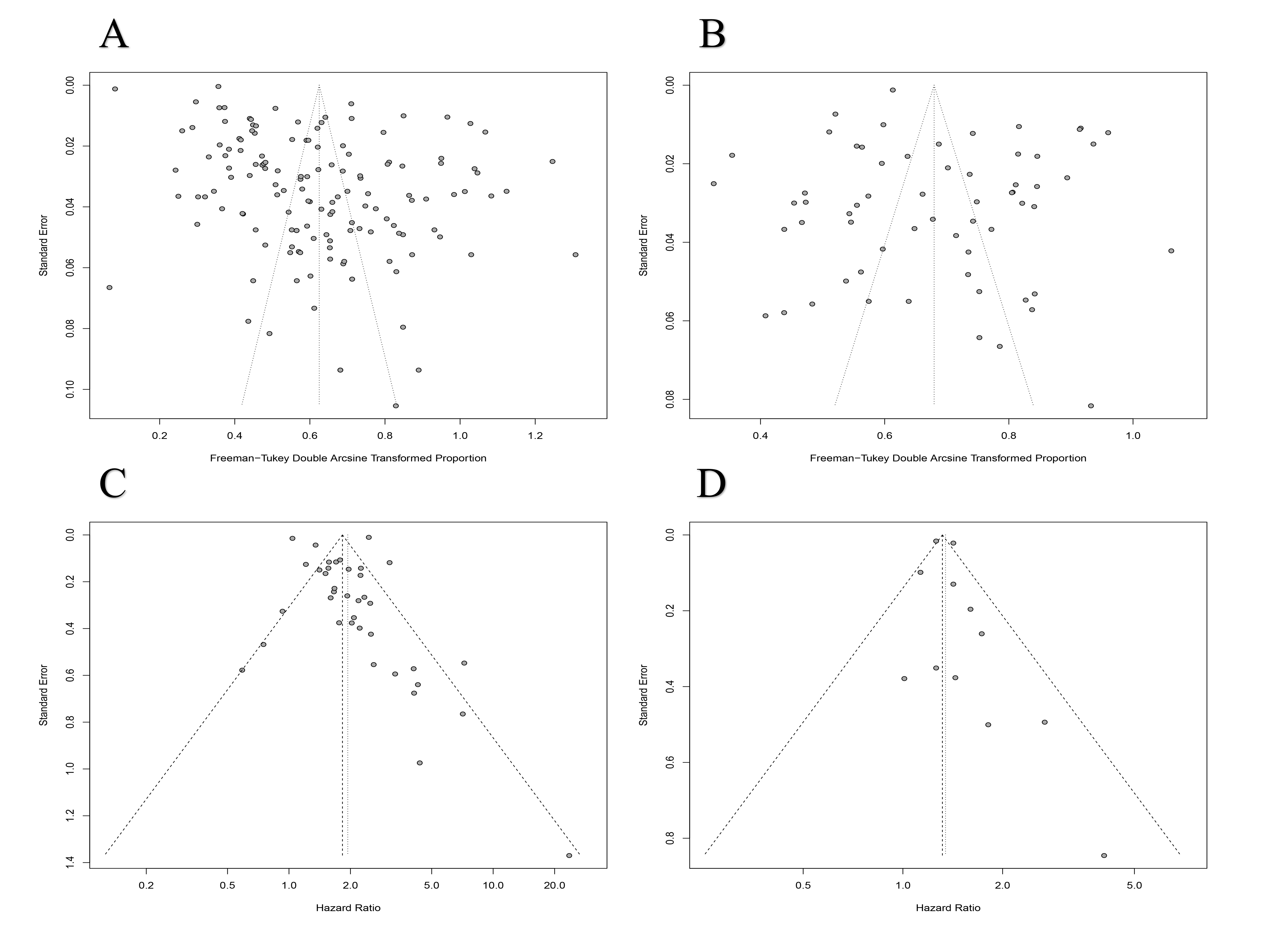


**Legend:** A) the prevalence of frailty (*P* _for egger_: <0.001); B) the prevalence of prefrail (*P* _for egger_: 0.021); C) frailty and the risk of mortality (*P* _for egger_: 0.853); D) prefrail and the risk of mortality (*P* _for egger_: 0.340).

Reference

1. Johansen KL, Dalrymple LS, Delgado C, Chertow GM, Segal MR, Chiang J, Grimes B, Kaysen GA: **Factors Associated with Frailty and Its Trajectory among Patients on Hemodialysis**. *Clin J Am Soc Nephrol* 2017, **12**(7):1100-1108.

2. Brar RS, Whitlock RH, Komenda P, Rigatto C, Prasad B, Bohm C, Tangri N: **Provider Perception of Frailty Is Associated with Dialysis Decision Making in Patients with Advanced CKD**. *Clin J Am Soc Nephrol* 2021, **16**(4):552-559.

3. Chao CT, Wang J, Huang JW, Chan DC, Chien KL: **Frailty Predicts an Increased Risk of End-Stage Renal Disease with Risk Competition by Mortality among 165,461 Diabetic Kidney Disease Patients**. *Aging Dis* 2019, **10**(6):1270-1281.

4. Slaven A, Hsu J, Schelling JR, Navaneethan SD, Rincon-Choles H, McAdams-DeMarco MA, Schachere M, O'Malley N, Deluca J, Lustigova E *et al*: **Social Support in Older Adults With CKD: A Report From the CRIC (Chronic Renal Insufficiency Cohort) Study**. *Kidney Med* 2021, **3**(5):776-784.e771.

5. Chan G, Ng J, Chow KM, Kwong V, Pang WF, Cheng P, Law MC, Leung CB, Li P, Szeto CC: **Progression in Physical Frailty in Peritoneal Dialysis Patients**. *Kidney Blood Press Res* 2021, **46**(3):342-351.

6. Haugen CE, Chu NM, Ying H, Warsame F, Holscher CM, Desai NM, Jones MR, Norman SP, Brennan DC, Garonzik-Wang J *et al*: **Frailty and Access to Kidney Transplantation**. *Clin J Am Soc Nephrol* 2019, **14**(4):576-582.

7. Chu NM, Chen X, Norman SP, Fitzpatrick J, Sozio SM, Jaar BG, Frey A, Estrella MM, Xue QL, Parekh RS *et al*: **Frailty Prevalence in Younger End-Stage Kidney Disease Patients Undergoing Dialysis and Transplantation**. *Am J Nephrol* 2020, **51**(7):501-510.

8. Chen X, Shafaat O, Liu Y, King EA, Weiss CR, Xue QL, Walston JD, Segev DL, McAdams-DeMarco MA: **Revision of frailty assessment in kidney transplant recipients: Replacing unintentional weight loss with CT-assessed sarcopenia in the physical frailty phenotype**. *Am J Transplant* 2022, **22**(4):1145-1157.

9. Van Loon IN, Goto NA, Boereboom FTJ, Bots ML, Hoogeveen EK, Gamadia L, Van Bommel EFH, Van De Ven PJG, Douma CE, Vincent HH *et al*: **Geriatric Assessment and the Relation with Mortality and Hospitalizations in Older Patients Starting Dialysis**. *Nephron* 2019, **143**(2):108-119.

10. Lee SY, Yang DH, Hwang E, Kang SH, Park SH, Kim TW, Lee DH, Park K, Kim JC: **The Prevalence, Association, and Clinical Outcomes of Frailty in Maintenance Dialysis Patients**. *Journal of renal nutrition : the official journal of the Council on Renal Nutrition of the National Kidney Foundation* 2017, **27**(2):106-112.

11. Zanotto T, Mercer TH, van der Linden ML, Koufaki P: **Screening tools to expedite assessment of frailty in people receiving haemodialysis: a diagnostic accuracy study**. *BMC Geriatr* 2021, **21**(1):411.

12. Pérez-Sáez MJ, Redondo-Pachón D, Arias-Cabrales CE, Faura A, Bach A, Buxeda A, Burballa C, Junyent E, Crespo M, Marco E *et al*: **Outcomes of Frail Patients While Waiting for Kidney Transplantation: Differences between Physical Frailty Phenotype and FRAIL Scale**. *J Clin Med* 2022, **11**(3).

13. Nixon AC, Bampouras TM, Pendleton N, Mitra S, Dhaygude AP: **Diagnostic Accuracy of Frailty Screening Methods in Advanced Chronic Kidney Disease**. *NEPHRON* 2019, **141**(3):147-155.

14. Roshanravan B, Khatri M, Robinson-Cohen C, Levin G, Patel KV, de Boer IH, Seliger S, Ruzinski J, Himmelfarb J, Kestenbaum B: **A prospective study of frailty in nephrology-referred patients with CKD**. *Am J Kidney Dis* 2012, **60**(6):912-921.

15. Davenport A: **Application of the Clinical Frailty Score and body composition and upper arm strength in haemodialysis patients**. *CLINICAL KIDNEY JOURNAL* 2022, **15**(3):553-559.

16. Ali H, Abdelaziz T, Abdelaal F, Baharani J: **Assessment of prevalence and clinical outcome of frailty in an elderly predialysis cohort using simple tools**. *Saudi journal of kidney diseases and transplantation : an official publication of the Saudi Center for Organ Transplantation, Saudi Arabia* 2018, **29**(1):63-70.

17. Orlandi FD, Gesualdo GD: **Assessment of the frailty level of elderly people with chronic kidney disease undergoing hemodialysis**. *ACTA PAULISTA DE ENFERMAGEM* 2014, **27**(1):29-34.

18. Kosaka S, Ohara Y, Naito S, Iimori S, Kado H, Hatta T, Yanishi M, Uchida S, Tanaka M: **Association among kidney function, frailty, and oral function in patients with chronic kidney disease: a cross-sectional study**. *BMC NEPHROLOGY* 2020, **21**(1).

19. Yoneki K, Kitagawa J, Hoshi K, Harada M, Watanabe T, Shimoda T, Matsuzawa R, Yoshida A, Matsunaga Y, Takeuchi Y *et al*: **Association between frailty and bone loss in patients undergoing maintenance hemodialysis**. *JOURNAL OF BONE AND MINERAL METABOLISM* 2019, **37**(1):81-89.

20. Santos D, Ferreira L, Pallone JM, Ottaviani AC, Santos-Orlandi AA, Pavarini S, Zazzetta MS, Orlandi FS: **Association between frailty and depression among hemodialysis patients: a cross-sectional study**. *Sao Paulo Med J* 2022.

21. Usui N, Yokoyama M, Nakata J, Suzuki Y, Tsubaki A, Kojima S, Inatsu A, Hisadome H, Uehata A: **Association between social frailty as well as early physical dysfunction and exercise intolerance among older patients receiving hemodialysis**. *Geriatr Gerontol Int* 2021, **21**(8):664-669.

22. Yabuuchi J, Ueda S, Yamagishi SI, Nohara N, Nagasawa H, Wakabayashi K, Matsui T, Yuichiro H, Kadoguchi T, Otsuka T *et al*: **Association of advanced glycation end products with sarcopenia and frailty in chronic kidney disease**. *Sci Rep* 2020, **10**(1):17647.

23. Delgado C, Grimes BA, Glidden DV, Shlipak M, Sarnak MJ, Johansen KL: **Association of Frailty based on self-reported physical function with directly measured kidney function and mortality**. *BMC NEPHROLOGY* 2015, **16**.

24. Delgado C, Doyle JW, Johansen KL: **Association of Frailty With Body Composition Among Patients on Hemodialysis**. *JOURNAL OF RENAL NUTRITION* 2013, **23**(5):356-362.

25. Mansur HN, Lovisi JCM, Colugnati FAB, Raposo NRB, Da Silva Fernandes NM, Bastos MG: **Association of frailty with endothelial dysfunction and its possible impact on negative outcomes in Brazilian predialysis patients with chronic kidney disease**. *BMC NEPHROLOGY* 2015, **16**(1).

26. Luo CM, Hsieh MY, Cheng CH, Chen CH, Liao MT, Chuang SY, Wu CC: **Association of Frailty With Thrombosis of Hemodialysis Vascular Access: A Prospective Taiwanese Cohort Study**. *Am J Kidney Dis* 2022.

27. Delgado C, Shieh S, Grimes B, Chertow GM, Dalrymple LS, Kaysen GA, Kornak J, Johansen KL: **Association of Self-Reported Frailty with Falls and Fractures among Patients New to Dialysis**. *Am J Nephrol* 2015, **42**(2):134-140.

28. Smith G, Avenell A, Band MM, Hampson G, Lamb EJ, Littleford RC, McNamee P, Soiza RL, Sumukadas D, Witham MD: **Associations between frailty, physical performance, and renal biomarkers in older people with advanced chronic kidney disease**. *EUROPEAN GERIATRIC MEDICINE* 2021, **12**(5):943-952.

29. Zhang R, Pu C, Cui X, Zhang N, Li X, Zheng F: **Burden in primary family caregivers caring for uremic patients on maintenance peritoneal dialysis**. *Perit Dial Int* 2020, **40**(6):556-562.

30. Lysak N, Hashemighouchani H, Davoudi A, Pourafshar N, Loftus TJ, Ruppert M, Efron PA, Rashidi P, Bihorac A, Ozrazgat-Baslanti T: **Cardiovascular death and progression to end-stage renal disease after major surgery in elderly patients**. *BJS OPEN* 2020, **4**(1):145-156.

31. Kim JC, Do JY, Kang SH: **Clinical Significance of Volume Status in Body Composition and Physical Performance Measurements in Hemodialysis Patients**. *FRONTIERS IN NUTRITION* 2022, **9**.

32. Mitra S, Jayanti A, Vart P, Coca A, Gallieni M, Øvrehus MA, Midtvedt K, Abd Elhafeez S, Gandolfini I, Büttner S *et al*: **Clinical triage of patients on kidney replacement therapy presenting with COVID-19: An ERACODA registry analysis**. *NEPHROLOGY DIALYSIS TRANSPLANTATION* 2021, **36**(12):2308-2320.

33. Fernandez MP, Miguel PM, Ying H, Haugen CE, Chu NM, Puyol D, Rodriguez-Manas L, Norman SP, Walston JD, Segev DL *et al*: **Comorbidity, Frailty, and Waitlist Mortality among Kidney Transplant Candidates of All Ages**. *AMERICAN JOURNAL OF NEPHROLOGY* 2019, **49**(2):103-110.

34. Davenport A: **Comparison of frailty, sarcopenia and protein energy wasting in a contemporary peritoneal dialysis cohort**. *Peritoneal Dial Int* 2022.

35. Kim JC, Do JY, Kang SH: **Comparisons of physical activity and understanding of the importance of exercise according to dialysis modality in maintenance dialysis patients**. *SCIENTIFIC REPORTS* 2021, **11**(1).

36. Szeto CC, Chan GC, Ng JK, Chow KM, Kwan BC, Cheng PM, Kwong VW, Law MC, Leung CB, Li PK: **Depression and Physical Frailty Have Additive Effect on the Nutritional Status and Clinical Outcome of Chinese Peritoneal Dialysis**. *Kidney Blood Press Res* 2018, **43**(3):914-923.

37. Konel JM, Warsame F, Ying H, Haugen CE, Mountford A, Chu NM, Crews DC, Desai NM, Garonzik-Wang JM, Walston JD *et al*: **Depressive symptoms, frailty, and adverse outcomes among kidney transplant recipients**. *Clin Transplant* 2018, **32**(10):e13391.

38. van Munster BC, Drost D, Kalf A, Vogtlander NP: **Discriminative value of frailty screening instruments in end-stage renal disease**. *Clin Kidney J* 2016, **9**(4):606-610.

39. Van Pilsum RS, Konel J, Warsame F, Ying H, Buta B, Haugen C, King E, DiBrito S, Varadhan R, Rodríguez-Mañas L *et al*: **Engaging clinicians and patients to assess and improve frailty measurement in adults with end stage renal disease**. *BMC Nephrol* 2018, **19**(1):8.

40. Hubbard RE, Peel NM, Smith M, Dawson B, Lambat Z, Bak M, Best J, Johnson DW: **Feasibility and construct validity of a Frailty index for patients with chronic kidney disease**. *AUSTRALASIAN JOURNAL ON AGEING* 2015, **34**(3):E9-E12.

41. Bancu I, Graterol F, Bonal J, Fernández-Crespo P, Garcia J, Aguerrevere S, Del CD, Bonet J: **Frail Patient in Hemodialysis: A New Challenge in Nephrology-Incidence in Our Area, Barcelonès Nord and Maresme**. *J Aging Res* 2017, **2017**:7624139.

42. Wilhelm-Leen ER, Hall YN, Tamura MK, Chertow GM: **Frailty and Chronic Kidney Disease: The Third National Health and Nutrition Evaluation Survey**. *AMERICAN JOURNAL OF MEDICINE* 2009, **122**(7):664-U686.

43. McAdams-DeMarco MA, Tan J, Salter ML, Gross A, Meoni LA, Jaar BG, Kao WH, Parekh RS, Segev DL, Sozio SM: **Frailty and Cognitive Function in Incident Hemodialysis Patients**. *Clin J Am Soc Nephrol* 2015, **10**(12):2181-2189.

44. Pugh J, Aggett J, Goodland A, Prichard A, Thomas N, Donovan K, Roberts G: **Frailty and comorbidity are independent predictors of outcome in patients referred for pre-dialysis education**. *Clin Kidney J* 2016, **9**(2):324-329.

45. McAdams-DeMarco MA, Ying H, Olorundare I, King EA, Desai N, Dagher N, Lonze B, Montgomery R, Walston J, Segev DL: **Frailty and Health-Related Quality of Life in End Stage Renal Disease Patients of All Ages**. *J Frailty Aging* 2016, **5**(3):174-179.

46. Alfaadhel TA, Soroka SD, Kiberd BA, Landry D, Moorhouse P, Tennankore KK: **Frailty and mortality in dialysis: evaluation of a clinical frailty scale**. *Clin J Am Soc Nephrol* 2015, **10**(5):832-840.

47. Mansur HN, Colugnati FA, Grincenkov FR, Bastos MG: **Frailty and quality of life: a cross-sectional study of Brazilian patients with pre-dialysis chronic kidney disease**. *Health Qual Life Outcomes* 2014, **12**:27.

48. Noori N, Sharma PA, Lakhani R, Janes S, Goldstein MB: **Frailty and the Quality of Life in Hemodialysis Patients: The Importance of Waist Circumference**. *J Ren Nutr* 2018, **28**(2):101-109.

49. Schopmeyer L, El MM, Nieuwenhuijs-Moeke GJ, Berger SP, Bakker S, Pol RA: **Frailty has a significant influence on postoperative complications after kidney transplantation-a prospective study on short-term outcomes**. *Transpl Int* 2019, **32**(1):66-74.

50. Ng JK, Kwan BC, Chow KM, Cheng PM, Law MC, Pang WF, Leung CB, Li PK, Szeto CC: **Frailty in Chinese Peritoneal Dialysis Patients: Prevalence and Prognostic Significance**. *Kidney Blood Press Res* 2016, **41**(6):736-745.

51. Poveda V, Filgueiras M, Miranda V, Santos-Silva A, Paúl C, Costa E: **Frailty in End-Stage Renal Disease Patients under Dialysis and Its Association with Clinical and Biochemical Markers**. *J Frailty Aging* 2017, **6**(2):103-106.

52. Clark DA, Khan U, Kiberd BA, Turner CC, Dixon A, Landry D, Moffatt HC, Moorhouse PA, Tennankore KK: **Frailty in end-stage renal disease: comparing patient, caregiver, and clinician perspectives**. *BMC Nephrol* 2017, **18**(1):148.

53. Guo Y, Tian R, Ye P, Luo Y: **Frailty in Older Patients Undergoing Hemodialysis and Its Association with All-Cause Mortality: A Prospective Cohort Study**. *Clin Interv Aging* 2022, **17**:265-275.

54. Vettoretti S, Caldiroli L, Porata G, Vezza C, Cesari M, Messa P: **Frailty phenotype and multi-domain impairments in older patients with chronic kidney disease**. *BMC GERIATRICS* 2020, **20**(1).

55. Bloomfield K, Wu Z, Chan L, de Zoysa JR: **Frailty prevalence in Aotearoa New Zealand haemodialysis patients and its association with hospitalisations**. *N Z Med J* 2021, **134**(1546):95-108.

56. Clark D, Matheson K, West B, Vinson A, West K, Jain A, Rockwood K, Tennankore K: **Frailty Severity and Hospitalization After Dialysis Initiation**. *Can J Kidney Health Dis* 2021, **8**.

57. Moreno-Useche LD, Urrego-Rubio J, Cadena-Sanabria M, Amaya RR, Maldonado-Navas S, Ruiz-González C: **Frailty syndrome in patients with chronic kidney disease at a dialysis centre from santander, colombia**. *J Gerontology Geriatrics* 2021, **69**(2):103-109.

58. Fitzpatrick J, Sozio SM, Jaar BG, Estrella MM, Segev DL, Parekh RS, McAdams-DeMarco MA: **Frailty, body composition and the risk of mortality in incident hemodialysis patients: the Predictors of Arrhythmic and Cardiovascular Risk in End Stage Renal Disease study**. *Nephrol Dial Transplant* 2019, **34**(2):346-354.

59. Tsai MD, Tsai JP, Chen ML, Chang LC: **Frailty, Health Literacy, and Self-Care in Patients with Chronic Kidney Disease in Taiwan**. *International Journal of Environmental Research and Public Health* 2022, **19**(9).

60. Adame PS, Senior PA, Field CJ, Jindal K, Mager DR: **Frailty, Health-Related Quality of Life, Cognition, Depression, Vitamin D and Health-Care Utilization in an Ambulatory Adult Population With Type 1 or Type 2 Diabetes Mellitus and Chronic Kidney Disease: A Cross-Sectional Analysis**. *Can J Diabetes* 2019, **43**(2):90-97.

61. McAdams-DeMarco MA, Ying H, Thomas AG, Warsame F, Shaffer AA, Haugen CE, Garonzik-Wang JM, Desai NM, Varadhan R, Walston J *et al*: **Frailty, Inflammatory Markers, and Waitlist Mortality Among Patients With End-stage Renal Disease in a Prospective Cohort Study**. *TRANSPLANTATION* 2018, **102**(10):1740-1746.

62. López-Montes A, Martínez-Villaescusa M, Pérez-Rodríguez A, Andrés-Monpeán E, Martínez-Díaz M, Masiá J, Giménez-Bachs JM, Abizanda P: **Frailty, physical function and affective status in elderly patients on hemodialysis**. *Arch Gerontol Geriatr* 2020, **87**:103976.

63. Hornik B, Dulawa J: **Frailty, Quality of Life, Anxiety, and Other Factors Affecting Adherence to Physical Activity Recommendations by Hemodialysis Patients**. *INTERNATIONAL JOURNAL OF ENVIRONMENTAL RESEARCH AND PUBLIC HEALTH* 2019, **16**(10).

64. Meulendijks FG, Hamaker ME, Boereboom FT, Kalf A, Vögtlander NP, van Munster BC: **Groningen frailty indicator in older patients with end-stage renal disease**. *Ren Fail* 2015, **37**(9):1419-1424.

65. Drost D, Kalf A, Vogtlander N, van Munster BC: **High prevalence of frailty in end-stage renal disease**. *Int Urol Nephrol* 2016, **48**(8):1357-1362.

66. Weng SC, Chen YC, Hsu CY, Lin CS, Tarng DC, Lin SY: **Impacts of Heart Failure and Physical Performance on Long-Term Mortality in Old Patients With Chronic Kidney Disease**. *FRONTIERS IN CARDIOVASCULAR MEDICINE* 2021, **8**.

67. Lee SJ, Son H, Shin SK: **Influence of frailty on health-related quality of life in pre-dialysis patients with chronic kidney disease in Korea: a cross-sectional study**. *Health Qual Life Outcomes* 2015, **13**:70.

68. Lee SW, Lee A, Yu MY, Kim SW, Kim KI, Na KY, Chae DW, Kim CH, Chin HJ: **Is Frailty a Modifiable Risk Factor of Future Adverse Outcomes in Elderly Patients with Incident End-Stage Renal Disease?** *JOURNAL OF KOREAN MEDICAL SCIENCE* 2017, **32**(11):1800-+.

69. Warsame F, Haugen CE, Ying H, Garonzik-Wang JM, Desai NM, Hall RK, Kambhampati R, Crews DC, Purnell TS, Segev DL *et al*: **Limited health literacy and adverse outcomes among kidney transplant candidates**. *Am J Transplant* 2019, **19**(2):457-465.

70. Guo F, Xu J, Kang Y, Zhou H: **Logistic regression analysis of frailty phenotype in the young and the middleaged maintenance hemodialysis patients**. *Acta Med Mediterr* 2021, **37**(6):3577-3582.

71. Polinder-Bos HA, Nacak H, Dekker FW, Bakker S, Gaillard C, Gansevoort RT: **Low Urinary Creatinine Excretion Is Associated With Self-Reported Frailty in Patients With Advanced Chronic Kidney Disease**. *KIDNEY INTERNATIONAL REPORTS* 2017, **2**(4):676-685.

72. Mentias A, Desai MY, Saad M, Horwitz PA, Rossen JD, Panaich S, Jneid H, Kapadia S, Vaughan-Sarrazin M: **Management of Aortic Stenosis in Patients With End-Stage Renal Disease on Hemodialysis**. *CIRCULATION-CARDIOVASCULAR INTERVENTIONS* 2020, **13**(8).

73. Hernandez-Agudelo SY, Musso CG, González-Torres HJ, Castro-Hernández C, Maya-Altamiranda LP, Quintero-Cruz MV, Corradino C, Terrasa SA, Aroca-Martínez GJ, Cadena-Bonfanti A: **Optimizing dialysis dose in the context of frailty: an exploratory study**. *Int Urol Nephrol* 2021, **53**(5):1025-1031.

74. Farragher JF, Oliver MJ, Jain AK, Flanagan S, Koyle K, Jassal SV: **PD Assistance and Relationship to Co-Existing Geriatric Syndromes in Incident Peritoneal Dialysis Therapy Patients**. *Perit Dial Int* 2019, **39**(4):375-381.

75. Salter ML, Gupta N, Massie AB, McAdams-DeMarco MA, Law AH, Jacob RL, Gimenez LF, Jaar BG, Walston JD, Segev DL: **Perceived frailty and measured frailty among adults undergoing hemodialysis: a cross-sectional analysis**. *BMC Geriatr* 2015, **15**:52.

76. Okuyama M, Takeuchi H, Uchida HA, Kakio Y, Okuyama Y, Umebayashi R, Wada K, Sugiyama H, Sugimoto K, Rakugi H *et al*: **Peripheral artery disease is associated with frailty in chronic hemodialysis patients**. *VASCULAR* 2018, **26**(4):425-431.

77. Meyer AM, Pickert L, Heeß A, Becker I, Kurschat C, Bartram MP, Benzing T, Polidori MC: **Prognostic Signature of Chronic Kidney Disease in Advanced Age: Secondary Analysis from the InGAH Study with One‐Year Follow‐Up**. *Biomolecules* 2022, **12**(3).

78. Iyasere OU, Brown EA, Johansson L, Huson L, Smee J, Maxwell AP, Farrington K, Davenport A: **Quality of Life and Physical Function in Older Patients on Dialysis: A Comparison of Assisted Peritoneal Dialysis with Hemodialysis**. *Clin J Am Soc Nephrol* 2016, **11**(3):423-430.

79. Candemir B, Yıldırım F, Yaşar E, Erten Y, Göker B: **Relationship between Health Literacy and Frailty in Older Adults with Chronic Kidney Disease**. *Exp Aging Res* 2022:1-13.

80. Kamijo Y, Kanda E, Ishibashi Y, Yoshida M: **Sarcopenia and Frailty in PD: Impact on Mortality, Malnutrition, and Inflammation**. *Perit Dial Int* 2018, **38**(6):447-454.

81. Johansen KL, Chertow GM, Jin C, Kutner NG: **Significance of frailty among dialysis patients**. *J Am Soc Nephrol* 2007, **18**(11):2960-2967.

82. Wang J, Huang L, Xu M, Yang L, Deng X, Li B: **Study on the Clinical Implications of NLR and PLR for Diagnosing Frailty in Maintenance Hemodialysis Patients and Their Correlations with Patient Prognosis**. *J Healthc Eng* 2022, **2022**.

83. Inoue T, Shinjo T, Matsuoka M, Tamashiro M, Oba K, Arasaki O, Moromizato T, Arima H: **The association between frailty and chronic kidney disease; cross-sectional analysis of the Nambu Cohort Study**. *CLINICAL AND EXPERIMENTAL NEPHROLOGY* 2021, **25**(12):1311-1318.

84. Zachciał J, Uchmanowicz I, Czapla M, Krajewska M, Banasik M: **The Association between Psychosocial and Age-Related Factors with Adherence to Immunosuppressive Therapies after Renal Transplantation**. *Journal of Clinical Medicine* 2022, **11**(9).

85. Mutevelić-Turković A, Resić H, Roljić BČ, Dervišević A, Bećiragić A: **The frailty phenotype in hemodialysis patients and its association with biochemical markers of mineral bone disorder, inflammation and nutrition**. *Rom J Intern Med* 2022, **60**(1):42-48.

86. Li Y, Zhang D, Ma Q, Diao Z, Liu S, Shi X: **The impact of frailty on prognosis in elderly hemodialysis patients: A prospective cohort study**. *Clin Interv Aging* 2021, **16**:1659-1667.

87. Chen CH, Hsieh YL, Chuang SY, Su FY, Wang KT, Luo CM, Meng SW, Wu CC: **The Impact of Frailty on the Outcomes of Hemodialysis Vascular Access**. *Acta Cardiol Sin* 2022, **38**(1):29-38.

88. Takeuchi H, Uchida HA, Kakio Y, Okuyama Y, Okuyama M, Umebayashi R, Wada K, Sugiyama H, Sugimoto K, Rakugi H *et al*: **The Prevalence of Frailty and its Associated Factors in Japanese Hemodialysis Patients**. *Aging Dis* 2018, **9**(2):192-207.

89. Gopinathan JC, Hafeeq B, Aziz F, Narayanan S, Aboobacker IN, Uvais NA: **The Prevalence of Frailty and its Association with Cognitive Dysfunction among Elderly Patients on Maintenance Hemodialysis: A Cross-Sectional Study from South India**. *Saudi J Kidney Dis Transpl* 2020, **31**(4):767-774.

90. Jegatheswaran J, Chan R, Hiremath S, Moorman D, Suri RS, Ramsay T, Zimmerman D: **Use of the FRAIL Questionnaire in Patients With End-Stage Kidney Disease**. *Can J Kidney Health Dis* 2020, **7**:2054358120952904.

91. Lorenz EC, Cosio FG, Bernard SL, Bogard SD, Bjerke BR, Geissler EN, Hanna SW, Kremers WK, Cheng Y, Stegall MD *et al*: **The Relationship Between Frailty and Decreased Physical Performance With Death on the Kidney Transplant Waiting List**. *Prog Transplant* 2019, **29**(2):108-114.

92. Miller LM, Rifkin D, Lee A, Tamura MK, Pajewski NM, Weiner DE, Al-Rousan T, Shlipak M, Ix JH: **Association of Urine Biomarkers of Kidney Tubule Injury and Dysfunction With Frailty Index and Cognitive Function in Persons With CKD in SPRINT**. *AMERICAN JOURNAL OF KIDNEY DISEASES* 2021, **78**(4):530-U559.

93. Saitoh M, Ogawa M, Kondo H, Suga K, Takahashi T, Itoh H, Tabata Y: **Bioelectrical impedance analysis-derived phase angle as a determinant of protein-energy wasting and frailty in maintenance hemodialysis patients: retrospective cohort study**. *BMC Nephrol* 2020, **21**(1):438.

94. Hendra H, Sridharan S, Farrington K, Davenport A: **Characteristics of Frailty in Haemodialysis Patients**. *Gerontol Geriatr Med* 2022, **8**:23337214221098889.

95. Davenport A: **Comparison Between the Physical Performance Test and the Clinical Frailty Score in Adult Patients With Chronic Kidney Disease Treated by Haemodialysis**. *Gerontol Geriatr Med* 2022, **8**:23337214221085875.

96. Imamura K, Yamamoto S, Suzuki Y, Yoshikoshi S, Harada M, Osada S, Kamiya K, Matsuzawa R, Matsunaga A: **Comparison of the association between six different frailty scales and clinical events in patients on hemodialysis**. *Nephrol Dial Transplant* 2022.

97. Anderson BM, Qasim M, Correa G, Evison F, Gallier S, Ferro CJ, Jackson TA, Sharif A: **Correlations, agreement and utility of frailty instruments in prevalent haemodialysis patients: baseline cohort data from the FITNESS study**. *Clin Kidney J* 2022, **15**(1):145-152.

98. Hilbrands LB, Duivenvoorden R, Vart P, Franssen C, Hemmelder MH, Jager KJ, Kieneker LM, Noordzij M, Pena MJ, Vries H *et al*: **COVID-19-related mortality in kidney transplant and dialysis patients: results of the ERACODA collaboration**. *Nephrol Dial Transplant* 2020, **35**(11):1973-1983.

99. Kakio Y, Uchida HA, Takeuchi H, Okuyama Y, Okuyama M, Umebayashi R, Wada K, Sugiyama H, Sugimoto K, Rakugi H *et al*: **Diabetic nephropathy is associated with frailty in patients with chronic hemodialysis**. *Geriatr Gerontol Int* 2018, **18**(12):1597-1602.

100. Tabinor M, Crowley LE, Godlee A, Flanagan D, Rashid RM, Baharani J, Ferro CJ, Eddington H: **End-stage kidney disease patients from ethnic minorities and mortality in coronavirus disease 2019**. *Hemodial Int* 2022, **26**(1):83-93.

101. Yuan H, Zhang Y, Xue G, Yang Y, Yu S, Fu P: **Exploring psychosocial factors associated with frailty incidence among patients undergoing maintenance hemodialysis**. *J Clin Nurs* 2020, **29**(9-10):1695-1703.

102. Gesualdo GD, Duarte JG, Zazzetta MS, Kusumota L, Orlandi FS: **Frailty and associated risk factors in patients with chronic kidney disease on dialysis**. *Cien Saude Colet* 2020, **25**(11):4631-4637.

103. Chi CY, Lee SY, Chao CT, Huang JW: **Frailty as an Independent Risk Factor for Depression in Patients With End-Stage Renal Disease: A Cross-Sectional Study**. *Front Med (Lausanne)* 2022, **9**:799544.

104. Garcia-Canton C, Rodenas A, Lopez-Aperador C, Rivero Y, Anton G, Monzon T, Diaz N, Vega N, Loro JF, Santana A *et al*: **Frailty in hemodialysis and prediction of poor short-term outcome: mortality, hospitalization and visits to hospital emergency services**. *Ren Fail* 2019, **41**(1):567-575.

105. Soldati A, Poggi MM, Azzolino D, Vettoretti S, Cesari M: **Frailty index and adverse outcomes in older patients in haemodialysis**. *Arch Gerontol Geriatr* 2022, **101**:104673.

106. Kosoku A, Uchida J, Iwai T, Shimada H, Kabei K, Nishide S, Maeda K, Kabata D, Shintani A, Nakatani T: **Frailty is associated with dialysis duration before transplantation in kidney transplant recipients: A Japanese single-center cross-sectional study**. *Int J Urol* 2020, **27**(5):408-414.

107. Dos SMM, Coelho DCN, Archangelo TE, Modelli DAL, Pires FFS, de Souza CR, Kawano PR, Papini SJ, Costa NA, Monteiro DBAR: **Frailty predicts surgical complications after kidney transplantation. A propensity score matched study**. *PLoS One* 2020, **15**(2):e0229531.

108. Wei Y, Wan Y, Zhang Y, Zhang C, Cao Y: **Frailty prevalence and associated factors in patients on maintenance hemodialysis**. *Int J Clin Exp Med* 2021, **14**(6):2002-2009.

109. Neradova A, Vajgel G, Hendra H, Antonelou M, Kostakis ID, Wright D, Masson P, Milne SE, Jones G, Salama A *et al*: **Frailty score before admission as risk factor for mortality of renal patients during the first wave of the COVID pandemic in London**. *G Ital Nefrol* 2021, **38**(3).

110. Bao Y, Dalrymple L, Chertow GM, Kaysen GA, Johansen KL: **Frailty, dialysis initiation, and mortality in end-stage renal disease**. *Arch Intern Med* 2012, **172**(14):1071-1077.

111. Zhou H, Al-Ali F, Rahemi H, Kulkarni N, Hamad A, Ibrahim R, Talal TK, Najafi B: **Hemodialysis Impact on Motor Function beyond Aging and Diabetes-Objectively Assessing Gait and Balance by Wearable Technology**. *Sensors (Basel, Switzerland)* 2018, **18**(11).

112. Chan GC, Ng JK, Chow KM, Kwong VW, Pang WF, Cheng PM, Law MC, Leung CB, Li PK, Szeto CC: **Impact of frailty and its inter-relationship with lean tissue wasting and malnutrition on kidney transplant waitlist candidacy and delisting**. *Clin Nutr* 2021, **40**(11):5620-5629.

113. Kumarasinghe AP, Chakera A, Chan K, Dogra S, Broers S, Maher S, Inderjeeth C, Jacques A: **Incorporating the Clinical Frailty Scale into routine outpatient nephrology practice: an observational study of feasibility and associations**. *Intern Med J* 2021, **51**(8):1269-1277.

114. Jiang X, Li D, Shen W, Shen X, Liu Y: **In-Hospital Outcomes of Patients on Maintenance Dialysis With Frailty: 10-year Results From the US National Inpatient Sample Database**. *Journal of renal nutrition : the official journal of the Council on Renal Nutrition of the National Kidney Foundation* 2020, **30**(6):526-534.

115. Thomas AG, Ruck JM, Chu NM, Agoons D, Shaffer AA, Haugen CE, Swenor B, Norman SP, Garonzik-Wang J, Segev DL *et al*: **Kidney transplant outcomes in recipients with visual, hearing, physical and walking impairments: a prospective cohort study**. *NEPHROLOGY DIALYSIS TRANSPLANTATION* 2020, **35**(7):1262-1270.

116. Tylicki L, Puchalska-Reglińska E, Tylicki P, Och A, Polewska K, Biedunkiewicz B, Parczewska A, Szabat K, Wolf J, Dębska-Ślizień A: **Predictors of Mortality in Hemodialyzed Patients after SARS-CoV-2 Infection**. *J Clin Med* 2022, **11**(2).

117. Yi C, Lin J, Cao P, Chen J, Zhou T, Yang R, Lu S, Yu X, Yang X: **Prevalence and Prognosis of Coexisting Frailty and Cognitive Impairment in Patients on Continuous Ambulatory Peritoneal Dialysis**. *Sci Rep* 2018, **8**(1):17305.

118. Haugen CE, Thomas AG, Chu NM, Shaffer AA, Norman SP, Bingaman AW, Segev DL, McAdams-DeMarco M: **Prevalence of frailty among kidney transplant candidates and recipients in the United States: Estimates from a National Registry and Multicenter Cohort Study**. *Am J Transplant* 2020, **20**(4):1170-1180.

119. Worthen G, Vinson A, Cardinal H, Doucette S, Gogan N, Gunaratnam L, Keough-Ryan T, Kiberd BA, Prasad B, Rockwood K *et al*: **Prevalence of Frailty in Patients Referred to the Kidney Transplant Waitlist**. *Kidney360* 2021, **2**(8):1287-1295.

120. Wu H, Van Mierlo R, McLauchlan G, Challen K, Mitra S, Dhaygude AP, Nixon AC: **Prognostic performance of clinical assessment tools following hip fracture in patients with chronic kidney disease**. *Int Urol Nephrol* 2021, **53**(11):2359-2367.

121. Iyasere O, Brown EA, Johansson L, Davenport A, Farrington K, Maxwell AP, Collinson H, Fan S, Habib AM, Stoves J *et al*: **Quality of life with conservative care compared with assisted peritoneal dialysis and haemodialysis**. *Clin Kidney J* 2019, **12**(2):262-268.

122. Shrestha P, Haugen CE, Chu NM, Shaffer A, Garonzik-Wang J, Norman SP, Walston JD, Segev DL, McAdams-DeMarco MA: **Racial differences in inflammation and outcomes of aging among kidney transplant candidates**. *BMC Nephrol* 2019, **20**(1):176.

123. Lee S, Lee S, Harada K, Bae S, Makizako H, Doi T, Tsutsumimoto K, Hotta R, Nakakubo S, Park H *et al*: **Relationship between chronic kidney disease with diabetes or hypertension and frailty in community-dwelling Japanese older adults**. *GERIATRICS & GERONTOLOGY INTERNATIONAL* 2017, **17**(10):1527-1533.

124. Santos D, Pallone JM, Manzini C, Zazzetta MS, Orlandi FS: **Relationship between frailty, social support and family functionality of hemodialysis patients: a cross-sectional study**. *Sao Paulo Med J* 2021, **139**(6):570-575.

125. Heybeli C, Kazancioglu R, Smith L, Veronese N, Soysal P: **Risk factors for high fall risk in elderly patients with chronic kidney disease**. *INTERNATIONAL UROLOGY AND NEPHROLOGY* 2021.

126. Carvalho TC, Dini AP: **Risk of falls in people with chronic kidney disease and related factors**. *Rev Lat Am Enfermagem* 2020, **28**:e3289.

127. Fu W, Zhang A, Ma L, Jia L, Chhetri JK, Chan P: **Severity of frailty as a significant predictor of mortality for hemodialysis patients: a prospective study in China**. *Int J Med Sci* 2021, **18**(14):3309-3317.

128. Demircioglu DT: **The Association of Vitamin D Levels and the Frailty Phenotype Among Non-geriatric Dialysis Patients: A Cross-sectional Study**. *Clinics (Sao Paulo)* 2018, **73**:e116.

129. Jafari M, Kour K, Giebel S, Omisore I, Prasad B: **The Burden of Frailty on Mood, Cognition, Quality of Life, and Level of Independence in Patients on Hemodialysis: Regina Hemodialysis Frailty Study**. *Can J Kidney Health Dis* 2020, **7**.

130. Schaenman J, Castellon L, Liang EC, Nanayakkara D, Abdalla B, Sarkisian C, Goldwater D: **The Frailty Risk Score predicts length of stay and need for rehospitalization after kidney transplantation in a retrospective cohort: a pilot study**. *Pilot Feasibility Stud* 2019, **5**:144.

131. Brar R, Whitlock R, Komenda P, Lerner B, Prasad B, Bohm C, Thorsteinsdottir B, Rigatto C, Tangri N: **The Impact of Frailty on Technique Failure and Mortality in Patients on Home Dialysis**. *Perit Dial Int* 2019, **39**(6):532-538.

132. van Loon IN, Joosten H, Iyasere O, Johansson L, Hamaker ME, Brown EA: **The prevalence and impact of falls in elderly dialysis patients: Frail elderly Patient Outcomes on Dialysis (FEPOD) study**. *Arch Gerontol Geriatr* 2019, **83**:285-291.

133. Zhang B, Zhao P, Wang H, Wang S, Wei C, Gao F, Liu H: **Factors associated with frailty in kidney transplant recipients: A cross-sectional study**. *J Ren Care* 2021.

134. Gong W, Yao L, Zhong X, Qin D, Huang C, Yin L, Liu F: **Prevalence and associated factors of frailty among Southern Chinese Han patients on haemodialysis: a multicentre, observational cross-sectional study**. *BMJ Open* 2022, **12**(3):e054177.

135. Montesanto A, De Rango F, Berardelli M, Mari V, Lattanzio F, Passarino G, Corsonello A: **Glomerular filtration rate in the elderly and in the oldest old: correlation with frailty and mortality**. *Age (Dordr)* 2014, **36**(3):9641.

136. Rodriguez Villarreal I, Ortega O, Hinostroza J, Cobo G, Gallar P, Mon C, Herrero JC, Ortiz M, Di Giogia C, Oliet A *et al*: **Geriatric assessment for therapeutic decision-making regarding renal replacement in elderly patients with advanced chronic kidney disease**. *Nephron Clin Pract* 2014, **128**(1-2):73-78.

137. Shlipak MG, Stehman-Breen C, Fried LF, Song X, Siscovick D, Fried LP, Psaty BM, Newman AB: **The presence of frailty in elderly persons with chronic renal insufficiency**. *Am J Kidney Dis* 2004, **43**(5):861-867.

138. Yadla M, John JP, Mummadi M: **A study of clinical assessment of frailty in patients on maintenance hemodialysis supported by cashless government scheme**. *Saudi J Kidney Dis Transpl* 2017, **28**(1):15-22.

139. Painter P, Kuskowski M: **A closer look at frailty in ESRD: getting the measure right**. *Hemodial Int* 2013, **17**(1):41-49.

140. Johansen KL, Dalrymple LS, Delgado C, Kaysen GA, Kornak J, Grimes B, Chertow GM: **Association between body composition and frailty among prevalent hemodialysis patients: a US Renal Data System special study**. *J Am Soc Nephrol* 2014, **25**(2):381-389.

141. Ghazi L, Yaffe K, Tamura MK, Rahman M, Hsu CY, Anderson AH, Cohen JB, Fischer MJ, Miller ER, Navaneethan SD *et al*: **Association of 24-hour ambulatory blood pressure patterns with cognitive function and physical functioning in CKD**. *CLINICAL JOURNAL OF THE AMERICAN SOCIETY OF NEPHROLOGY* 2020, **15**(4):452-464.

142. Goto NA, van Loon IN, Boereboom F, Emmelot-Vonk MH, Willems HC, Bots ML, Gamadia LE, van Bommel E, Van de Ven P, Douma CE *et al*: **Association of Initiation of Maintenance Dialysis with Functional Status and Caregiver Burden**. *CLINICAL JOURNAL OF THE AMERICAN SOCIETY OF NEPHROLOGY* 2019, **14**(7):1039-1047.

143. Johansen KL, Dalrymple LS, Glidden D, Delgado C, Kaysen GA, Grimes B, Chertow GM: **Association of Performance-Based and Self-Reported Function-Based Definitions of Frailty with Mortality among Patients Receiving Hemodialysis**. *Clin J Am Soc Nephrol* 2016, **11**(4):626-632.

144. McAdams-DeMarco MA, Isaacs K, Darko L, Salter ML, Gupta N, King EA, Walston J, Segev DL: **Changes in Frailty After Kidney Transplantation**. *J Am Geriatr Soc* 2015, **63**(10):2152-2157.

145. Johansen KL, Painter P, Delgado C, Doyle J: **Characterization of physical activity and sitting time among patients on hemodialysis using a new physical activity instrument**. *J Ren Nutr* 2015, **25**(1):25-30.

146. Chao CT, Wang J, Huang JW, Chan DC, Hung KY, Chien KL, COhort GNNC: **Chronic kidney disease-related osteoporosis is associated with incident frailty among patients with diabetic kidney disease: a propensity score-matched cohort study**. *Osteoporos Int* 2020, **31**(4):699-708.

147. Johansen KL, Dalrymple LS, Delgado C, Kaysen GA, Kornak J, Grimes B, Chertow GM: **Comparison of self-report-based and physical performance-based frailty definitions among patients receiving maintenance hemodialysis**. *Am J Kidney Dis* 2014, **64**(4):600-607.

148. Kutner NG, Zhang R, Allman RM, Bowling CB: **Correlates of ADL difficulty in a large hemodialysis cohort**. *Hemodial Int* 2014, **18**(1):70-77.

149. Chan GC, Ng JK, Chow KM, Kwan BC, Kwong VW, Pang WF, Cheng PM, Law MC, Leung CB, Li PK *et al*: **Depression does not predict clinical outcome of Chinese peritoneal Dialysis patients after adjusting for the degree of frailty**. *BMC Nephrol* 2020, **21**(1):329.

150. Sy J, McCulloch CE, Johansen KL: **Depressive symptoms, frailty, and mortality among dialysis patients**. *HEMODIALYSIS INTERNATIONAL* 2019, **23**(2):239-246.

151. Kang SH, Do JY, Lee SY, Kim JC: **Effect of dialysis modality on frailty phenotype, disability, and health-related quality of life in maintenance dialysis patients**. *PLOS ONE* 2017, **12**(5).

152. Chao CT, Huang JW, Chan DC: **Frail phenotype might herald bone health worsening among end-stage renal disease patients**. *PeerJ* 2017, **2017**(7).

153. Garonzik-Wang JM, Govindan P, Grinnan JW, Liu M, Ali HM, Chakraborty A, Jain V, Ros RL, James NT, Kucirka LM *et al*: **Frailty and delayed graft function in kidney transplant recipients**. *Arch Surg* 2012, **147**(2):190-193.

154. McAdams-DeMarco MA, Law A, Salter ML, Chow E, Grams M, Walston J, Segev DL: **Frailty and early hospital readmission after kidney transplantation**. *Am J Transplant* 2013, **13**(8):2091-2095.

155. McAdams-DeMarco MA, Suresh S, Law A, Salter ML, Gimenez LF, Jaar BG, Walston JD, Segev DL: **Frailty and falls among adult patients undergoing chronic hemodialysis: a prospective cohort study**. *BMC Nephrol* 2013, **14**:224.

156. Walker SR, Brar R, Eng F, Komenda P, Rigatto C, Prasad B, Bohm CJ, Storsley LJ, Tangri N: **Frailty and physical function in chronic kidney disease: the CanFIT study**. *Can J Kidney Health Dis* 2015, **2**:32.

157. Lee SY, Wang J, Chao CT, Chien KL, Huang JW: **Frailty is associated with a higher risk of developing delirium and cognitive impairment among patients with diabetic kidney disease: A longitudinal population-based cohort study**. *DIABETIC MEDICINE* 2021, **38**(7).

158. McAdams-DeMarco MA, Law A, Tan JW, Delp C, King EA, Orandi B, Salter M, Alachkar N, Desai N, Grams M *et al*: **Frailty, Mycophenolate Reduction, and Graft Loss in Kidney Transplant Recipients**. *TRANSPLANTATION* 2015, **99**(4):805-810.

159. Goto NA, van Loon IN, Morpey MI, Verhaar MC, Willems HC, Emmelot-Vonk MH, Bots ML, Boereboom F, Hamaker ME: **Geriatric Assessment in Elderly Patients with End-Stage Kidney Disease**. *NEPHRON* 2019, **141**(1):41-48.

160. Chao CT, Huang JW: **Geriatric syndromes are potential determinants of the medication adherence status in prevalent dialysis patients**. *PeerJ* 2016, **4**:e2122.

161. McAdams-DeMarco MA, Ying H, Olorundare I, King EA, Haugen C, Buta B, Gross AL, Kalyani R, Desai NM, Dagher NN *et al*: **Individual Frailty Components and Mortality in Kidney Transplant Recipients**. *TRANSPLANTATION* 2017, **101**(9):2126-2132.

162. Chu NM, Ruck J, Chen X, Xue QL, Norman SP, Segev DL, McAdams-DeMarco MA: **Long-term Trajectories of Frailty and its Components after Kidney Transplantation**. *J Gerontol A Biol Sci Med Sci* 2022.

163. Chiang JM, Kaysen GA, Segal M, Chertow GM, Delgado C, Johansen KL: **Low testosterone is associated with frailty, muscle wasting and physical dysfunction among men receiving hemodialysis: a longitudinal analysis**. *Nephrol Dial Transplant* 2019, **34**(5):802-810.

164. Lee SY, Wang J, Tsai HB, Chao CT, Chien KL, Huang JW: **Muscle relaxant use and the associated risk of incident frailty in patients with diabetic kidney disease: a longitudinal cohort study**. *THERAPEUTIC ADVANCES IN DRUG SAFETY* 2021, **12**.

165. Haugen CE, Agoons D, Chu NM, Liyanage L, Long J, Desai NM, Norman SP, Brennan DC, Segev DL, McAdams-DeMarco M: **Physical Impairment and Access to Kidney Transplantation**. *Transplantation* 2020, **104**(2):367-373.

166. Reese PP, Cappola AR, Shults J, Townsend RR, Gadegbeku CA, Anderson C, Baker JF, Carlow D, Sulik MJ, Lo JC *et al*: **Physical performance and frailty in chronic kidney disease**. *AMERICAN JOURNAL OF NEPHROLOGY* 2013, **38**(4):307-315.

167. Kimura H, Kalantar-Zadeh K, Rhee CM, Streja E, Sy J: **Polypharmacy and Frailty among Hemodialysis Patients**. *NEPHRON* 2021, **145**(6):624-632.

168. Kutner NG, Zhang R, Huang Y, McClellan WM, Soltow QA, Lea J: **Risk factors for frailty in a large prevalent cohort of hemodialysis patients**. *Am J Med Sci* 2014, **348**(4):277-282.

169. Chao CT, Hsu YH, Chang PY, He YT, Ueng RS, Lai CF, Chiang CK, Huang JW, Huang SJ: **Simple self-report FRAIL scale might be more closely associated with dialysis complications than other frailty screening instruments in rural chronic dialysis patients**. *Nephrology (Carlton)* 2015, **20**(5):321-328.

170. Kang SH, Do JY, Jeong HY, Lee SY, Kim JC: **The Clinical Significance of Physical Activity in Maintenance Dialysis Patients**. *Kidney Blood Press Res* 2017, **42**(3):575-586.

171. Kang SH, Do JY, Kim JC: **The relationship between disability and clinical outcomes in maintenance dialysis patients**. *Yeungnam Univ J Med* 2021, **38**(2):127-135.

172. Haugen CE, Gross A, Chu NM, Norman SP, Brennan DC, Xue QL, Walston J, Segev DL, McAdams-DeMarco M: **Development and Validation of an Inflammatory-Frailty Index for Kidney Transplantation**. *J Gerontol A Biol Sci Med Sci* 2021, **76**(3):470-477.

173. Chao CT, Lai HJ, Tsai HB, Yang SY, Huang JW: **Frail phenotype is associated with distinct quantitative electroencephalographic findings among end-stage renal disease patients: an observational study**. *BMC GERIATRICS* 2017, **17**(1):277.

174. Pérez-Sáez MJ, Arias-Cabrales CE, Dávalos-Yerovi V, Redondo D, Faura A, Vera M, Bach A, Pedreira G, Junyent E, Crespo M *et al*: **Frailty among chronic kidney disease patients on the kidney transplant waiting list: the sex-frailty paradox**. *Clin Kidney J* 2022, **15**(1):109-118.

175. Johansen KL, Delgado C, Kaysen GA, Chertow GM, Chiang J, Dalrymple LS, Segal MR, Grimes BA: **Frailty Among Patients Receiving Hemodialysis: Evolution of Components and Associations With Mortality**. *J Gerontol A Biol Sci Med Sci* 2019, **74**(3):380-386.

176. Chu NM, Gross AL, Shaffer AA, Haugen CE, Norman SP, Xue QL, Sharrett AR, Carlson MC, Bandeen-Roche K, Segev DL *et al*: **Frailty and Changes in Cognitive Function after Kidney Transplantation**. *J Am Soc Nephrol* 2019, **30**(2):336-345.

177. McAdams-DeMarco MA, Law A, King E, Orandi B, Salter M, Gupta N, Chow E, Alachkar N, Desai N, Varadhan R *et al*: **Frailty and Mortality in Kidney Transplant Recipients**. *AMERICAN JOURNAL OF TRANSPLANTATION* 2015, **15**(1):149-154.

178. McAdams-DeMarco MA, Law A, Salter ML, Boyarsky B, Gimenez L, Jaar BG, Walston JD, Segev DL: **Frailty as a Novel Predictor of Mortality and Hospitalization in Individuals of All Ages Undergoing Hemodialysis**. *JOURNAL OF THE AMERICAN GERIATRICS SOCIETY* 2013, **61**(6):896-901.

179. Pérez-Sáez MJ, Dávalos-Yerovi V, Redondo-Pachón D, Arias-Cabrales CE, Faura A, Bach A, Buxeda A, Burballa C, Junyent E, Nogués X *et al*: **Frailty in kidney transplant candidates: a comparison between physical frailty phenotype and FRAIL scales**. *J Nephrol* 2022.

180. Nixon AC, Bampouras TM, Pendleton N, Mitra S, Brady ME, Dhaygude AP: **Frailty is independently associated with worse health-related quality of life in chronic kidney disease: a secondary analysis of the Frailty Assessment in Chronic Kidney Disease study (vol 13, pg 85, 2020)**. *CLINICAL KIDNEY JOURNAL* 2021, **14**(3):1035-1035.

181. Lee SY, Wang J, Chao CT, Chien KL, Huang JW: **Frailty modifies the association between opioid use and mortality in chronic kidney disease patients with diabetes: a population-based cohort study**. *Aging (Albany NY)* 2020, **12**(21):21730-21746.

182. Chan GCK, Ng JKC, Chow KM, Kwong VWK, Pang WF, Cheng PMS, Law MC, Leung CB, Li PKT, Szeto CC: **Interaction between central obesity and frailty on the clinical outcome of peritoneal dialysis patients**. *PLoS ONE* 2020, **15**(10).

183. Thomas AG, Ruck JM, Shaffer AA, Haugen CE, Ying H, Warsame F, Chu N, Carlson MC, Gross AL, Norman SP *et al*: **Kidney Transplant Outcomes in Recipients With Cognitive Impairment: A National Registry and Prospective Cohort Study**. *TRANSPLANTATION* 2019, **103**(7):1504-1513.

184. Chu NM, Shi Z, Berkowitz R, Haugen CE, Garonzik-Wang J, Norman SP, Humbyrd C, Segev DL, McAdams-DeMarco MA: **Poor Outcomes in Kidney Transplant Candidates and Recipients With History of Falls**. *TRANSPLANTATION* 2020, **104**(8):1738-1745.

185. Nastasi AJ, McAdams-DeMarco MA, Schrack J, Ying H, Olorundare I, Warsame F, Mountford A, Haugen CE, González Fernández M, Norman SP *et al*: **Pre-Kidney Transplant Lower Extremity Impairment and Post-Kidney Transplant Mortality**. *AMERICAN JOURNAL OF TRANSPLANTATION* 2018, **18**(1):189-196.

186. Sy J, Streja E, Grimes B, Johansen KL: **The Marginal Cost of Frailty Among Medicare Patients on Hemodialysis**. *Kidney Int Rep* 2020, **5**(3):289-295.

187. Zanotto T, Mercer TH, van der Linden ML, Rush R, Traynor JP, Petrie CJ, Doyle A, Chalmers K, Allan N, Shilliday I *et al*: **The relative importance of frailty, physical and cardiovascular function as exercise-modifiable predictors of falls in haemodialysis patients: a prospective cohort study**. *BMC Nephrol* 2020, **21**(1):99.
